# Exposure to the widely used herbicide atrazine results in deregulation of global tissue-specific RNA transcription in the third generation and is associated with a global decrease of histone trimethylation in mice

**DOI:** 10.1093/nar/gkw840

**Published:** 2016-09-20

**Authors:** Chunxiang Hao, Aurore Gely-Pernot, Christine Kervarrec, Melissa Boudjema, Emmanuelle Becker, Pavel Khil, Sergei Tevosian, Bernard Jégou, Fatima Smagulova

**Affiliations:** 1 Inserm U1085 IRSET, 9 Avenue du Professeur Léon-Bernard, 35000 Rennes, France; 2 EHESP, 2 Avenue du Professeur Léon-Bernard, 35000 Rennes, France; 3 Clinical Center, National Institute of Health, Bethesda, MD 20892, USA; 4 University of Florida, Department of Physiological Sciences, Box 100144, 1333 Center Drive, 32610 Gainesville, FL, USA

## Abstract

The epigenetic events imposed during germline reprogramming and affected by harmful exposure can be inherited and transferred to subsequent generations via gametes inheritance. In this study, we examine the transgenerational effects promoted by widely used herbicide atrazine (ATZ). We exposed pregnant outbred CD1 female mice and the male progeny was crossed for three generations with untreated females. We demonstrate here that exposure to ATZ affects meiosis, spermiogenesis and reduces the spermatozoa number in the third generation (F3) male mice. We suggest that changes in testis cell types originate from modified transcriptional network in undifferentiated spermatogonia. Importantly, exposure to ATZ dramatically increases the number of transcripts with novel transcription initiation sites, spliced variants and alternative polyadenylation sites. We found the global decrease in H3K4me3 occupancy in the third generation males. The regions with altered H3K4me3 occupancy in F3 ATZ-derived males correspond to altered H3K4me3 occupancy of F1 generation and 74% of changed peaks in F3 generation are associated with enhancers. The regions with altered H3K4me3 occupancy are enriched in SP family and WT1 transcription factor binding sites. Our data suggest that the embryonic exposure to ATZ affects the development and the changes induced by ATZ are transferred up to three generations.

## INTRODUCTION

Epigenetic factors, such as DNA methylation, non-coding RNAs and histone modifications are involved in the regulation of many cellular functions and developmental reprogramming (1). Epigenetic inheritance has been demonstrated in several species including worm (2), fly (3) and mouse species (4,5). Epigenetic inheritance can be modulated by environmental factors and transmitted to subsequent generations via germline cells (6,7).

Recently, a series of studies have shown that exposing the gestating mammalian female F0 to environmental factors during embryonic gonadal sex determination causes changes in DNA methylation that are retained in adult F1–3 male progeny, which produce sperm with altered DNA methylation (8–12). The inherited changes were monitored in the third generation after treatment (13–15). Effects on DNA methylation reprogramming were suggested as a major mechanism for transgenerational inheritance of chromatin modifications.

In addition to DNA methylation, it was suggested that histone modifications could also play a role in epigenetic inheritance. The evidence for this mechanism comes from histone retention studies in sperm, with 10% of the haploid genome in humans and 1% in mice being able to retain the nucleosomes (16). These nucleosome-protected regions are of great interest because they can potentially mediate transgenerational epigenetic inheritance. The first high-resolution genome-wide map of human spermatozoa histones shows a strong enrichment in methylated histone H3K4me3 at many promoters of developmental genes, genes encoding signaling factors, imprinted gene clusters, microRNA and HOX gene clusters (17). These studies showed that the promoters in sperm are enriched in H3K4me3 and H3K27me3, and it was suggested that genes with these marks play a particularly important role in epigenetic inheritance (16–18). On the other hand, more recent nucleosome mapping studies showed that sperm nucleosome fraction is enriched in gene-poor regions and is generally depleted from the promoters (19,20). The discrepancy in nucleosome mapping could be explained by different chromatin preparation protocols (21). Despite different interpretation of histone position data, it is well agreed upon that the nucleosome-containing fraction in sperm (a fraction of genome that is not packed by protamines) plays important role in transgenerational inheritance. It remains unclear how histone marks are re-established *de novo* after replication. During replication, parental histones re-associate locally with newly synthesized DNA in budding yeast (22). H3K27me3 marks appear to propagate via replication in *Drosophila* (23). However, whether this mechanism is conserved in vertebrates, is uncertain. A recent work showed that accessible chromatin in early mouse embryos is both of the maternal and paternal origins (24) and implicated paternal sperm histone function in early zygote. H3K4me3 is found to be strongly associated with both maternal and paternal genomes in mouse, rabbit and bovine zygote (25). The human sperm nucleosome fraction is enriched in Short Interspersed Nuclear Element (SINE) and Long Interspersed Element-1 (LINE) retroelements (20). It was suggested that sperm DNA containing repetitive elements plays a key role in pre-implantation processes (20). This view was recently challenged by Royo *et al.*, who showed that nucleosomal enrichment in repetitive sequences is resulted from biased sequence analyses (26). However, the role of retroelements in transgenerational inheritance was also demonstrated independently in case of Intracisternal A-particle retroelements (27). Recent work showed that the accessible chromatin landscape in early embryos is extensively shaped by transposable elements (24).

Nucleosomes in sperm are also enriched in CpG islands (18). Recently, a strong association between nucleosome retention at CpG islands in sperm and the establishment of unmethylated regions in the early mammalian embryo was shown (28). Regions containing unmethylated CpG islands in early embryos are strongly associated with H3K4me3 and, to a lesser degree, H3K27me3 in mature sperm (28). The authors suggested that the distribution of paternal nucleosomes in zygotes influences transcriptional activity in the embryo (28). The role of histones in transgenerational inheritance also was demonstrated in *Caenorhabditis elegans*, where H3K27me and PRC2 transmit a memory of repression across generations and during development (29). Another piece of evidence corroborating the role for histones in transgenerational inheritance came from the work that showed sperm to be epigenetically programmed to regulate the transcription of several embryonic genes (30). The disruption of histone methylation in the developing sperm impairs the health of the offspring (31). Taken together, these findings raise the possibility that histone marks, which are retained in sperm, play a key role in safekeeping epigenetic information for subsequent generations. A groundbreaking work by Skinner laboratory showed that the fetus undergoing gonadal sex determination is particularly vulnerable to environmental exposure that engenders changes in DNA methylation (11).

In this study, we examined the effects of herbicide atrazine (ATZ) on transgenerational inheritance. ATZ is the most common contaminant found in underground waters in the USA (32) and many other countries (33,34). ATZ is metabolized in mammalian organisms by Glutathione S-transferases and cytochrome P450 systems and affects the expression of *Star, Cyp19a1* and many other genes (35,36). A series of experiments with sperm and isolated mitochondria suggest that ATZ inhibits mitochondrial function through its action on F1–F0-adenosine triphosphate (ATP) synthase, which is a major cellular protein capable of binding to ATZ (37). Recent study showed that ATZ also affects DNA methylation in plants (38). In mammals, ATZ exposure affects many tissues, including testes (39–42), ovaries (43–47), brain (48,49), liver (50–54) and the presence of low dose metabolites of ATZ is associated with low birth weight in human (55).

We hypothesized that embryonic exposure to ATZ during the E6.5 to E15.5 developmental window will cause heritable epigenetic reprograming (modifications) and affect reproduction in subsequent generations. To test this hypothesis, we treated gestating outbred CD1 mice with ATZ and studied subsequent generations, F1 and F3. We examined H3K4me3 modifications in the testes and RNA expression in several tissues. We show here that embryonic exposure to ATZ globally changes tissue-specific RNA expression patterns, which become deregulated concomitant with the alterations in H3K4me3 marks. We suggest that H3K4me3 occupancy pattern in the F3 generation of ATZ-lineage is derived from embryonic exposure of F1 males and that the pattern of altered H3K4me3 peaks in F3 is preserved from the previous generations, however, the mechanism of this inheritance is not known. We believe this work to be the first comprehensive study that integrates genome-wide ChIP-seq and RNA-seq approaches in two generations (F1 and F3) of animals with the goal of understanding transgenerational inheritance upon toxicant exposure. Moreover, this study generates a large number of novel sequencing data for an outbred CD1 mouse strain that is commonly used in many studies.

## MATERIALS AND METHODS

### RNA isolation

Total RNA was prepared from fresh or frozen tissue using TRIzol reagent (Life Technologies) and RNA easy plus kit (Qiagen)

### Quantitative PCR

Reverse transcription of total RNA was completed using QuantiTect Reverse Transcription kit (Qiagen) according to the manufacturer's instructions. Applied Biosystems (ABI) 7500 real-time polymerase chain reaction (PCR) system was used for quantitative PCR. Duplicates of at least four independent experiments were performed. Statistical significance was calculated by Student's *t*-test. The qPCR-quantification of ChIP-seq experiments was performed as previously described (56). Equal amounts of ChIP and input DNA were used and quantitative PCR was performed using the ABI 7500 real-time PCR system. The copy number for each locus was calculated using ABI sequence detection software (SDS) version V2.0.5 using a standard curve. Enrichments were estimated as a ratio of the copy number in ChIP samples to the copy number in the input sample as a percentage. The average enrichment values of at least four independent experiments were compared, plotted and expressed as a fold change. The primer sequences used in this research are listed in the Supplemental information (SI) in Supplementary Table S13.

### Chromatin immunoprecipitation and high-throughput sequencing

Chromatin immunoprecipitation was conducted as previously described (42). An Illumina Hiseq2000 Genome Analyzer was used to perform massively parallel 50-bp sequencing in Single End mode. We sequenced two biological replicates per condition in multiplexing mode. The reads were demultiplexed and passed through quality control, at which point reads shorter than 50 nucleotides were removed. FastQ files were generated using a genomic platform in Rennes, France.

### Peak calling and differential-peak finding

Approximately 40 million tags were derived from the anti-H3K4me3 ChIP and input. The resulting sequences were quality filtered using the Sickle program (57) (https://github.com/najoshi/sickle) with –q33 and mapped back to the mouse mm9/Ensembl genome using Bowtie 1.0.0 with seed length 20. Only tags that passed the quality filter and mapped uniquely to the genome were used. ChIP enrichment was further verified using CHANCE (58). The H3K4me3 mark peaks were identified using MACS 2.0.1 (59) with two biological replicate samples, including corresponding input, shift-size window 73 bp, no model, with *P*-value threshold <10E−5. The peak regions determined by MACS were further divided into subpeak regions using PeakSplitter with default parameters (60). Two biological replicates were used for ChIP-seq and samples for each condition were analyzed independently. The set of peaks was verified at an irreproducible discovery rate of 0.05% (61) to confirm that the biological replicates were sufficiently similar for use in the analysis. The number of mapped reads was multiplied by a scale factor to normalize the total number of reads in different samples. To compare the H3K4me3 ChIP datasets of the ATZ-treated and control samples, differential peak calling was performed using several steps. First, from all the peaks called above, we selected the peaks that were reproducible in both biological replicates in the same conditions; second, we retained only peaks with average values above the 50th quintile; last, we selected the peaks with fold changes above 1.5 or 2 and False Discovery Rate (FDR) < 10%. Statistical significance was calculated using a Limma test (62). The annotation of significantly differential peaks was performed by CEAS (63). The results were visualized using the Integrative genomics viewer IGV version 2.3.36 (64).

### Gene ontology (GO) term analyses

Gene ontology (GO) term analyses were performed using DAVID v6.7 (65) with enrichment thresholds ease 0.05, kappa index 3.

### Functional annotation of ChIP-seq data

Functional annotation was performed by GREAT version 3.0.0 (66) with default parameters.

### Meme-ChIP motif search

For this analysis, we used the summits of 500-bp sequences from the differential peaks. Motif finding was performed using MEME-ChIP (67) with the default parameters. Identified motifs were compared with known motifs using TomTom (68) with the default parameters. FIMO (Find Individual Motif Occurrences) was used to scan for SP1/3/4 and WT1 motif-binding sites (69). Mammalian conservation scores for each motif-binding site were obtained from the PhastCons30wayPlacental table from the UCSC browser. Highly conserved binding sites (>0.70 conservation score) were retained as potential targets.

### RNA-Seq library sequencing

A strand-specific library preparation protocol for RNA sequencing was performed using the sequencing technological platform at IGBMC, Strasbourg, France. We used three biological replicates for each tissue, testis, liver and hypothalamus. The sequencing was performed in massive parallel sequencing paired end mode, and the size of the sequencing tag was 50 bp.

### RNA-Seq expression data processing

The reads in FASTQ format were processed for quality control using the FastQC tool (http://www.bioinformatics.babraham.ac.uk/projects/fastqc/). For the analysis of differentially expressed genes (DEGs), the quality-checked reads for each condition were processed using the TopHat version 2.0.12 package (70). The reads were mapped to the reference genome (*Mus musculus* Ensembl mm9 sequence), and the alignment files were generated as binary sequence alignment map (BAM) files. These files were used as the input for Cufflinks, a complementary method used to generate assembled transcripts for each condition and the abundance was evaluated using read data. The assembled transcripts were compared and annotated using Cuffmerge against the Ensembl (mm9) gene annotations (71). Mm9 gene annotation was used in this study for the comparative analysis of previously published datasets. These assemblies were used in Cuffquant and Cuffnorm from the Cufflinks 2.2.1 package to calculate the expression levels. To identify transcripts differentially expressed between the ATZ- and control-derived samples, we first selected the cases for which we obtained values greater than the 50th quantile of all values in at least one condition and then we filtered the transcripts with more than a 2-fold difference between the ATZ and control-derived samples. Finally, we employed a statistical test implemented in the R package, Limma, with the false-discovery rate set at less than 5% (62). RNA-Seq data were visualized using the Integrated Genomics Viewer (version 2.3.36) (64). The correlations between biological replicates are shown in Supplementary Table S1.

### LncRNA identification

Transcripts that mapped to known lncRNAs were retained. Transcripts defined by Cuffmerge as unknown that include at least 2 exons and are longer than 200 bp were considered as putative lncRNAs. These putative lncRNAs were filtered for potential coding ability using the Coding Potential Assessment Tool (CPAT) and Coding Potential Calculator (CPC) (72,73). Any transcript with a negative CPC score (‘non-coding’ and ‘non-coding weak’ categories) and a CPAT coding probability less than 40% was classified as a non-coding RNA.

### Classification as ‘enhancers’ or ‘promoters’ for regions occupied by H3K4me3 in testis

It has been suggested that the ratio between H3K4me3 and H3K4me1 indicates the tendency of the region to act as either a promoter or an enhancer (74). To classify the differential regions as enhancers or promoters, we used a published dataset, which includes both H3K4me3 and H3K4me1 ChIP-seq data of mouse embryonic stem cells (75). For each region with altered H3K4me3 occupancy, we calculated the intensities of H3K4me3 and H3K4me1 marks from mouse embryonic stem cells. The differential regions that show intensity of H3K4me3 higher than H3K4me1 were defined as ‘promoters’ and the differential regions with intensity H3K4me1 intensity higher than H3K4me3 were considered as ‘enhancers’.

The additional methods are described in SI in the ‘Methods’ section.

## RESULTS

### Research strategy

To evaluate the effects of environmental chemicals on epigenetic inheritance, gestating outbred CD1 strain mice were treated with ATZ during embryonic period E6.5 to E15.5. This exposure window corresponds to reprogramming in the germline when the genome is undergoing global demethylation and subsequent remethylation. The progeny of F1-exposed males were crossed with unrelated females for three generations. F1 (exposed) and F3 (unexposed) males were used for transgenerational inheritance analysis. We used ATZ at 100 mg/kg/day. This dose is commonly used in toxicological studies with ATZ (76,77). According to our data this dose does not causes gross effects on organ or body weight in outbred CD1 mice. We used at least three independent lineages for ATZ and control samples. We analyzed germline, testicular and somatic (liver and hypothalamus tissue) cells for RNA expression. To determine the contribution of epigenetic factors, specifically histone modifications, to transgenerational inheritance, testis tissue was analyzed to determine genome-wide H3K4me3 distribution in F1 and F3 ATZ- or control-derived males.

### Embryonic exposure to ATZ reduces the number of spermatozoa in the F3 generation without changing the morphology of the testes in F3

The morphological analysis of H&E stained sections did not reveal notable differences in testis architecture between ATZ-derived and vehicle-treated F3 males (Supplementary Figure S1). To evaluate whether the relative proportions of germ and Sertoli cells were altered, we immunostained the testis sections with antibodies against proteins specific to these cell types (ZBTB16 and GATA1, respectively, (Supplementary Figure S2A)). Marker analysis did not reveal any notable differences between the ATZ-derived and control samples in F3 males (Supplementary Figure S2B). To assay the relative contributions of all cell types in the testes, we sorted the cells using FACS. Our data showed that ATZ treatment does not affect the relative proportion of cell types in the testes (Supplementary Figure S3A–E). Furthermore, we did not observe any significant changes in body or organ weights (Supplementary Figure S4A and B) or any decrease in testosterone and Follicle-stimulating hormone (FSH) levels (Supplementary Figure S4C), which are the expected outcomes of ATZ treatment in adult males (42). We cannot exclude the possibility that a subtle phenotype exists in F3 ATZ-lineage males that is not detectable by these methods.

The number of spermatozoa is an important physiological parameter of normal testis function, so we counted the number of spermatozoa in the epididymis in each group of males. We found that the number of spermatozoa was significantly decreased in ATZ-derived F1 and F3 generation males compared to vehicle-treated control mice, with a decrease of nearly 30% in F3, suggesting disrupted meiosis or spermiogenesis (Supplementary Figure S4D). In summary, the embryonic exposure of F1 males to ATZ does not obviously affect the morphology or cell types in F3 testis tissues but results in a lower spermatozoa count.

### Embryonic exposure to ATZ affects meiosis and spermiogenesis in F3 males

We hypothesized that the decrease in sperm number could be due to defects in meiosis or spermiogenesis. First, we asked whether ATZ exposure negatively affects meiosis in F3 male progeny of the treated male. We performed surface spreads from testis tissue and analyzed synaptonemal complexes (SCs) formation. SCs are zipper-like structures, which are assembled between homologous chromosomes during the prophase of the meiosis I. Formation of SCs is essential for proper parental chromosome segregation before meiotic division (78). We immunostained the surface cell spreads with antibodies against SYCP3 (a marker of the chromosome core) and SYCP1 (that marks completely synapsed chromosomes) and analyzed the pattern of chromosome synapsing. In normal meiosis during pachytene stage all chromosomes are totally synapsed, so SYCP1 protein is detected along each of the chromosomes. The analysis of SCs in F3 ATZ males showed that there are several defects in synapsing, specifically, telomere-to-telomere connections, formation of ring-like structure between X- and Y-sex chromosomes and incomplete synapsing (Supplementary Figure S5A–C). The quantitative analysis (*n* = 199 in control and *n* = 195 in ATZ derived cells) showed the significant increase of meiotic defects in F3 male progeny of ATZ derived males (Supplementary Figure S5D). To confirm this phenotype we immunostained the spreads with antibody against TERF1 (Supplementary Figure S6). TERF1 protein binds to repeats in telomeres and protects them against the degradation (79). We found the appearance of clusters of TERF1 clusters in places of telomere connections in ATZ-derived samples. We suggest that the deregulation of telomere-protective function could be the reason of abnormal telomere connections during meiosis in ATZ lineage that we observed. Our data also suggest that meiosis is abnormal in F3 generation ATZ males and could lead to spermatozoa number decline.

To elucidate an ATZ effect on spermiogenesis we analyzed the protein level of protamine 2 from whole testis extract in F3 males. We found the protein levels of protamine 2 decreased 2.6 times in ATZ-derived testis (Supplementary Figure S7A and B). Recent study demonstrated that acetylation at histone H4 (H4K5ac and H4K8ac) is essential for bromodomain-containing protein (BRDT) binding, and that binding is, in turn, critical for histone-to-protamine transition (80). We analyzed the level of H4K5Ac in purified histone fraction by WB and we found that the level of H4K5Ac has decreased 1.3 times in F3 ATZ-lineage males (Supplementary Figure S7C and D). These results suggest that the decreased protamine and histone H4 acetylation levels could reflect the inefficient histone-to-protamine replacement in the F3 lineage of ATZ males.

In summary, our results demonstrate that meiosis and spermiogenesis are affected in F3 generation of ATZ lineage males and that could contribute to spermatozoa decline that we observe.

### RNA expression is globally affected in F3 animals

**Figure 1.**
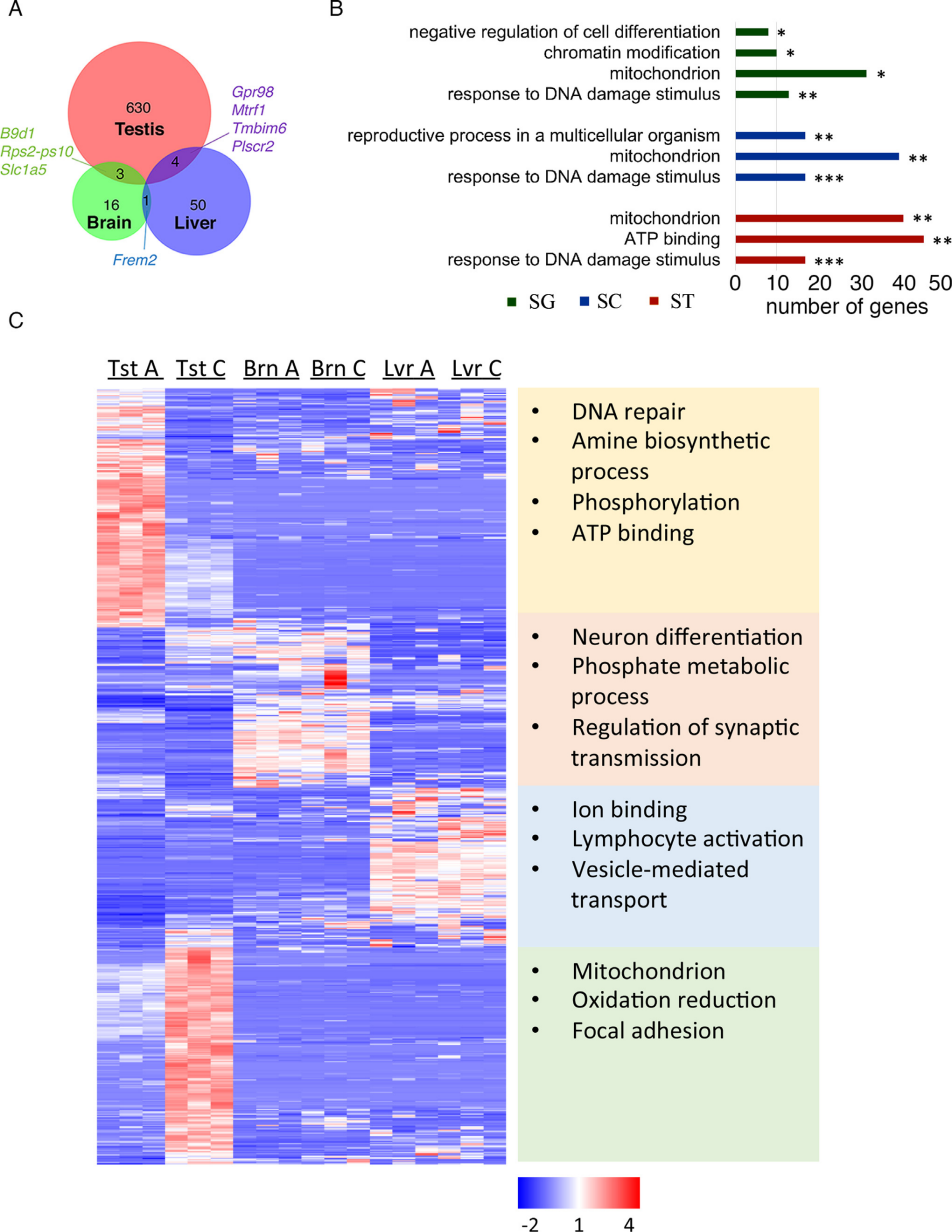
Embryonic exposure to ATZ globally affects gene expression in the third generation of males after treatment. (**A**) Venn diagram of the DEGs in ATZ F3 male testes, liver and brain. (**B**) The functional annotation of DEGs in testis fractions reveals several enriched clusters; gene ontology terms are sorted based on *P*-values (*P < 0.05; **P < 0.01, ***P < 0.001, Fisher exact test), SG-spermatogonia, SC-spermatocytes, ST-spermatids. The number of genes in each term is shown in right. (**C**) The heatmap of the expression of DEGs in the testes. The hierarchical cluster analysis groups the DEGs from the testes into four clusters according to their expression levels in three tissues. The corresponding enriched GO terms for each cluster are shown in the right. Each column represents a biological replicate. The upregulated genes are depicted in red, downregulated in blue. TstC (testis control samples), TstA (testis ATZ samples), BrnC (brain control samples), BrnA (brain ATZ samples), LvrC (liver control samples) and LvrA (liver ATZ samples).

To determine the consequences of ATZ treatment at whole genome level, we performed genome-wide transcriptomics analysis by strand specific paired-end high-throughput RNA sequencing. We identified a total of 704 genes (Figure 1A) corresponding to 1419 differentially expressed transcripts (fold change (FC) > 2 and FDR < 0.05): 1322 in the testes (Supplementary Figure S8 and Supplementary Table S2), 69 in the liver and 28 in the brain. Surprisingly, only a few DEGs were found to be common among all tissues. Thus, the majority of DEGs are tissue-specific, suggesting that transgenerational ATZ exposure alters different gene sets in each tissue. Among the tissues we tested, the testes showed the highest number of DEGs. We reasoned that such enrichment stems from testicular germ line where the transcripts are much more vulnerable to ATZ exposure than somatic transcripts. Genes encoding proteins with functions in spermatogenesis (Supplementary Figure S9) and DNA damage response (Supplementary Figure S10) were selected, and their differential expression was confirmed by qPCR. DNA damage related genes (*Nbn, Rif1, Sun1, Ctc1*) are also known to be involved in the regulation of telomere function (81–85). To determine whether telomere length is altered in ATZ-lineage males, we analyzed the average telomere length ratio (ATLR) in genomic DNA from the testes and liver using recently developed qPCR approach (86). We found that in both tissues from ATZ-lineage males, the ATLR is significantly increased by 50 and 18% respectively (Supplementary Figure S11). We concluded that telomere length is increased in the progeny of the exposed F1 males and that the resulting changes are detected in two different tissues in non-exposed generation (F3). The mechanism for the telomere length increase remains to be understood.

To identify the cellular origin for the DEGs we used recently published datasets of genes that are expressed in different testicular cell types (87). We identified 349 DEGs out of 14 985 (total number of genes) that originate from spermatogonia (SG), 422 DEGs out of 16 562 from spermatocytes, 465 DEGs out of 21 856 in round spermatids and 411 DEGs out of total 17 081 genes from Sertoli cells (Supplementary Figure S12A). All pre-meiotic (SG) and post-meiotic (spermatocytes and spermatids) cell fractions are enriched in genes associated with mitochondria and DNA repair function (Figure 1B). SG are enriched in genes associated with chromatin modification and negative regulation of cell differentiation. The meiotic (spermatocytes) fraction is enriched in genes associated with reproduction function. Spermatids are enriched in genes encoding ATP-binding proteins. We sought to test the idea that changes in the expression of (at least some) genes are associated with direct effects of ATZ. To examine whether DEGs identified in this study are encoding protein products capable of interacting with ATZ, we compared our DEGs roster with a database http://ctdbase.org/ (36) of proteins known to interact with ATZ (the database in (36) is based on experimental data). We noted that 157 DEGs (*P* = 4E-6, Hypergeometric test) that have altered function in ATZ-lineage males were also identified in other ATZ studies. The large numbers of these genes are associated with mitochondrial function. Specifically, these are genes encoding oxidative reduction enzymes (*Cryzl1, Cyp27a1, Fdxr, Hsd17b3, Nadsyn1, Ndufs2, Ndufs5*), genes of mitochondrial homeostasis (*Aars2, Atp5h, Bnip3, Chchd3, Cox18, Fdxr, Gcat, Htt, Mrpl55, Mtrf1, Ndufs2, Ndufs5, Polg, Pdk2, Tdrd7*). Many of ATZ-responsive DEGs are encoding ATP-binding proteins (*Aars2, Dhx34, Glul, Pdk2*). Importantly, the expression level of *Atpaf2* (ATP synthase mitochondrial F1 complex assembly factor 2) encoding the ATP synthase assembly protein has dramatically (eight times) decreased. This data suggests that mitochondrial metabolism and ATP synthesis could be an important part of ATZ lineage phenotype.

This data and the work of others (80) have suggested the role for histone H4 acetylation in spermatogenesis and in early zygote (88). To further explore the connection between transgenerational inheritance, histone H4 acetylation and gene expression during chromatin remodeling, we compared DEGs identified in this study to a recently published datasets of H4K5ac and H4K8ac ChIP-seq data in separated round spermatids and spermatocytes fractions (80). We found that H4K5Ac marks are present in large number of DEGs: 511 and 495 testis DEGs in spermatocytes and spermatids, respectively. Similarly, 496 and 465 DEGs normally have H4K8Ac marks in spermatocytes and spermatids, respectively. Out of 637 of DEGs total, 80 and 78% have H4K5Ac and H4K8Ac marks, respectively. For a genome-wide comparison, only 61% genes expressed in testis have H4K5Ac marks. These are genes associated with chromosome segregation function (*P* = 5.40E-07, Fisher exact test) and genes associated with meiotic cell cycle (*P* = 7.55E-05) among the others. The genes without H4K5Ac marks are associated with sensory perception of chemical stimulus function (*P* = 3.83E-82) and with G-protein coupled receptor signaling pathway (*P* = 7.94E-68). These data provide further support for the possibility that histone H4 acetylation is altered at regions of DEGs in ATZ progeny male mice.

We noted that genes associated with mitochondrial function are also affected in the third generation in liver. The analysis of transcripts from liver revealed that many DEGs belong to the oxidoreductase (*Me1, Cyp2c44, Akr1b3, Glud1, Pgd, Cat, Dus1l, Hadha, Mtrf1, Slc25a22, Mtscp1, Timm23*) and to mitochondrion function (*Me1, Mtrf1, Glud1, Slc25a22, Mtcp1, Cat, Timm23, Hadha*) pathways (Supplementary Table S3). Similarly, among very few DEGs that were identified in brain, we noted genes associated with embryonic development (*B9d1*), oxidation-reduction (*Txn2, 4933434E20Rik*) and imprinted brain development (*Nnat*) (Supplementary Table S4).

To obtain a functional overview of the expression patterns for DEGs in the testes, brain and liver, we performed a hierarchical cluster analysis (Figure 1C). We determined that a subset of genes associated with DNA repair function was upregulated in the testis of F3 ATZ-lineage males, while the expression levels for these genes in the control testes, brain and liver were low. In contrast, a subset of genes associated with mitochondrion and oxidation-reduction processes showed a different pattern: they were downregulated in F3 ATZ-lineage males compared to the control testes, and their expression levels in the brain and liver were also low. In summary, the embryonic exposure to ATZ globally affects the transcriptomics in the third generation, with testis gene expression substantially more affected by the exposure than the two other organs examined.

### Embryonic exposure to ATZ is associated with differential expression of long non-coding RNA in the third generation

Among differentially expressed transcripts in the testes, 41% are non-coding RNAs (Supplementary Figure S13). Long non-coding RNAs play essential roles in many developmental processes, diseases and cancer (89–91). To compare the expression of the long intergenic non-coding RNAs we analyzed the transcripts that are longer than 200 bp, include at least two exons and have no coding potential (LincRNA). We identified 207 novel differentially expressed LincRNAs in the testes (Supplementary Figure S14A). To explore the genomic context near differential LincRNAs we analyzed genes located up and downstream of LincRNAs. The genes located in the vicinity of differential LincRNAs are associated with transcriptional control and developmental regulation functions (Supplementary Figure S14B). We noted that highly differentially expressed LincRNAs are often located a long distance away from the transcription start sites (TSSs) of the genes. For example, we identified differentially expressed LincRNA located, respectively, at 430 and 269 kb away from and between the TSSs of the *Isl1* and *Parp8* genes (Supplementary Figure S14C). We acknowledge the possibility that LincRNAs does not necessarily regulate genes located in cis positions.

**Embryonic exposure to ATZ is associated with increases in the appearance of the new isoforms of RNA initiated from alternative TSSs, with increase in expression of alternatively spliced RNA isoforms and alternative polyadenylation (APA) transcripts**

**Figure 2.**
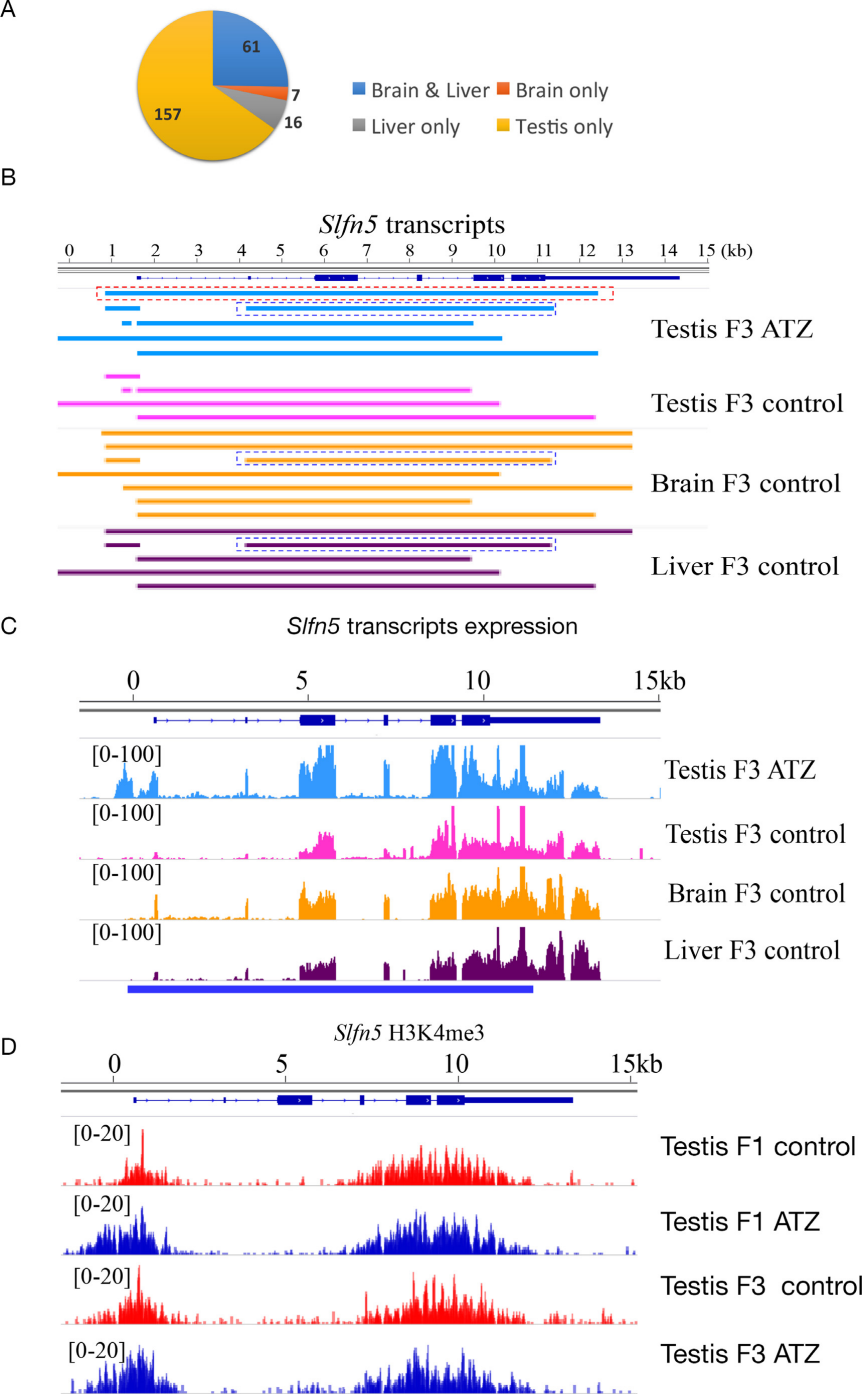
The alternative initiation of mRNA transcription affects the third generation after treatment. (**A**) The pie chart represents the transcripts with new TSSs in the testis, liver and brain tissues. (**B**) The schematic presentation of *Slfn5* transcripts. The blue dashed box shows a transcript that is expressing in ATZ-derived testis (FPKM = 1.13), this transcript is not expressing in the control testes but it is present in the control liver (FPKM < 0.01) and control brain (FPKM = 0.04); the red dashed box shows a new transcript, which is expressing in ATZ-derived testis (FPKM = 1.3), but is not expressing in the control testis, liver and brain. (**C**) Visualization of *Slfn5* expression in testis brain and liver illustrated by IGV. (**D**) The H3K4me3 peaks in the promoters of *Slfn5* genes in ATZ-derived males are wider in both F1 and F3 generation ATZ-derived males, two biological replicates were merged for simplicity. *P*-values in ChIP-seq were counted by comparison of normalized intensities at each peaks of two biological replicates from control and ATZ-derived samples with *Limma* statistical test. Benjamini–Hochberg (BH) correction was applied to the *P*-values and peaks with FDR less than 10% are considered statistically significantly different.

In addition to characterized transcripts, we identified 241 previously un-annotated differentially expressed transcripts in the testes of F3 males from ATZ-derived progeny. These new isoforms encode proteins with transcription factor activities and well-established developmental roles (Supplementary Table S5). Alternative transcription is often used for the tissue-specific production of new proteins. Therefore, we asked whether these novel testis transcripts are being expressed elsewhere in other tissues. We determined that out of 241 alternative new transcripts found in the testes, 61 are expressed in both the liver and the brain, 16 only in the liver and 7 only in the brain, while 157 new isoform transcripts are specific for the testes only (Figure 2A). For example, there are two differentially expressed transcripts of different size derived from the *Slfn5* gene in ATZ-derived F3 testes (Figure 2B) that are not present in control-derived testes. The longer transcript is also absent from the brain and liver tissues, while the shorter transcript is expressed (Figure 2B and C). The appearance of both transcripts in the testes hints at the important role of the *Slfn5* gene in ATZ-exposed males, as the *Slfn5* gene encodes a protein involved in cell cycle progression and the control of cellular proliferation (92). H3K4me3 peak at this gene changes its shape in F1 males and, to a lesser extent, in F3 ATZ-derived testes and becomes broader compared to the control (Figure 2D). At least in some cases, the alterations in the testicular chromatin structure prefigure similar changes in other tissues. For example, the liver-specific transcript *Adat1* (this gene's expression level in the testes is very low) changes the RNA expression level in liver only in F3 ATZ-derived males. We identified a change in the H3K4me3 occupancy at the *Adat1* promoter in the testes of F1 ATZ-derived males (Supplementary Figure S15), suggesting that the changes in the testes of F1 males could contribute to the alteration in expression of RNA in other, non-testis, tissues in F3 generation males.

Alternative splicing is another important tissue-specific mechanism for regulation of gene expression. We asked whether the alternative splicing mechanisms are also affected in the ATZ F3 progeny of males. We found transcripts with alternative TSSs that also have fewer exons or more exons, as well as transcripts with the same number of exons but using different donor–acceptor splicing sites (Supplementary Table S5).

In addition to alternative TSS isoforms and alternatively spliced transcripts, we identified 50 differentially expressed transcripts with APA sites (Supplementary Table S6). Among differentially expressed APA transcripts, 21 are found in the testes, 18 in the liver and 11 in the brain. Recently, it has been shown that APA regulation is important for RNA processing during spermatogenesis (93). We compared APA transcripts with published datasets derived from different testis cell types (87) and found that the *Cd68* APA transcript has altered 3′UTR. The change of 3′UTR in *Cd68* APA transcript occurs during spermatogenesis. The *Cd68* APA transcript in F3 ATZ-derived testes has an altered APA site, which in the control testes is detected only in a small fraction of leptotene spermatocytes and at later stages it becomes shorter. However, in F3 ATZ-derived testes, the transcript does not change in length (Supplementary Figure S16). These data suggest that *Cd68* transcript acquires the altered APA site specifically in the ATZ lineage, however, the role of this transcript or *Cd68* gene in meiosis is unknown.

In summary, ATZ exposure during embryonic development in F1 generation associated with the appearance of transcripts with novel transcriptional initiation sites, differentially spliced and APA transcripts in the third generation after treatment.

### Analysis of H3K4 trimethylation reveals the global decrease of these marks in ATZ-derived testes in the third generation

**Figure 3.**
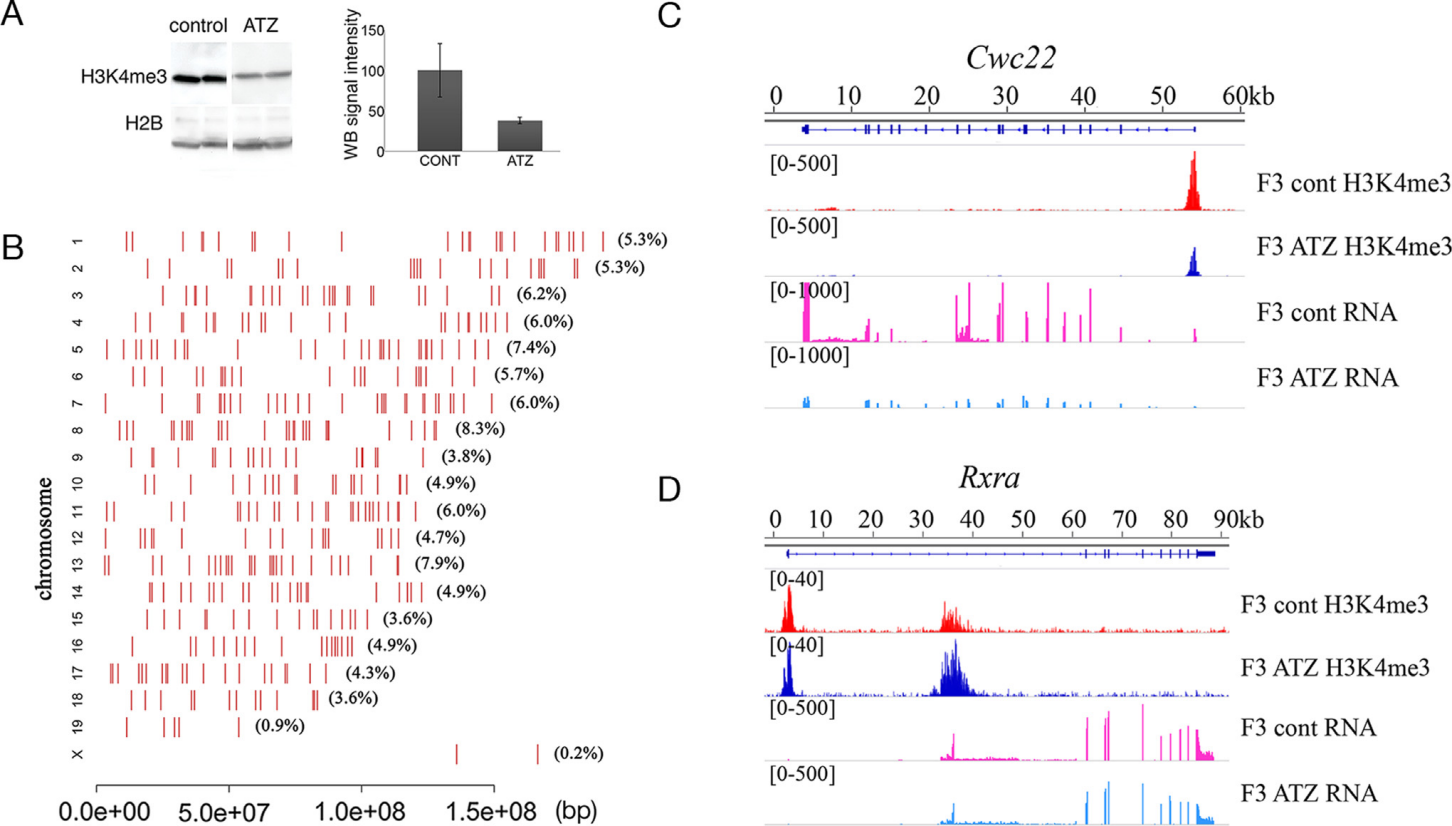
Histone H3K4me3 occupancy is globally affected in the third generation after treatment. (**A**) The decrease in histone H3K4me3 protein levels in treated mice. Histones were purified from F3 ATZ-derived and control male testes and equal amounts of proteins were analyzed by Western blot as described in the ‘Materials and Methods’ section. The control and ATZ samples were run in the same gel. (**B**) The altered genome-wide histone H3K4me3 peak occupancy in F3 ATZ-derived males. The red bars represent the position on the chromosome and the proportion of altered peaks per chromosome is indicated in brackets. (**C**) The occupancy of H3K4me3 marks (FC = 0.33) and transcription level (FC = 0.08) for the *Cwc22* gene were both decreased in the third-generation males after exposure, (**D**) Despite the increased H3K4me3 occupancy (FC = 3) of ATZ-derived mice, the RNA transcript level of *Rxra* is not altered in ATZ F3 males. Histone H3K4me3 peaks were identified as described in the ‘Materials and Methods’ section and are expressed as a normalized tag number at each position. The RNA profile is shown as vertical bars (exons), and the values at each position were calculated as described in the ‘Materials and Methods’ section. H3K4me3 and RNA profiles were visualized using IGV genome viewer version 2.3.36.

To determine whether embryonic exposure to ATZ specifically affects H3K4me3 distribution, we examined the H3K4me3 level from the purified histone protein fraction in the testes of F3 generation males. We found that the total protein level of H3K4me3 is decreased in F3 ATZ-lineage males (Figure 3A). To investigate the genome-wide profile of H3K4me3 marks, we performed ChIP-seq using anti-H3K4me3 antibody. We identified ∼48 thousand peaks (*P*-value of 10^−5^, Poisson distribution test, generated by MACS 2.0.1) (Supplementary Figure S17A and B). We found the differential peaks to be distributed throughout the genome (Figure 3B). A total of 473 peaks show altered occupancy in the ATZ-lineage testes (FC > 1.5 and FDR < 10%): ∼9/10 (424) peaks show decreased H3K4me3 occupancy in the ATZ-lineage testis, while only 49 are increased (FC > 1.5 and FDR < 10%). Some of the altered peaks were randomly selected and confirmed by ChIP-qPCR (Supplementary Figure S18). Not all of the promoters with altered H3K4me3 occupancy correlate with changes in RNA transcription. For example, for the *Cwc22* gene, there are changes in both H3K4me3 occupancy and RNA expression level (Figure 3C). On the contrary, the *Rxra* promoter (Figure 3D) has increased occupancy of H3K4me3, but its transcription level is unchanged. To reveal whether the decrease in H3K4me3 methylation marks is associated with the altered expression level of histone modifier enzymes, we analyzed the expression patterns of histone 3 lysine 4 methyltransferases and demethylases by inspecting RNA-seq data. We found the expression level of histone H3 lysine 4 methyltransferase mRNA (*Whsc1l1*) to be significantly decreased. In contrast, the expression levels of histone H3 lysine demethylase, *Kdm5b* is increased in the testes of F3 ATZ-lineage males (Supplementary Figure S19). These data suggest that the altered activities of histone modifying enzymes in the F3 generation may contribute to the global changes in H3K4me3 mark occupancy. To investigate the genomic context of regions with altered H3K4me3 occupancy, we inspected the genes located in the vicinity of differential peaks using GREAT, v3.0.0 (66). We found changes in H3K4me3 occupancy at 722 genes in F3 ATZ-lineage males, which are specifically enriched in gene clusters corresponding to several cellular functions (Table 1). Specifically, genes associated with the regulation of metabolic processes, transcriptional regulation and mitosis are among the most affected (the full list of gene clusters is presented in Supplementary Table S7). The promoters of *Ezh2, Tgif1* and *Zfp536* genes that negatively regulate the retinoic acid (RA) signaling pathway, show decreased H3K4me3 occupancy; in contrast, the region of *Rxra* have increased H3K4me3 occupancy. RA plays key roles in SG differentiation and is essential for meiosis initiation (94). Our data suggests that changed H3K4me3 marks within genes regulating RA pathway may play a role in impaired spermatogenesis.

**Table 1.** Functional annotation of altered H3K4me3 peaks in F3

GO	Gene ontology term description	*P*-value	No. of genes
GO:0031323	Regulation of cellular metabolic process	2.42E-04	188
GO:0048387	Negative regulation of retinoic acid receptor signaling pathway	7.31E-04	3
GO:0032318	Regulation of Ras GTPase activity	9.29E-04	16
GO:0010468	Regulation of gene expression	9.91E-04	147
GO:0016070	RNA metabolic process	6,81E-03	105
GO:0031124	mRNA 3′-end processing	4.88E-03	5
GO:0032204	Regulation of telomere maintenance	5.31E-03	3
GO:0007067	Mitosis	8.67E-03	18
GO:0051301	Cell division	1.36E-02	23
GO:0033044	Regulation of chromosome organization	4.69E-02	8

To assess the origin of differential H3K4me3 peaks, we separated the testicular cells into meiotic cells (spermatocytes) and post-meiotic cells (spermatids) using cell sedimentation in bovine serum albumin gradient method as described in SI ‘Methods’ section and performed ChIP-seq using H3K4me3 from recovered fractions. We identified 53 974 and 52 201 peaks in spermatocyte and spermatid fractions, respectively. We compared the differential peaks with normalized peak intensity of each fraction. We identified 273 differential peaks that have high signal in spermatocytes (meiotic fraction) and 200 differential peaks with high signal in spermatid fractions (postmeiotic fraction). H3K4me3 peaks in both spermatocytes and spermatids are generally located distally from promoters (Supplementary Figures S20A and S21A).

The identified peaks were functionally annotated with GREAT. We found that differential peaks that belong to spermatocyte fraction are enriched in epithelial cell development genes and meiosis-specific genes, including *Mei1, Trip13, Spin1, Spdya* and *Wrn* (Supplementary Figure S20B). Differential peaks of spermatid fraction are enriched in genes associated with mitosis and chromatin organization functions, including*Akap9, Asz1, Cdc7, Cdk1, Pabpc1l, Rad21, Trip13* and *Vrk1* (Supplementary Figure S21B).

To identify peaks of SG fractions we compared the differential peaks with published dataset (95). The differential regions of SG fraction are enriched in cell cycle and RNA processing genes. We also found that SG fraction of differential peaks is enriched in genes that encode proteins with histone modifying activities, such EZH2 (H3K27me3 methyltransferase), PHF2 *(*H3K9me demethylase) and BRD4 (chromatin remodeling factor). These enzymes maintain the pluripotent state of the cell (Supplementary Figure S22B). They are also required for mesenchymal to epithelial transition during induced reprogramming of human fibroblasts into stem-like cells (96,97). BRD4 is an important factor of self-renewal ability and pluripotency in ESCs (98). The reduced H3K4me3 peaks at genes encoding these important factors could be established during germline reprogramming, as these factors are required earlier in development. Abnormal H3K4me3 distribution can affect the establishment of other histone marks at meiosis. To investigate whether the differential peaks specifically result from abnormal histone H4 acetylation during the chromatin remodeling events of spermiogenesis we compared the differentially peaks with a recently published dataset of H4K5ac and H4K8ac of ChIP-seq data in spermatocytes and round spermatids (80). We found that almost all differential peaks correlates with H4K5ac or H4K8ac peaks in both fractions and are shared between spermatocyte and spermatid fractions (Supplementary Figure S23). The reduction of histone H4K5ac acetylation suggests that other histone modifications are also affected and could contribute to abnormal spermatogenesis. Overall, our data suggests that the exposure to ATZ in embryonic stage is associated with the global decrease of H3K4me3 occupancy in the third generation males.

### Integrative analysis of ChIP-seq and RNA-seq data

Our results demonstrate that the changes in H3K4me3 occupancy do not, by themselves, account for the differences in transcriptional output at adult developmental stage. As H3K4me3 marks are found both at inactive promoters (99,100) and in developmentally poised genes (101), we suggest that some promoters with altered histone H3K4me3 marks are likely to be in the ‘inactive’ state in adult mice; the initiation of transcription from promoters ‘inactive’ in later life occurs at early developmental stage, but changes in histone occupancy are retained in adult.

To reveal the association between gene expression and histone H3K4me3 occupancy data, we performed integrative analysis of ChIP-seq and RNA-seq F3 generation data. Our data show that out of 725 differentially expressed transcripts in testis, 259 (36%) harbor altered H3K4me3 marks (FC > 1.2) within 5 kb of the TSS. Because H3K4me3 marks are also known to associate with meiotic double strand break (DSB) hotspots, we excluded H3K4me3 peaks that could be DSB-associated, namely the intergenic distally located weak signal peaks. A previous work determined that peaks associated with DSBs normally comprise ∼17% of the total number of H3K4me3 peaks (56).

This current work suggests that, in addition to alterations in the H3K4me3 occupancy in proximal locations, a large number of testis differential peaks in the ATZ lineage are located distally and correspond to the enhancers and promoters of non-coding RNAs. We identified 121 out of 473 differential peaks (25.6%) as presumptive promoters; the majority of peaks correspond to presumptive enhancers (74.4%). To correlate the promoter peaks intensity with altered RNA transcript expression level, we analysis the altered promoter peaks corresponding to alterations in RNA expression levels and found 56 out of 121 promoter peaks correlates with altered RNA expression (FC > 1.2) (Supplementary Figure S24). However, we found that only a few regions gained or lost peaks in ATZ males (Supplementary Figure S25), with most of them located in intergenic regions.

We also analyzed the enhancer-associated peaks using Genomic Regions Enrichment of Annotations Tool (GREAT). We found that 71 enhancer-associated genes (out of 558) are genes encoding for transcription factors. These are genes essential for embryogenesis, such as *Dmrt1, Cux1, Runx1, Zfpm2, Foxc2, Foxk1, Sox6, Sox14, Sp5, Asxl2* among others. For example, *Dmrt1* is an essential gene regulating pluripotency during development (102). This data suggests that changes in H3K4me3 occupancy at the enhancer regions regulating developmental genes may contribute to alteration of developmental gene expression. We found that 22 testis DEGs out of 637 have altered peaks in enhancers, including *Rfx8, Zfpm2, Sox6 and Klf2*.

In summary, while a small fraction of DEGs harbor altered H3K4me3 pattern at their TSS, the majority of modified H3K4me3 peaks are localized to enhancers.

### Comparison of F1 and F3 ChIP-seq H3K4me3 occupancy reveals common modified regions

**Figure 4.**
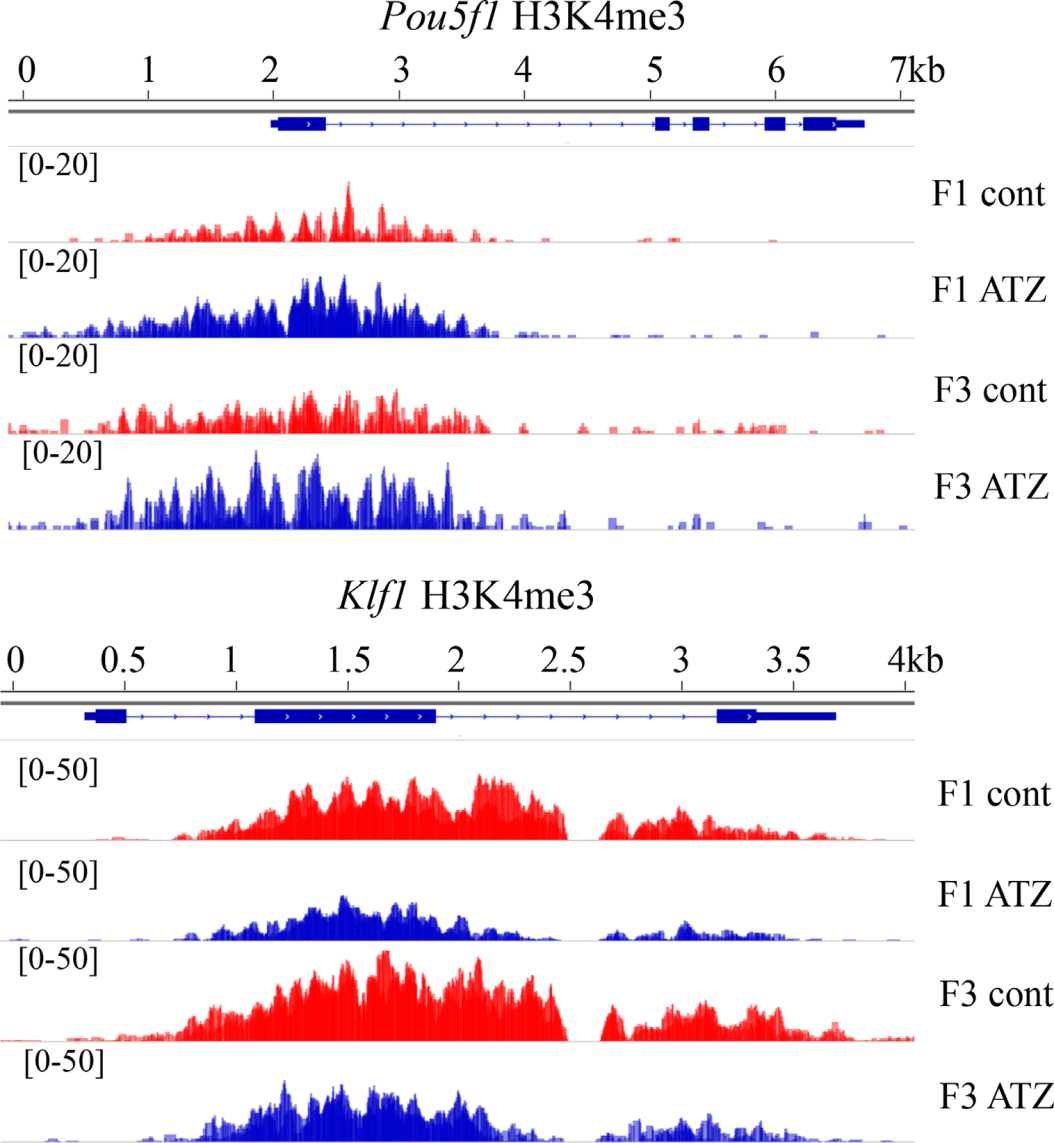
Histone H3K4me3 occupancy is affected in the *Pou5f1* and *Klf1* genes in the first generation after treatment and remains detectable in the third generation. The schematic presentation of H3K4me3 marks in the (**A**) *Pouf5f1* and (**B**) *Klf1* gene. These genes have altered H3K4me3 peaks in the F1 generation and the changes are also detectable in the F3 generation. In the *Pouf5f1* gene, the H3K4me3 occupancy is increased in F1 and the change is still present in F3. In the *Klf1* gene, the H3K4me3 occupancy is decreased in F1 and the change is still present in F3.

To understand the pattern of H3K4me3 occupancy in the F3 generation of ATZ-derived males, it was essential to first characterize the chromatin landscape in earlier generations and relate it to F3. To this end, we performed ChIP-seq on the F1 generation of ATZ and control males using antibody against H3K4me3. We identified ∼48 000 peaks using a *P*-value of 10^−5^. Using a cut-off fold change value of 2 and FDR < 10%, we identified 270 differential peaks between control and F1 ATZ-lineage males. To reveal the regions in the genome that become altered in F1 in ATZ-derived males and could contribute to transgenerational change in the F3 generation males, we performed functional annotation using GREAT v3.0.0. We identified the promoters of 375 genes with altered H3K4me3 occupancy. We found the highest enrichment of altered H3K4me3 occupancy at genes associated with stem cell differentiation and stem cell development and maintenance (Table 2). The full list of genes for the enriched clusters is presented in Supplementary Table S8. Remarkably, in F1 males H3K4me3 peaks show increased occupancy in F1 males proximal to genes encoding key pluripotency factors such as *Sox2, Pou5f1* and *Nanos2* and transcription factors such as *Sox9* and decreased occupancy at genes encoding factors of stem cell differentiation factors such as *Cyp26a1, Eomes, Hey1, Isl1, Jarid2, Med10, Msi2* and *Nog*. To examine whether F3 generation differential peaks are similar to those in the F1 generation, we compared the two datasets. We found that 200 out of 473 regions with altered H3K4me3 occupancy in F3 were also detected in F1 males. These regions are located in the promoters of genes associated with regulation of cell differentiation, including promoters of *Pou5f1, Sox6, Klf1, Klf2, Foxh1, Jmjd1c, Nfatc2ip, Rxra* and *Asxl2*. Among these genes, the *Pouf5f1* play especially prominent roles in a ground state pluripotency (103). While the mechanism of epigenetic inheritance remains to be understood, this data supports the key notion that epigenetic state of key pluripotency genes is preserved in subsequent generations. Specifically, *Klf1* and *Pou5f1* have altered H3K4me3 marks at their TSSs in the testes of F1 ATZ-derived males compared to controls. In F3 ATZ-derived males, these H3K4me3 marks remain altered, albeit to a lesser extent (Figure 4). In summary, our data suggests that altered H3K4me3 peaks are found at the promoters of key pluripotency-associated genes in F1 generation of ATZ-derived males and these changes are retained in F3 generation.

**Table 2.** Functional annotation of altered H3K4me3 peaks in F1

GO term	Gene ontology term description	*P-*value	No. of genes
GO:0048863	Stem cell differentiation	3.74E-05	15
GO:0019827	Stem cell maintenance	5,27E-04	8
GO:0033993	Response to lipid	5.31E-04	21
GO:0043583	Ear development	1.03E-03	11
GO:0050870	Positive regulation of T cell activation	1.42E-03	8
GO:0032526	Response to retinoic acid	3.15E-03	6
GO:0010033	Response to organic substance	4.02E-03	43
GO:0060008	Sertoli cell differentiation	5.02E-03	3
GO:0002064	Epithelial cell development	9.94E-03	10
GO:0006282	Regulation of DNA repair	1.10E-02	4

### Differential peaks are enriched in *Wt1* and *Sp1/Sp3and Sp4* motifs

**Figure 5.**
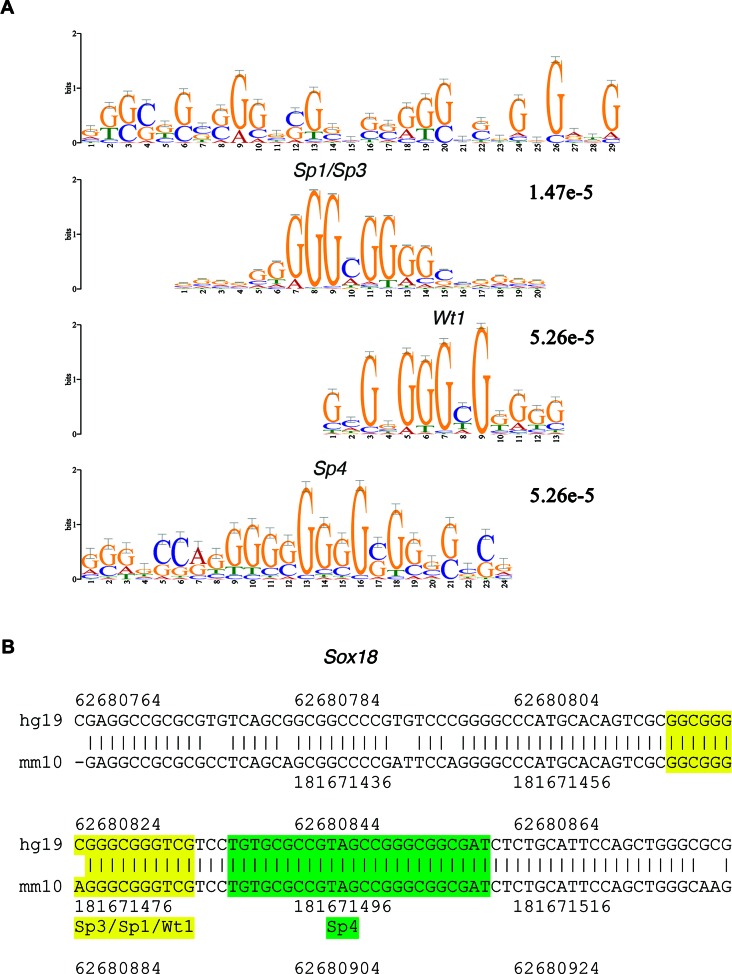
The altered H3K4me3 occupancy regions are enriched in the 29-mer motif. (**A**) The 29-mer motif enriched in altered peaks. SP1, WT1 and SP3 binding sites are shown below the 29-mer motif. The 500-bp summits of differential peaks were used for analysis as described in the ‘Materials and Methods’ section. (**B**) The presentation of conservative binding sites located within the *Sox18* gene; the conserved region containing binding sites for SP1/SP3/ WT1 is highlighted in yellow and the SP4 binding site is shown in green.

To explore the role for transcriptional control of epigenetic modifications in F3 progeny of ATZ-derived mice we analyzed altered F3 H3K4me3 peaks for motif enrichment using MEME-ChIP. We noted a highly enriched 29-mer motif (motif 1) located within the sequences of differential peaks (Figure 5A). The inspection of this motif using TomTom (68) revealed parts of the motif that resemble the binding sites for the Specifity protein (SP) group (SP1, SP3, SP4) and Wilms tumor 1 (WT1) transcriptional factors. The binding sites for these factors include a GC box and have a high GC content. Importantly, the analysis of the 500 bp upstream sequences corresponding to the promoters of genes encoding testis differentially expressed alternative transcripts also showed the presence of a highly similar motif identified in H3K4me3 altered peaks (Supplementary Figure S26). These data support the hypothesis that these transcription factors may contribute to establishing the H3K4me3 occupancy of some promoters, including the promoters of alternative transcription initiation. To further explore the involvement of these factors in gene network regulation, we identified genes located near the binding sites of motif 1. We found the motif's binding sites with FIMO and estimated the conservation score using UCSC conservation estimation tools. The sites with more than 70% conservation between mammalian species were retained (Supplementary Tables S9 and 10). We found the motif presents in many genes associated with transcription factor activity (*Zhx2, Zfp36l1, Gabrb3, Etv6, Sox18, Zfat, Cux1*) (Supplementary Tables S9 and 10). For example, two conservative blocks are present within the promoter of *Sox18*, which contain binding sites for the SP1/SP3/WT1 and SP4 factors (Figure 5B). The analysis of the strongest sperm H3K4me3 peaks identified the presence of a similar 29-mer motif (Supplementary Figure S26). In summary, our data shows that regions of altered H3K4me3 occupancy are enriched in SP family and WT1 transcription factor binding sites.

### ATZ effects on germline during embryonic development

To assess whether embryonic ATZ exposure directly affects the germ line cells, we performed the analysis of the number of germ cells using the paraffin testis sections at E15.5 and E18.5 of F1 generation males immunostained with the antibody against specific germ cell marker, DDX4 (Supplementary Figure S27A). The quantitation of DDX4-positive cells revealed that germline population is significantly diminished at E18.5 (Supplementary Figure S27B and C). The somatic to germ line reprogramming is also associated with global DNA demethylation and remethylation. We analyzed the expression level of *de novo* DNA methyltransferases; the significant increase of *Dnmt3a* expression was found (Supplementary Figure S27D). Changes in DNA methylation in embryonic germ cells can be associated with changes in histone epigenetic landscape since DNA methylation and histone modifications are functionally linked and can affect gene expression (104). It is also known that the somatic-to-germ line reprogramming causes the activation of retrotransposon elements (105). To explore this possibility we analyzed by qPCR the activity of the most common retro-elements by qPCR. We found the significant increase in activity of retro-element *SINE-B1* in embryonic testis in F1 ATZ-lineage male (Supplementary Figure S27E); the expression of *SINE-B1* was also higher in F3 adult males (data not shown). Our data suggest that exposure to ATZ causes changes in embryonic germ cells, which could result in transcriptional network deregulation and transmit these changes to subsequent generations.

## DISCUSSION

### The transgenerational effects of ATZ on reproduction

This study was aimed at elucidating the transgenerational-inherited changes caused by embryonic exposure to ATZ. A study in a French cohort showed the association between the presence of low-dose ATZ metabolites in pregnant mothers and low birth weight in newborns (55). This study raises the possibility that exposure to ATZ can directly alter the fetal developmental program and that ATZ-induced effects can be inherited across generations. Here we used an outbred CD1 mouse model because outbred mice are more sensitive to toxic exposure than inbred mice (11) and are considered to be better predictors of the human response (106). Pregnant dams (F0) carrying F1 offspring were exposed to the herbicide ATZ between embryonic (E) days E6.5 to E15.5. The F1 generation was bred to obtain F2, and the progeny of F2 were bred to obtain the F3 generation. The F3 generation adult males (never directly exposed to ATZ) were studied for changes in the somatic and germline tissues. The majority of our experiments were focused on the testis, due to its role in the production of gametes and as a major tissue where the transgenerational effects will be generated (107). We detected meiotic defects and showed that telomeres are often fused during meiosis in ATZ-lineage. The telomeres play a special role during meiosis, as they are required for initiation of homologous recombination. Telomere-mediated movement along the nuclear envelope is crucial for homologous pairing and synapsis during meiosis (108). Meiotic telomeres carry a set of specific proteins such as SUN1, SUN2 and TERB1, which associate with telomeres between the leptotene and diplotene stages during meiotic prophase I (109,110). The telomere fusion during meiosis in our study could be associated with altered function of these telomere-associated proteins. Specifically, we found *Sun1* gene to be differentially expressed and observed an increase in TERF1 at fused telomeres. This is important as the recent paper showed that the increase in TERF1 leads to formation of chromosome fusions (111). Altogether our data support the notion that meiosis is transgenerationally affected. We also found the decrease in protamine and in the H4K5ac levels. Histone-to-protamine replacement is a dynamic process that depends on efficiency of BRDT binding to acetylated histones H4 (H4K5Ac and H4K8AC). BRDT binding is competing with histone butyrylation, which is associated with transcriptional activity (80). We suggest that transcriptional deregulation in SG is responsible for changes in all testis cell types, because meiotic and post-meiotic cells are derived from SG. Indeed, SG derived from ATZ-lineage Primordial Germ Cells (PGCs) have altered transcriptional network. We propose that direct effects of ATZ on mitochondrial function are the most likely cause for these changes in germ cells. The ATZ-responsive DEGs play an important role in many cellular processes, including cell signaling, chromatin remodeling and cell cycle. ATZ was originally introduced to limit the growth of broadleaf plants. It has been proposed that ATZ induces oxidative damage in plants caused by the breakdown in the electron transport chain process during photosynthesis (112). In eukaryotes, an important electron transport chain and ATP synthase function are found in inner mitochondrial membrane. Our data suggest that the mechanism where mitochondrial function and oxidative phosphorylation are primary targets of ATZ exposure. ATZ-induced downregulation of mitochondrial transport could impair the pathways in developing germ cells that are dependent on ample ATP energy. The dramatic reduction of *Atpaf2* expression and deregulation of a large number of genes encoding ATP-binding proteins suggest that changes induced in embryonic germ cells are likely preserved in SG. Importantly, ATP synthase is a key protein required for germ cell differentiation (113) and expression changes in genes, encoding the subunits or assembly proteins of ATP synthase, such as *Atpaf2*, could contribute to germ cell development. We also explored the idea that at least some changes in SG-derived lineages could originate in the cells that express POU5F1 as a key pluripotency factor. To evaluate this hypothesis, we asked whether DEGs we identified are targets of POU5F1. We performed the comparative analysis of DEGs and *Pou5f1* targets using BioGRID (BioGRID Version 3.4.139) (114) and published dataset (115). We identified 18 *Pou5f1* target genes that are altered in ATZ lineage SG (Supplementary Figure S12B). These are key regulators of germ cell development *(Esrrb, Gatad2b, Ncor1)* and genes that are important for spermatogenesis *(Rif1, Ssrp1)* and cell cycle (*Rfc1, Rfc5)*. RFC1 and RFC2 are also ATP-binding proteins. The SG derived from affected cells have modified transcriptional network that impacts both meiotic and post-meiotic testis fractions. The defective meiotic and post-meiotic cells could be eliminated either by Sertoli cell phagocytosis (116) or in epididymis, as mouse epididymis is able to phagocyte immature germ cells (117) and ultimately lead to spermatozoa decrease. Besides that, transcriptional factors expressed in the epididymis control the chromatin assembly on the genome-wide level and could contribute to transmission of altered information to subsequent progeny. For example, the members of RHOX family proteins (118) and SHH (119) are essential for sperm maturation and mobility. The forkhead transcription factors, (e.g. FOXA2) play an important role in steroid hormone-responsive gene activation (119) and FOXI1 is required for male fertility (120). ATZ-induced chromatin changes could interfere with the ability of these transcription factors to regulate gene expression in germ cells.

### The effects of ATZ on tissue-specific alternative transcription

We now report the genome-wide transgenerational effects on tissue-specific alternative transcription in the testes. Alternative transcription initiation leads to the formation of transcripts with different first exon or different 5′-UTR. It has been shown that TSS selection has a critical role during development, cell differentiation and could be associated with cancer and diseases (reviewed in (121)). Genetic or epigenetic changes in the promoters as well as changes in distal elements (enhancers) could lead to a disease (121). Alternative promoters are regulated through the action of DNA methylation and histone modifications. For example, *Dclk1* expression in the brain is initiated from an upstream promoter during early postnatal development, and is switched to the downstream promoter in adult tissue (122). This promoter-switching was associated with changes in the H3K4 and H3K27 trimethylation status (122). We suggest that the appearance of a large number of novel transcripts is likely due to alteration of epigenetic states at many promoters which occurred during exposure or subsequent reprogramming events.

### The role of SP family and WT1 transcription factors in transgenerational inheritance

The presence of SP family binding sites in a large number of promoters with altered TSS transcripts and in many altered H3K4me3 peaks suggests their potential involvement in the epigenetic regulation of at least some of these promoters. Transcription factors of SP1 family and WT1 are implicated in regulating the expression of genes involved in cellular differentiation and embryonic development (123). Moreover, these proteins mediate the RA pathway (124) and are important for murine *Nanog* gene expression (125). WT1 factor is prominently expressed in germ cells during embryonic development and plays essential role in gametogenesis. It specifically activates the transcriptional expression of *Nr5a1* and thus promotes gonadogenesis (126). We suggest that epigenetic changes in many genes are mediated via a combined action of SP family and WT1 factors, which are expressing during development and could deregulated upon ATZ exposure.

### Global effects on H3K4me3 histone marks in F3 generation

In this work, we identified an increase in *Pou5f1* H3K4me3 peaks in the ATZ lineage. We suggest that this change first appears during development as a result of ATZ exposure and impacts *Pou5f1* regulation. The *Pou5f1* promoter contains response elements for SP1 protein (127), and we provide evidence that large number of altered peaks contain SP1-binding site. Another plausible explanation for this change could be regulation of *Pou5f1* expression by other transcriptional factors, e.g. NR5A2. It has been shown that NR5A2 binds directly to both promoter and enhancer regions in *Pou5f1* and activates its expression (128). *Nr5a2* is highly expressed during development and the knockdown of *Nr5a2* in zygote leads to downregulation of *Pou5f1* expression (24). In our previous work we showed that mice exposed to ATZ have a higher number of H3K4me3 peaks that contain *Nr5a2* motif (42). It has also been shown before that ATZ activates NR5A receptors (35).

We report that a subset of H3K4me3 peaks altered in F1 ATZ-derived males is also altered in F3 ATZ-derived males. Importantly, the regions where the altered H3K4me3 pattern between F1 and F3 is conserved include the promoters of genes associated with maintenance of pluripotent state and germ cell differentiation. We suggest that these regions are altered during reprogramming in ATZ exposed males. As we discussed in the ‘Introduction’ section, there exists some disagreement in the mapping of sperm nucleosome-retaining regions. In the first human dataset (17) the retention prevalence of developmental promoters over the gene poor regions was suggested. Later, a study that utilized fixed chromatin and a smaller amount of micrococcal nuclease concluded that there is a prevalence of the gene-poor regions in the nucleosome fractions (19,20). A careful inspection of the latter datasets from human and bovine sperm nucleosome retaining regions still shows a limited number of promoters present corresponding to genes of signal transduction, protein processing and ATP-binding, suggesting their role in the early zygote development (20). Furthermore, the overlap between human and bovine data showed the significant enrichment in genes associated with signal transduction and RNA and protein processing factors and suggests the conservative mechanism of nucleosome retention (20). The comparison of our murine data with human data (which contain regions not enriched in developmental genes) showed that 47% of genes located near murine differential peaks are also retained in human sperm, including genes such as *Phf2, Ezh2, Zhx2 and Sox6* among others (Supplementary Table S11). However, whether the differences we observed are sufficient to impact the zygote development remains to be understood. Our model predicts that the changes in germ cells induced by ATZ will be preserved at limited genomic regions. Specifically, we propose that the genes, which are expressed from both paternal and maternal alleles in the very early zygote (such as *Pou5f1, Nr5a2, Klf2*) are required to be in an open chromatin state (24) that likely preserves their histones as well as the histone modifications. Other potential candidates to mediate ATZ effects are ATP-binding proteins, since human sperm nucleosome fraction of genome is enriched in genes encoding them (20). The genes for proteins with chromatin modifications function, which have open chromatin state at distally located promoters and are essential for establishment of early pluripotent state (24) are also among the most plausible candidates.

We acknowledge the possibility that these are not the only factors that mediate ATZ effect. Other histone post-translational modifications and non-coding RNAs could be involved in transgenerational transmission of the paternal epigenetic program. At least 24 post-translational histone modifications were identified in sperm, which includes 4 unique to sperm (129), suggesting a possible role of these marks in mediating the flow of information between generations. Histone H4 acetylation, which plays important role in spermatogenesis and early zygote, is the most plausible candidate. Other histone modifications (e.g. ubiquitination) also occur simultaneously with histone acetylation and may play roles in nucleosome destabilization for facilitating histone-to-protamine transition (130). In summary, histone epigenetic landscape can be transmitted to subsequent generation via gametes and can be targeted by environmental factors.

### The comparison of ATZ F3 and vinclozolin-induced transgenerational inheritance data reveals the common region of effects

To determine whether exposure to different toxicants targets different or similar genomic regions, we compared the H3K4me3 regions affected by ATZ and the previously published data on differentially methylated regions in a vinclozolin study (12). In that study, the authors analyzed the transgenerational effects of vinclozolin on F3 male mice. The sperm of F3 males was analyzed by methylated DNA immunoprecipitation (MEDIP) to identify the differentially methylated regions, revealing 52 regions of altered methylation. We compared that data and the data obtained in this study. We found 13 regions altered in F3 vinclozolin-derived males that were also affected in F3 ATZ-derived males (Supplementary Table S12), including promoters of splicing genes (*Cwc22, Arl6ip4*), cell growth- and differentiation- (*Etv1, Bop1, Sepw1*) and cell adhesion- (*Itgb3*) associated genes. Combined, these data hint at the existence of ‘sensitive’ regions in the mouse genome that are involved in transgenerational inheritance, albeit mediated through different mechanisms. We suggest that these regions possess vulnerable chromatin structure, which makes them sensitive to exposure. Additionally, the modified epigenetic state could reflect the involvement of these regions in responding to toxic conditions, for example, to augment protein synthesis, which requires a new chromatin state. Both DNA methylation and histone modifications must cooperate in generating a defined epigenetic environment to respond to toxic stress. A functional connection between histone H3 methylation and DNA methylation state is known to exist, and histone H3K4me3 marks instruct DNA methylation by inhibiting the binding of *de novo* methylases (131,132). We suggest that both of these mechanisms mediate the transgenerational inheritance and are functionally linked.

## CONCLUSION

Our data suggest that transgenerational inheritance in ATZ-lineage males is mediated via altered epigenetic state of defined regions in the genome.

## Supplementary Material

SUPPLEMENTARY DATA

## ACCESSION NUMBERS

All sequencing data from this study are publicly available and have been deposited in the National Center for Biotechnology Information Gene Expression Omnibus (GEO) with GSE number GSE81093.
