# Supplementary material for: Exposure to the widely used herbicide atrazine results in deregulation of global tissue-specific RNA transcription in the third generation and is associated with a global decrease of histone trimethylation in mice
Source: Nucleic Acids Res. 2016 Sep 20;44(20):9784–802. doi: 10.1093/nar/gkw840 (PMC5175363; doi:10.1093/nar/gkw840)
Supplement: SUPPLEMENTARY DATA [file supp_44_20_9784__index.html]

Exposure to the widely used herbicide atrazine results in deregulation of global tissue-specific RNA transcription in the third generation and is associated with a global decrease of histone trimethylation in mice — Exposure to the widely used herbicide atrazine results in deregulation of global tissue-specific RNA transcription in the third generation and is associated with a global decrease of histone trimethylation in mice — SUPPLEMENTARY DATA 

# Exposure to the widely used herbicide atrazine results in deregulation of global tissue-specific RNA transcription in the third generation and is associated with a global decrease of histone trimethylation in mice

## SUPPLEMENTARY DATA

- SUPPLEMENTARY DATA
