# Supplementary material for: Exposure to the widely used herbicide atrazine results in deregulation of global tissue-specific RNA transcription in the third generation and is associated with a global decrease of histone trimethylation in mice
Source: Nucleic Acids Res. 2016 Sep 20;44(20):9784–802. doi: 10.1093/nar/gkw840 (PMC5175363; doi:10.1093/nar/gkw840)
Supplement: SUPPLEMENTARY DATA [file supp_gkw840_nar-01413-v-2016-File007.pdf]

Supplementary Methods

Animals and atrazine exposure. All animal procedures were performed according to the guidelines for animal models in research defined by the Ethics Committee and approved by the Ministry of France (reference project number is 01861.02), and by persons who qualified for laboratory animal care and use. Eight-week-old adult CD1 mice were treated by oral gavage from E6.5 to E15.5 with atrazine in oil at a concentration of 100 mg/kg/day The control mice were treated with the same volume of oil. The 12 week old F1 male progeny were crossed with unrelated untreated 12-week-old female and progeny of F2 were crossed again to receive F3 male progeny. 12 week-old F1 and F3 ATZ-derived or control-derived male mice were euthanized, and reproductive organs, liver and hypothalamus were dissected. At least five independent experiments were performed with at least six-to-ten animals in each group.

Testosterone and FSH quantification. Serum was collected from ketamine/xylazine-anaesthetized adult animals by terminal cardiac exsanguination, and aliquots were stored at -20°C. Testosterone levels in the serum were assayed in duplicate using a commercial radioimmunoassay (RIA) based on competitive binding with I125-labeled testosterone (Immunotech, Beckman Coulter, Villepinte, France), according to the manufacturer's recommendations. FSH measurements were conducted according to a standard protocol provided by a FSH measurement kit (KA2330, Abnova, Walnut CA, USA). In each hormone measurement, the data from at least 10 treated and control animals were averaged and plotted, and the results were expressed in nanograms per milliliter.

Analysis of histology, apoptosis and germ cells/Sertoli cells numbers. For the histological analysis, testis samples were fixed in Bouin's solution and embedded in paraffin. Histological sections (5-µm thick) were stained with hematoxylin and eosin (H&E). The staging of cells in seminiferous tubules was performed according to an established method (Russell 1990), with analysis of at least 600 tubule sections. For immunohistochemistry (IHC), the animals were perfused, and the testes were fixed for 24 hours in 4% (wt/vol) paraformaldehyde (PFA), dehydrated and embedded in paraffin. For IHC, 5-µm-thick testis sections were incubated overnight at 4°C with goat anti-ZBTB16 (diluted at 1:500) and rat anti-GATA1 (diluted at 1:50). The sections were counterstained with 0.001% (vol/vol) 4,6-diamidino-2-phenylindole dihydrochloride (DAPI) and mounted in Vectashield (Vector Laboratories, UK).

Cells sorting by flow cytometry. Germ cells were isolated from control and ATZ-derived F3 male testes. The testes were subjected to collagenase/DNAse I treatment and purified by FACS, as described previously (Zhu, Shao et al. 2011). The dispersed cells were fixed in 1% (wt/vol) buffered PFA for 20 min at 4°C and permeabilized in a solution of 0.2% (wt/vol) saponin containing propidium iodide to label the DNA. Cells were sorted at concentration of 1X106 cells/ml on FACSCalibur cell-sorting machine. The data were analyzed by CellQuestPro software, which was included with the sorter.

Spermatozoa count. Spermatozoa counts were conducted according to an established protocol (Vallet-Erdtmann, Tavernier et al. 2004). Briefly, mice were euthanized, and each epididymis was dissected, rapidly frozen in liquid nitrogen and stored at -80°C until the sperm heads were counted as follows. The organ was first cut with a scalpel into several fragments and homogenized in 50 ml 0.15 M NaCl containing 0.005% (vol/vol) Triton X-100 (Sigma). After homogenization with three rounds of sonication (12 kHz), an aliquot of the cell suspension was loaded onto a Malassez hemocytometer, and spermatozoa heads were counted. The data from at least ten controls or F3 ATZ-derived animals were averaged and plotted, and presented as averaged sperm counts normalized to control; a t-test was performed using Excel software.

Antibodies. The following commercial antibodies were used: rat anti-GATA1 (sc-265) and goat anti-PRM2 (sc-23104) antibodies from Santa Cruz, rabbit anti-H3K4me3 (07-473), mouse anti-H2B (05-1352) and rabbit anti-H4K5Ac (07-327) from Millipore, rabbit anti-Ddx4 (ab13840) from Abcam, mouse anti-ACTB (A1978) from Sigma Aldrich and goat anti-PLZF (AF2944) from R&D systems. Secondary HRP antibodies were purchased from Jackson Laboratories. Fluorescent secondary Alexa antibodies were purchased from Invitrogen.

Histone purification and Western blot analysis.

Protein samples from F3 mouse testes were prepared using the EpiSeeker Histone Extraction Kit (Abcam, 113476) according to the manufacturer's supplied protocol. Briefly, mice testes were homogenized and centrifuged at 900 g for 5 min. The pellets were resuspended in lysis buffer and left on ice for 30 min. After centrifugation, the supernatant fractions containing acid-soluble proteins were transferred to new tubes, and the Balance-DTT buffer was added. The protein concentrations were determined using the Pierce™ 660nm Protein Assay (ThermoScientific, France). Five ug (H4K5ac), 10 ug (H2B) or 20 µg (H3K4me3) of protein were run on a 4-20% gradient gel (BioRad, USA) for 1 h. Proteins were transferred onto ImmobilonPSQ membranes (Millipore, France) using an electro-blotter system (TE77X; Hoefer, USA) and modified Towbin buffer (48 mM Tris base, 40 mM glycine and 0.1% (wt/vol) SDS) and methanol (20% (vol/vol) anode; 5% (vol/vol) cathode) for 2 hours. Proteins were detected using an H2B mouse monoclonal antibody, a PRM2 goat polyclonal antibody, an ACTB mouse monoclonal antibody and a H3K4me3 or a H4K5Ac rabbit polyclonal antibody. The antibodies were incubated in hybridization buffer. The primary antibodies were detected using secondary antibodies conjugated to horseradish peroxidase for 1 h. The signals were developed using the ECL-Plus Chemiluminescence kit (GE Healthcare, USA) and the ImageQuant 350 system (GE Healthcare, USA).

Meiotic surface spreads

The surface spreads were prepared in 4 independent experiments from F3 control or ATZ progeny as described in a previous study (Peters, Plug et al. 1997). The analysis of the meiotic substages was performed according to previous studies (Mahadevaiah, Turner et al. 2001, Page, de la Fuente et al. 2012). The images were taken from randomly chosen pachytene stages of prophase I of meiosis and reanalyzed independently by two researchers.

Fractionation of germ cells

Germ cells were separated according to (Bellve, 1979). Briefly, 8 C57Bl/6J twelve week-old male mice were perfused with PBS, and testes were dissected. Testes were digested with collagenase, trypsine and DNAse. The cells were loaded into 2-4% gradient of BSA and allowed to sediment. The spermatocytes and spermatids fractions were collected. The purity of the fractions was confirmed by DAPI staining of the small cell sample. Cells were fixed with PFA and used for ChIP-seq. The library and data analysis were done similar to analysis of total testis extracts.

Preparation and Immunohistochemistry of embryonic testis histological sections

To perform immunohistochemistry in E15.5 and E18.5 embryo, the testis from control or ATZ-treated groups were fixed in PFA 4% (v/v) solution for 16 hours, then were dehydrated and embedded in paraffin. Organs were cut and every 5th sections (5 µm-thick) were taken for a slide. In total, testes from at least 3 animals of each group were used. The sections were deparaffined and rehydrated and slides were placed in 0.01M solution of citrate buffer, pH6 at 80°C for 45min to unmask the epitopes. The sections were washed three times for five minutes in 1X PBS Tween 0.05% (v/v), then incubated with primary antibody anti-Ddx4 diluted PBS-Tween 0.05% (v/v) overnight at 4C ° in a humid chamber. This antibody recognizes all germ cells. The following day the sections were washed with PBS-Tween 0.05% and then incubated with florescent secondary antibody for 1 hour in a humidified chamber. The sections were all counterstained with 0.001 % (vol/vol) 4,6-diamidino-2-phenylindole dihydrochloride (DAPI) and mounted in Vectashield (Vector Laboratories, UK). The images were taken using an AxioImager microscope equipped with an AxioCam MRc5 camera and AxioVision software version 4.8.2 (Zeiss, Le Pecq, France) with a 20X objective lens. To quantify the number of Ddx4-positive cells, we take at least 10 pictures in at least 3 different slides per replicate. We counted the number of Ddx4-positive cells in each tubule present on the picture and we report this number to the surface of seminiferous tubule measure using ImageJ software.

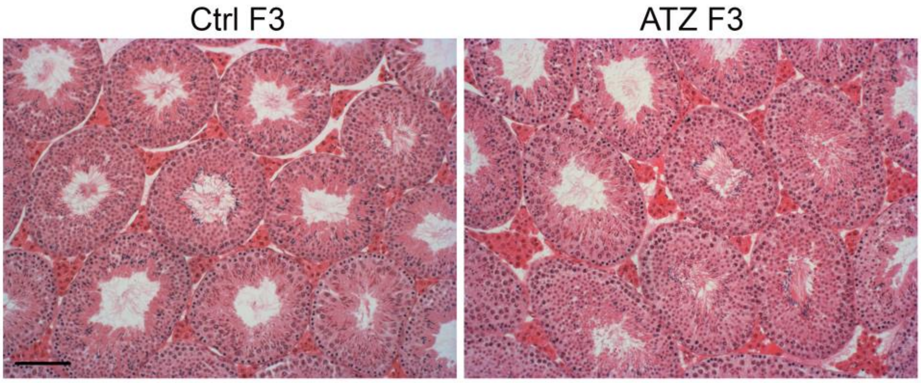

Supplementary_Figure_S1. Embryonic exposure to ATZ does not affect morphology in the third generation of males after treatment.

Representative image of H&E staining of the histological sections of F3 control (vehicle) and ATZ-derived mouse testes (12 weeks old). The sections from testis tissue were prepared as described in the Supplementary Methods section. We compared sections of seminiferous tubules at similar stages in at least 4 different samples of each group control (Ctrl F3) or ATZ (ATZ F3) derived, scale bar:150µM.

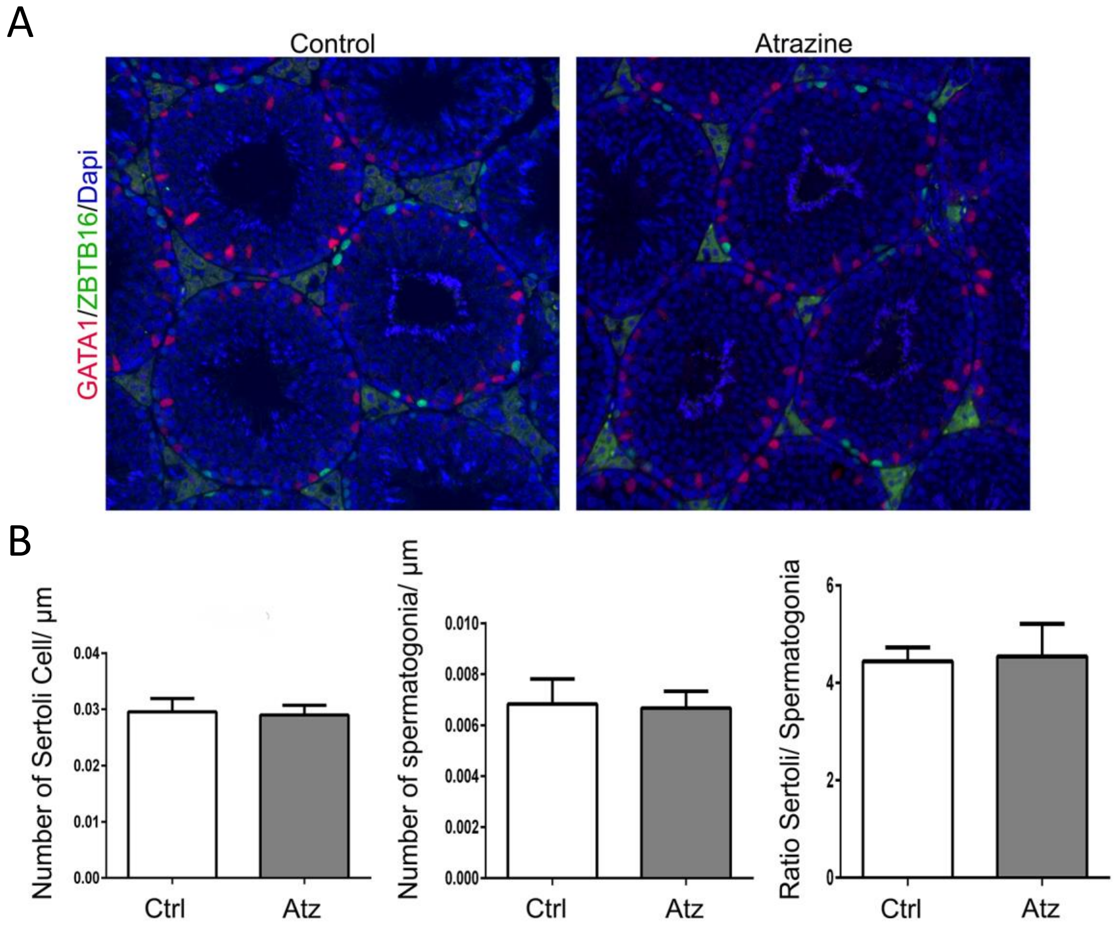

Supplementary_Figure_S2. Embryonic exposure to ATZ does not affect the number of undifferentiated spermatogonia and Sertoli cells in the F3 generations after treatment.

(A) Representative image of testis sections: Sertoli and spermatogonia cells are immunostained with anti– GATA1 or anti– ZBTB16 antibodies. (B) Quantitative analysis: cells, Sertoli cells and spermatogonia were counted manually at stage VII in F3 mouse testes. The contour of each tubule section was measured using the ImageJ software. The values indicate the cell counts per micrometer of tubule circumference. Ratio of the number of Sertoli cells per spermatogonia is also indicated. n=4, Scale bar: 50 µm. The immunostaining of testis sections was performed as described in the Supplementary Methods section.

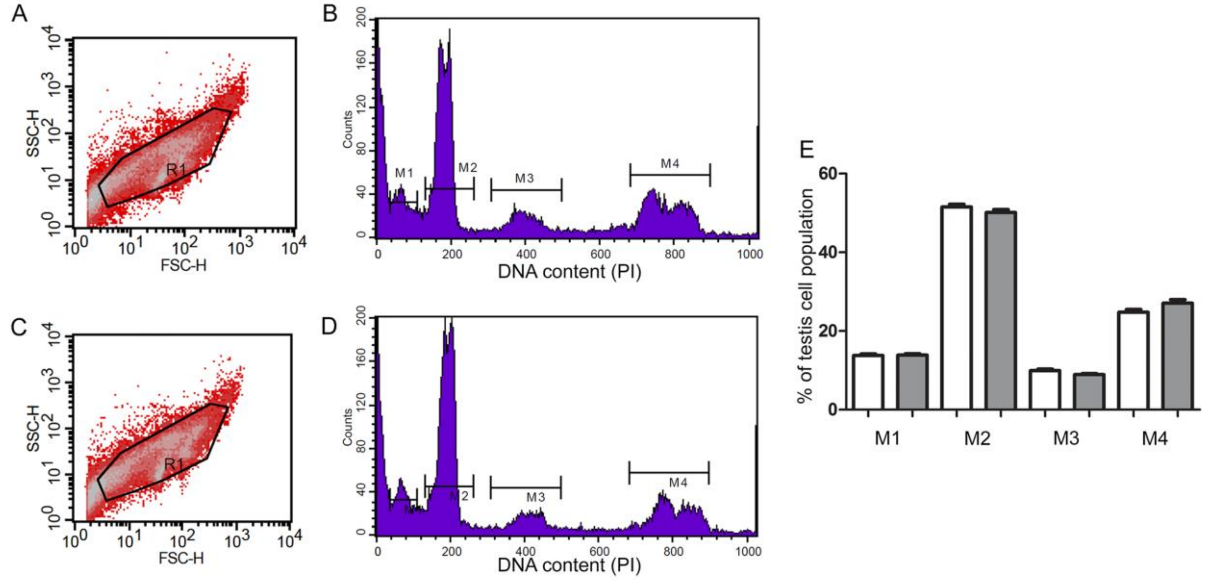

Supplementary_Figure_S3. Embryonic exposure to ATZ does not affect cell populations in seminiferous tubules in the F3 generation of males after treatment.

(A-D) Representative image of a flow cytometric analysis of germ cells from (A-B) control and (C-D) ATZ-derived testes. DNA content of cells was evaluated by propidium iodide incorporation (PI), which discriminates among the four populations: M1, spermatozoa; M2, haploid cells; M3, diploid cells; and M4, tetraploid cells (meiotic cells). (E) Quantitation of the percentage of cell type in each population from F3 control (white bars) and ATZ-derived mice (grey bars); n=8. The details of the flow cytometric analysis of germ cells are described in the Supplementary Methods section.

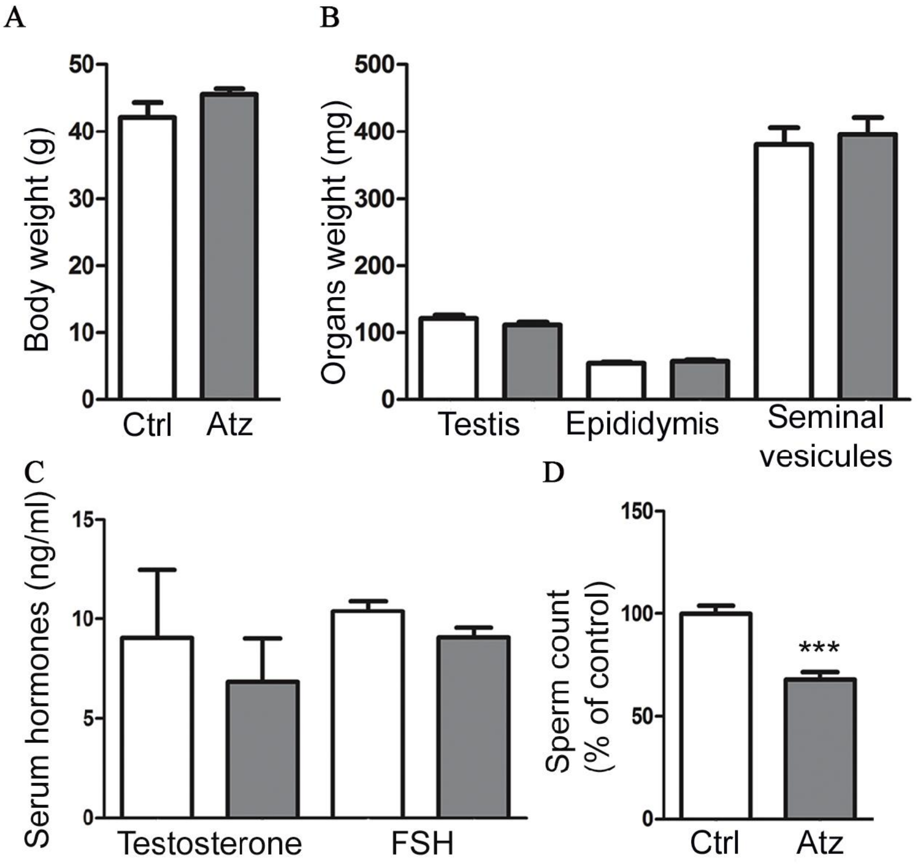

Supplementary_Figure_S4. Embryonic exposure to ATZ does not affect the weight of reproductive organs but decreases spermatozoa number in the epididymis in the F3 generation of males after treatment.

Weight of (A) body and (B) reproductive organs in F3 control (with bar) and ATZ (grey bar) mice (n≥14). (C), Testosterone and FSH level are not significantly decreased in serum of F3 control and ATZ-derived mice (n≥8). (D) The number of spermatozoa in the epididymis decreased in F3 ATZ-derived males compared to the control (n ≥10). Significant differences (P***<0.001, t-test) shown by asterisks. The measurements of hormones and spermatozoa counts are described in the Supplementary Methods section.

A

B

C

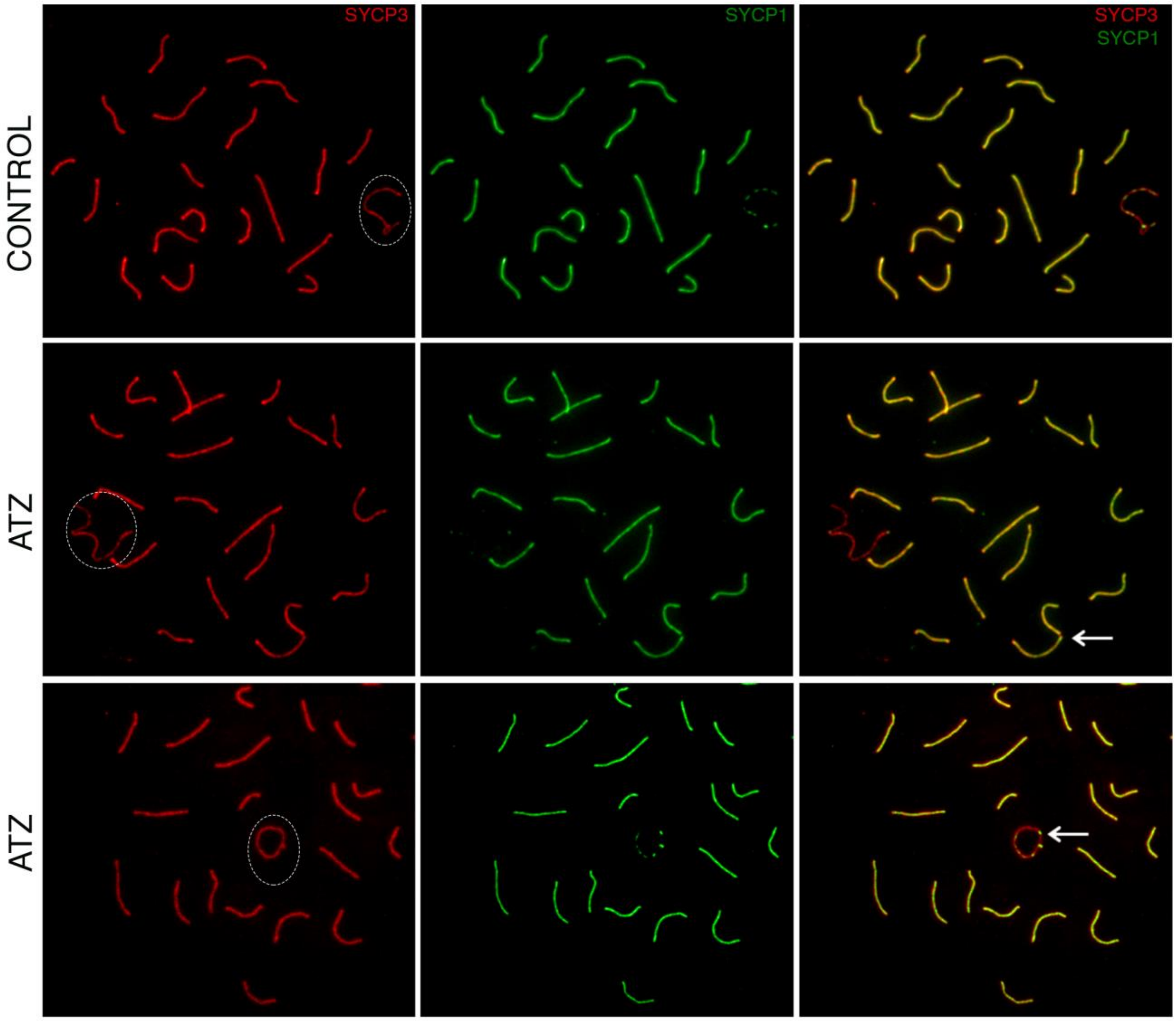

D

	Normal SCs	Telomere defects	"ring" sex body	Incomplete synapse
control, n=199	185	10	0	4
ATZ, n=195	156	25	10	4

Supplementary_Figure_S5. Embryonic exposure to ATZ affects meiosis in F3 ATZ-derived males.

Surface spreads were prepared from F3 generation testes of the control or ATZ-derived males from 4 independent experiments as described in Supplementary Methods section. The spreads were immunostained against the major protein of lateral element of synaptonemal complex (SC), SYCP3 (in red), and against protein of the SC central element, SYCP1 (in green). (A) Representative images of control testis spread, normally SYCP3 is detected along the length of every chromosomes, SYCP1 is present in all autosomal chromosomes and is visible as punctuated or absent immunostaining pattern in sex chromosomes, (B) In ATZ-derived males the cells with anomalies of the telomere connections are increased (C) the formation in sex chromosomes of the “ring” like structure. X and Y sex chromosomes (sex body) is circled, the arrow shows the chromosomes with abnormal phenotypes.
N=199 (control), n=195 (ATZ), $p<0.01$, Pearson's chi-squared test.

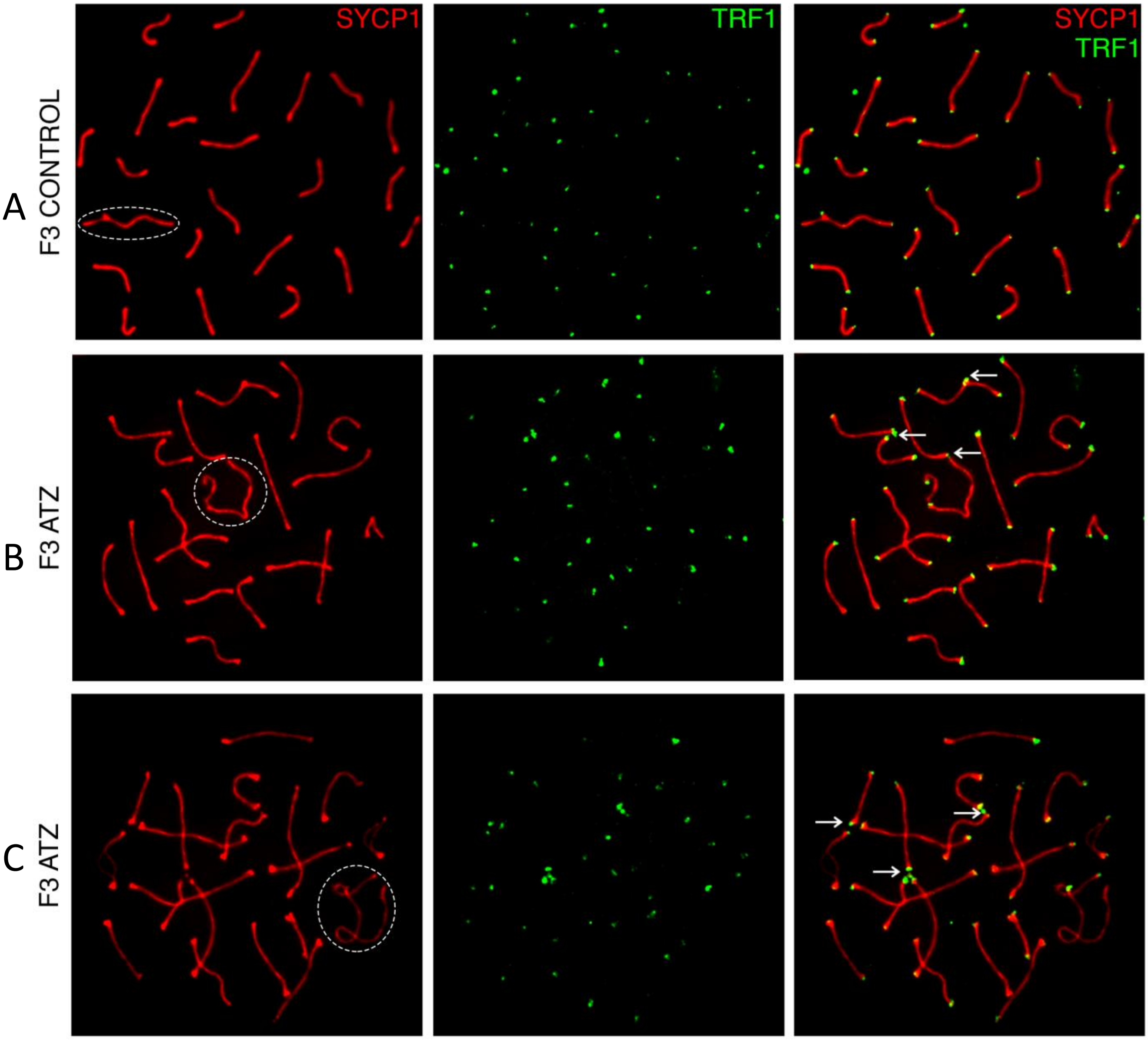

Supplementary_Figure_S6. Embryonic exposure to ATZ causes defects in SC in F3 ATZ-derived males.

Surface spreads were prepared from the testes of the F3 generation of control or ATZ-derived males as described in Supplementary Methods section. The spreads were immunostained against the major protein of synaptonemal complex, SYCP3 (in red), and TERF1 (in green), a telomere –binding protein. (A) In control cells the TERF1 signal is detected at the end of each chromosomes. (B) The telomeres of different chromosomes are connected in cells of ATZ-derived males . (C), The telomeres of different chromosomes were connected in ATZ-derived sample , X and Y sex chromosomes (sex body) are circled, the arrows show the chromosomes with abnormal phenotypes.

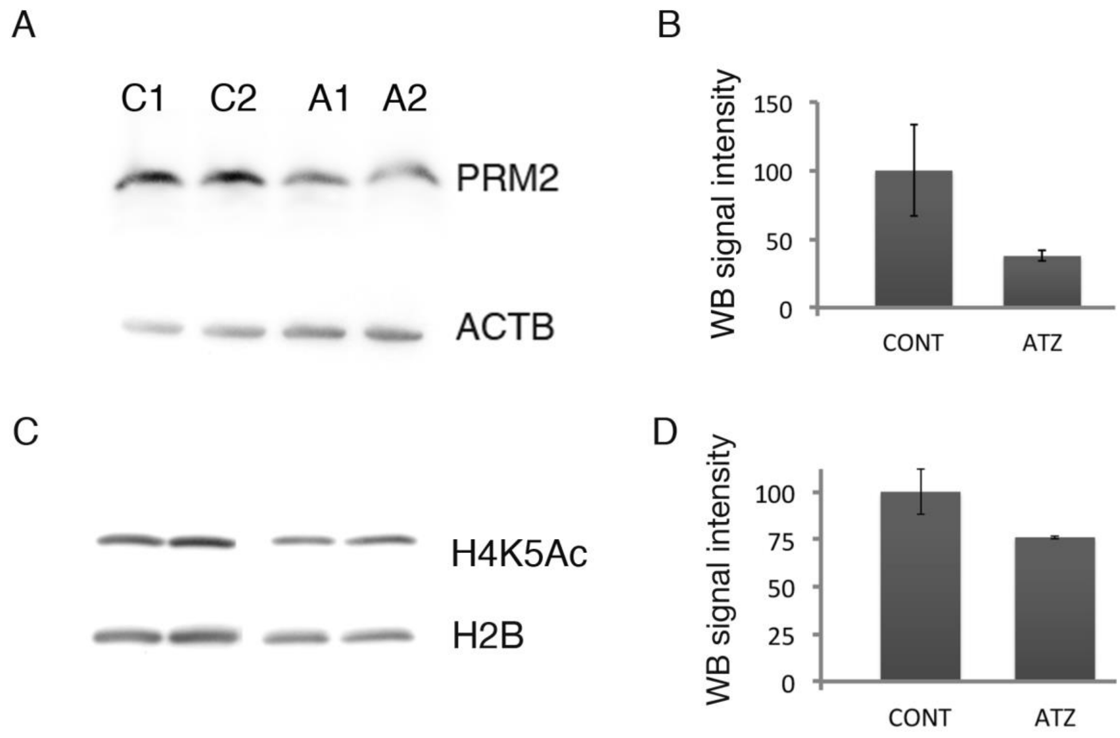

Supplementary_Figure_S7. The PRM2 and H4K5Ac proteins are decreased in F3 ATZ progeny males.

The total protein extracts or purified histone fractions from F3 control or ATZ progeny testes were prepared as described in Supplementary Methods. Two biological replicates of protein extracts were used. (A) The signal of PRM2 intensity was normalized to ACTB, (B) quantitative analysis of the blot in A. (C) The signal of H4K5Ac intensity was normalized to intensity of unmodified histone H2B, (D) quantitative analysis of the Western blot in C. The peak intensities was calculated using Image J software.

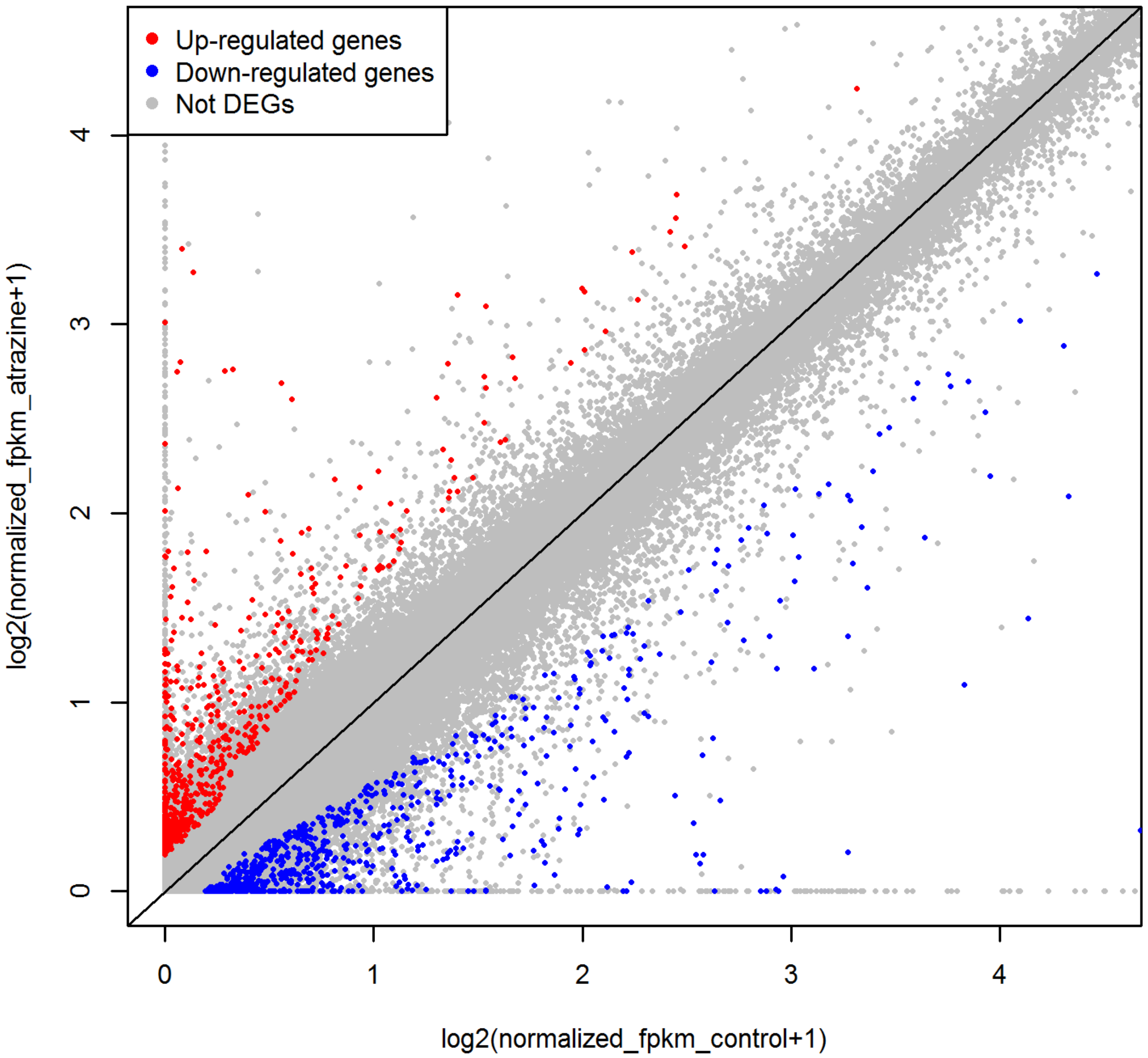

Supplementary_Figure_S8: **Embryonic exposure to ATZ globally affects gene expression in the testis tissue in third generation of males**

The scatter plot of differentially expressed genes. Each dot is the log2 mean normalized FPKM (fragments per kilobase of exon per million mapped fragments) value from three biological replicates. Up-regulated transcripts are depicted in red, down-regulated ones in blue and not differentially expressed transcripts are in grey, (619 transcripts are upregulated and 703 downregulated).

A

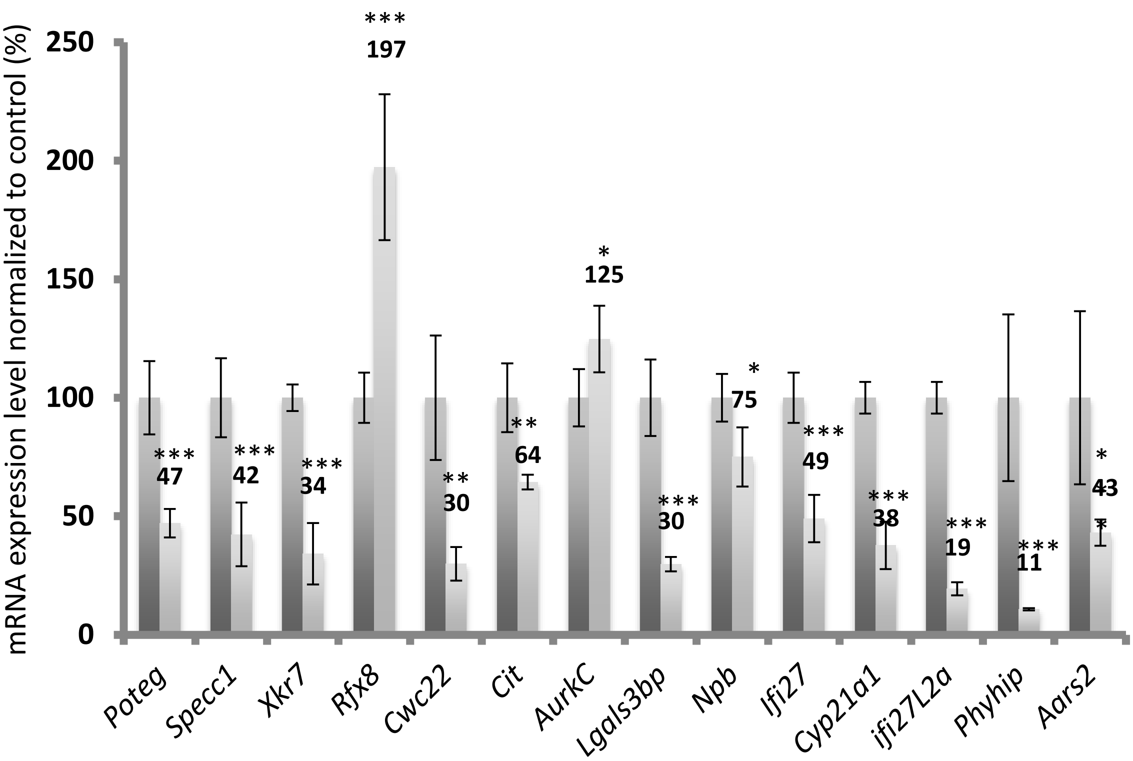

B

gene	RNA-seq FC	FDR RNA-seq	qPCR FC	p-value qPCR	process
Poteg	0.49	0.013	0.47	0.00346	Spermatogenesis
Specc1	0.46	0.017	0.42	0.00196	Spermatogenesis, role in cancer
Xkr7	0.48	0.050	0.34	0.00066	ND
Rfx8	0.42	0.001	0.42	0.00503	Transcription
Cwc22	0.27	0.019	0.30	0.01010	Splicing
Cit	0.00	0.025	0.64	0.01406	Spermatogenesis
AurkC	3.48	0.023	1.25	0.03762	Spermatogenesis
Lgals3bp	0.22	0.008	0.30	0.00256	Cell adhesion
Npb	0.47	0.044	0.75	0.02218	G-protein coupled receptor b
Ifi27	0.33	0.019	0.49	0.00044	Regulation of transcription
Cyp21a1	0.23	0.034	0.38	0.00011	C21-steroid hormone biosynthetic
Ifi27L2a	0.10	0.049	0.19	0.00503	Immune response
Phyhip	0.01	0.032	0.11	0.00002	Mitophagy
Aars2	0.45	0.040	0.43	0.01473	Mitochondrial respiratory chain complex assembly
Spin2d	7.59	0.007	10.69	0.05092	ND
Crtam	>10	0.042	4.71	0.00878	Regulation of immune response

Supplementary_Figure_S9. Comparison of RNA-seq data and qPCR gene expression data for mRNA analysis.

(A) Differentially expressed genes identified by RNA-seq and confirmed by qPCR. Total RNA was extracted, cDNA isolated and qPCR performed as described in the Methods section. Primer sequences are indicated in Supplemental_Table_S12. At least 4 independent experiments were performed and averaged (* p<0.05, ** p<0.01, *** p<0.001, t-test). Control samples are in dark grey, ATZ-derived in light grey. (B) Comparison of gene expression determined by RNA-seq and qCPR data.

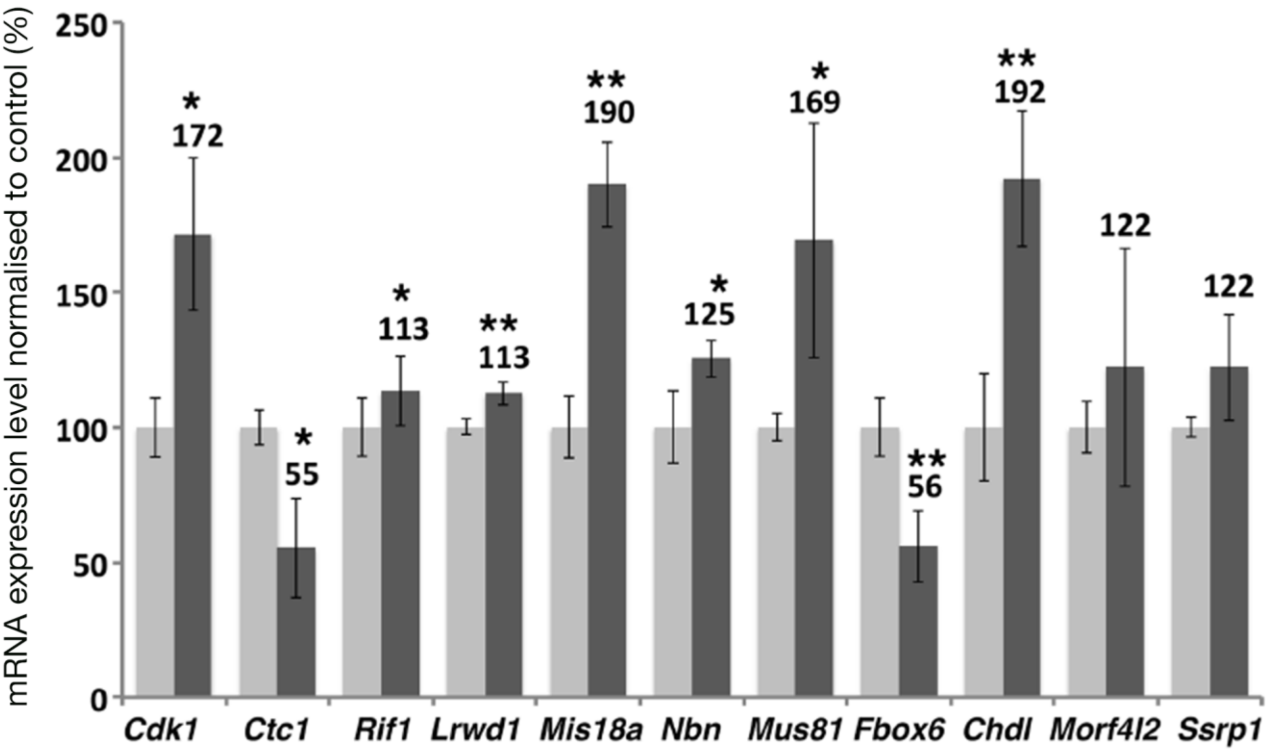

Supplementary _Figure_S10. **Embryonic exposure to ATZ affects the expression of several genes belonging to the DNA damage pathway.**

QPCR confirmation of differentially expressed genes of DNA damage response. The details of the experiments are described in the Methods section. The data represent the average of at least 4 independent experiments and are expressed as mRNA expression level in the experimental sample compared to the control (%) (p< 0.05, ** p < 0.01, ***, p< 0.001, t-test). The primer sequences are indicated in Supplemental_Table_S12.

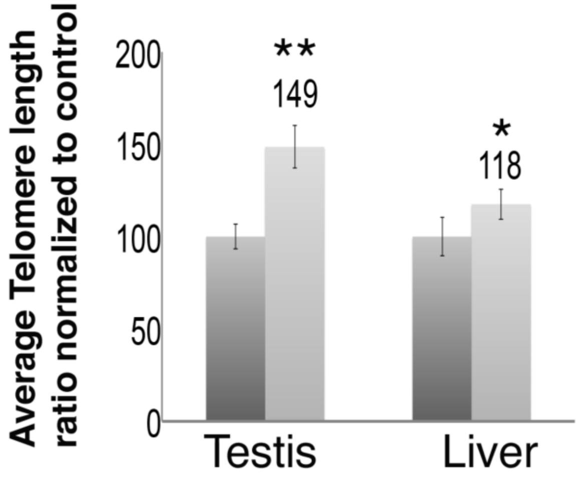

Supplementary_Figure_S11. Embryonic exposure to ATZ affects the telomeres length.

Genomic DNA was isolated from testis or liver tissues using Proteinase K and phenol: chloroform extraction. Concentration of DNA was estimated by fluorescent method. Equal amounts of DNA were used for qPCR. The primers sequences are indicated in Suplementary_Table_S12.

Data from at least 6 independent experiments were averaged and expressed as the average telomere length (ATLR) normalized to control, (* $p<0.05$, ** $p<0.01$, t-test).

A

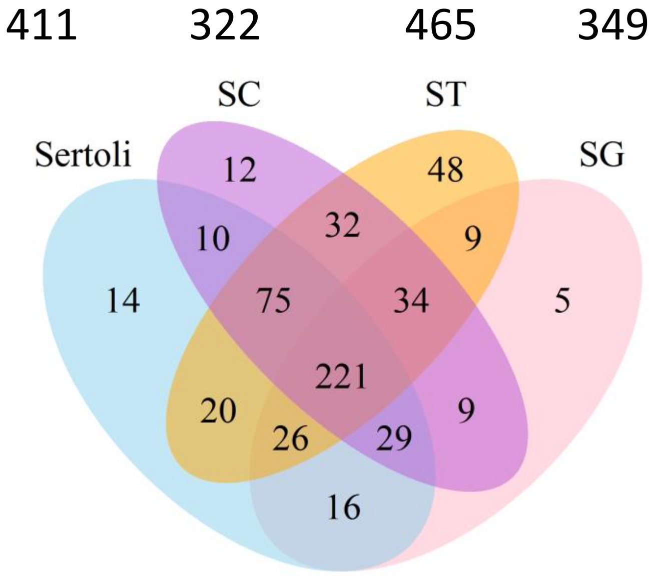

B

**Gene symbol**	**Gene Name**
2810474O19Rik	RIKEN cDNA 2810474O19 gene
Acp6	acid phosphatase 6, lysophosphatidic
Aox3	aldehyde oxidase 3
Esrrb	estrogen related receptor, beta
Flnb	filamin, beta
Gatad2b	GATA zinc finger domain containing 2B
Gtf2i	general transcription factor II I
Hsp90aa1	heat shock protein 90, alpha
Islr2	immunoglobulin superfamily containing leucine-rich repeat 2
Manba	mannosidase, beta A, lysosomal
Mcf2	mcf.2 transforming sequence
Ncor1	nuclear receptor co-repressor 1
Prrc2b	proline-rich coiled-coil 2B
Rfc1	replication factor C (activator 1) 1
Rfc5	replication factor C (activator 1) 5
Rif1	replication timing regulatory factor 1
Ssrp1	structure specific recognition protein 1
Sumo3	small ubiquitin-like modifier 3
Esrrb	estrogen related receptor, beta

Supplementary_Figure_S12: **The origin of differentially expressed testis genes in the third generation of males after treatment**.

A). The differentially expressed genes were compared with gene expression in different cell types from previously published dataset (Gan, Wen et al. 2013). Each oval represents a fraction, Sertoli (Sertoli cells), SC (spermatocytes), ST (spermatids), SG (Spermatogonia). A large number of genes (221) are shared between all cell fractions. The highest number (48) of unique differentially expressed genes are found in spermatid fraction. B). DEGs from spermatogonia fraction are known to be interacting with POU5F1.

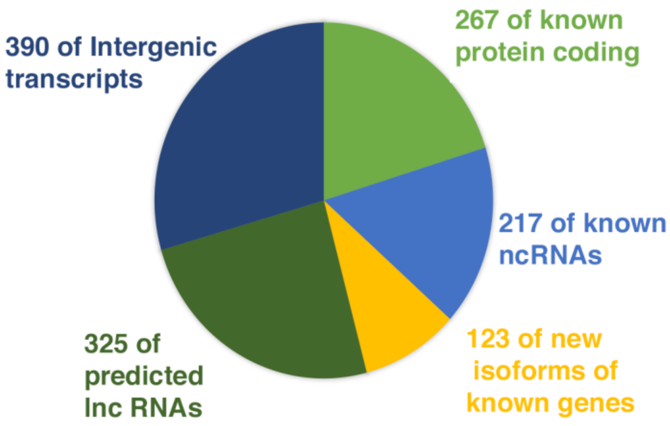

Supplementary_Figure_S13. Group of transcripts that are differentially expressed in the F3 generation of the ATZ -derived males.

The pie chart shows the distribution of the differentially expressed RNA types in the testis tissue of F3 ATZ-derived males. The RNA-Seq data were analyzed as described in the Methods section.

A

Term Name	Number of genes	P-Value	Genes
transcription regulator activity	32	1.77E-04	*Sox2, Sox5, Sox14, Meis1, Max, Lbh, Olig3, Gata3, Gata4, Hey2, Foxb2, Atoh8, Foxd1, Tlx1, Klf5, Klf12, Tbx3, , Nr4a2, Rybp, Tead1, Ldb2, Arntl, Isl1, Med13l, Ppargc1a, Med10, Mef2d, Hhex, Ets1, Vgll3, Prdm2, Rbpj*
nitrogen compound biosynthetic process	13	1.07E-03	*Bcat1, Dctd, Adss, Nampt, Adcy1, Atp11b, Nr4a2, Atp5g3, Cmpk2, Gm4953, Gata3, Mat2b, Tph2*
cell motion	13	5.32E-03	*Fut8, 4921509c19rik, Plxna2, Gm14147, Nr4a2, Tnp1, Isl1, Kitl, Alcam, Tns1, Bmp7, Foxd1, Chl1*
regulation of cell development	8	6.82E-03	*Tbx3, Ntrk2, Sox2, Sox5, Isl1, Bmp7, Meis1, Ngf*
regulation of organ growth	4	1.06E-02	*Gata4, Hey2, Cxadr, Bcl2l11*
potassium ion binding	6	1.90E-02	*Kcnma1, Slc24a3, Kcnh7, Kcnj3, Kcnip4, Kcnv1*

B

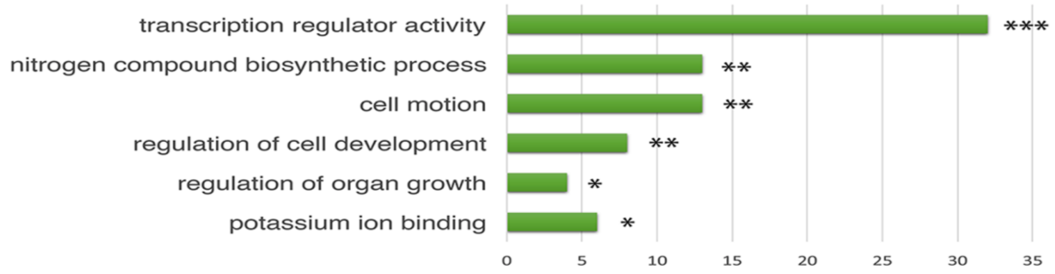

C

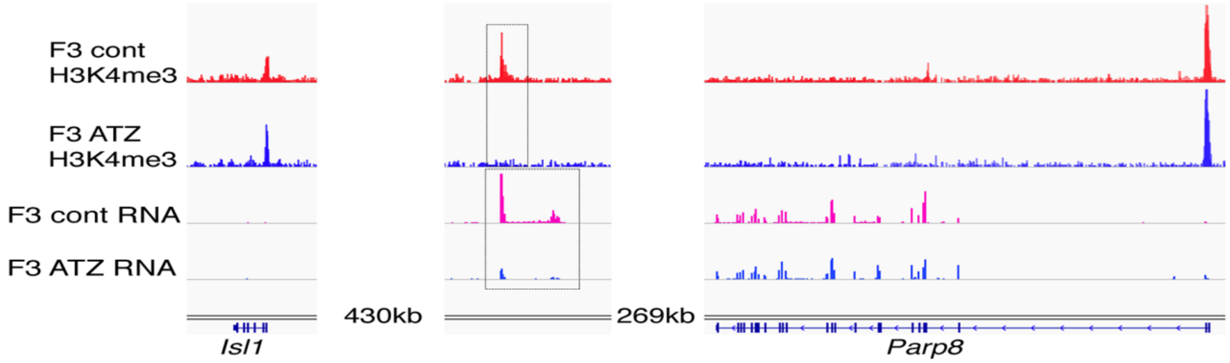

Supplementary_Figure_S14: The expression of the new long noncoding RNA is globally affected in the F3 generation.

(A) Gene Ontology (GO) term enrichment of genes located upstream and downstream of LincRNA. GO terms were sorted based on p-values (*P<0.05; ** P< 0.01, *** P<0.001, Fisher exact test). (B) The plot represents the number of genes in each GO category. (C) A new predicted long noncoding RNA is located distally from the *Isl1* and *Parp8* genes. Both the expression of noncoding RNA and the H3K4me3 occupancy in the promoter are dramatically decreased. H3K4me3 peaks and RNA expression were determined as described in the Methods section and represent the averaged and normalized values of two (ChIP-seq) or three (RNA-seq) biological replicates for the F3 control (in red) or ATZ-derived (in blue) males testes.

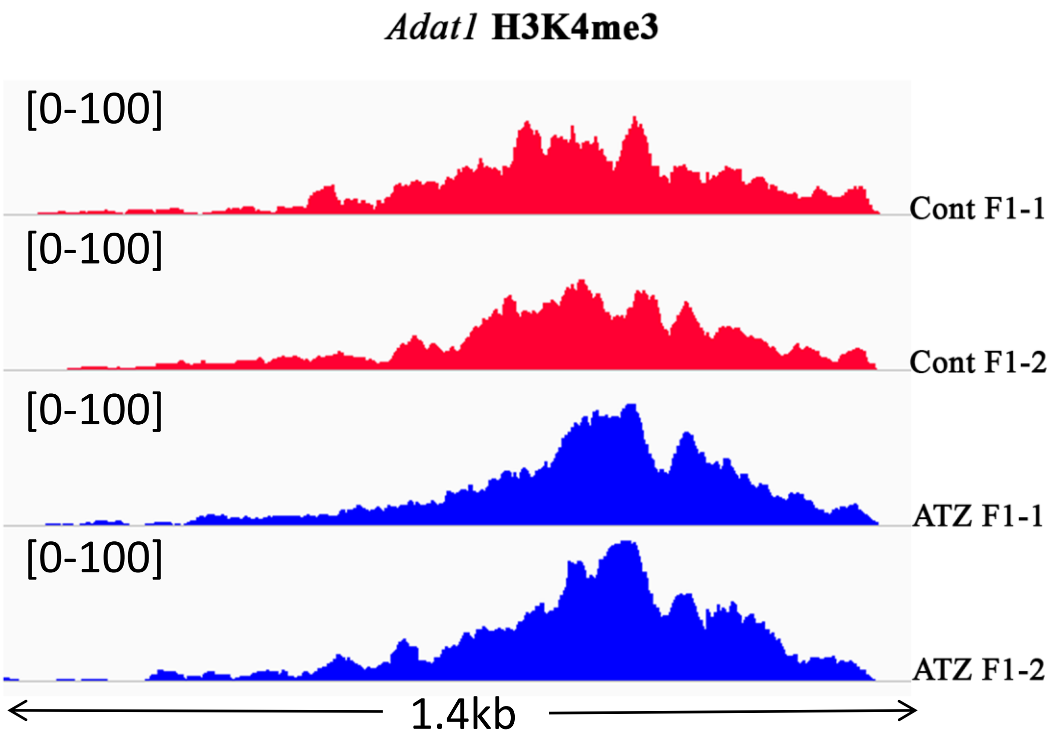

Supplementary_Figure_S15. Embryonic exposure to ATZ affects the H3K4me3 regions in promoters of *Adat1* gene in F1 ATZ-derived males.

Histone H3K4me3 occupancy is changed in the promoter of the *Adat1* gene in the testes of the F1 generation of ATZ-derived males. Each plot represents the normalized number of tags in the control (red) and ATZ-derived samples (blue).
Two biological replicates are shown for the control and the ATZ-derived animals. The IGV genome viewer v 2.3.36 was used to visualize the ChIP-seq data for the *Adat1* gene.

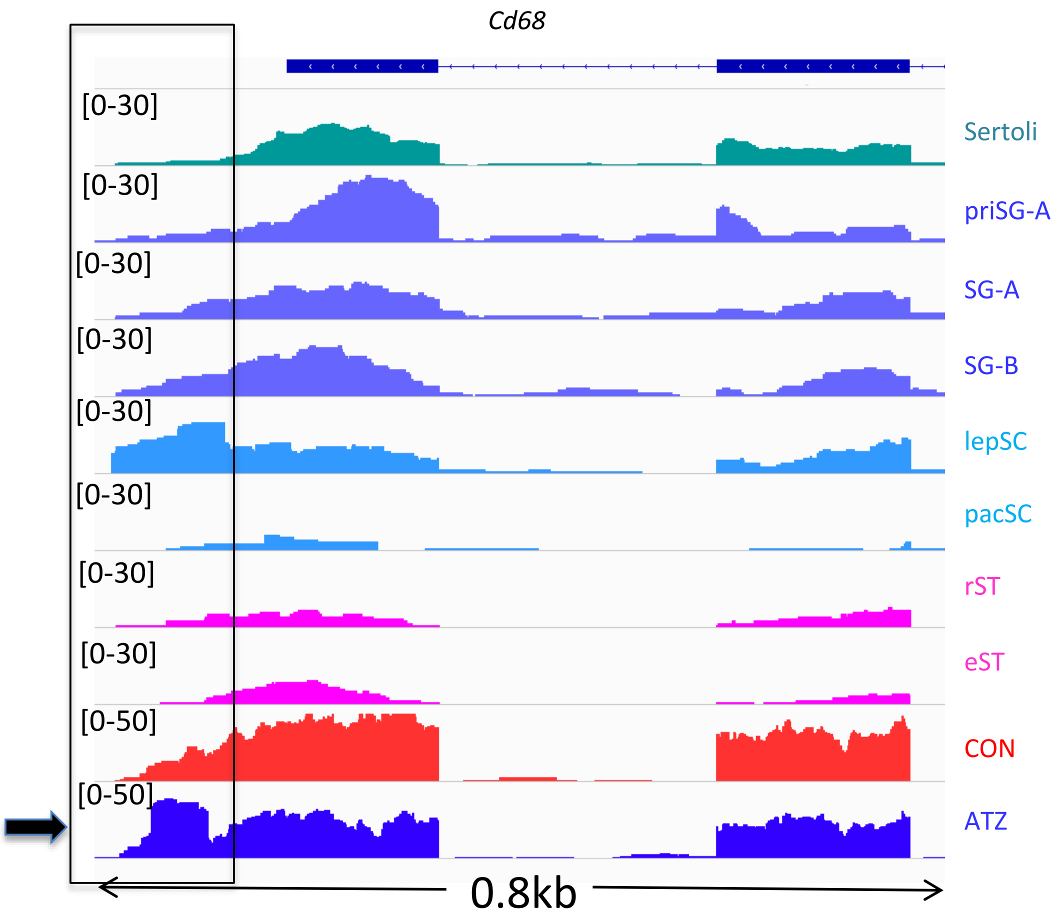

Supplementary_Figure_S16. The altered APA transcript of the *Cd68* gene was identified in F3 ATZ progeny males.

The RNA profiles from different cell types based on previous work were reanalyzed (Gan, Wen et al. 2013). The *CD68* gene has only one transcript. The altered polyadenylated site is shown by an arrow. Three biological replicates of RNA-seq data in *Cd68* transcripts were merged for simplicity in the control (red) and ATZ-derived (blue) transcripts and are represented as averaged and normalized tag numbers. The direction of transcription is on the opposite strand. priSG-A: primitive type A spermatogonia; SG-A: type A spermatogonia; SG-B: type B spermatogonia; lepSC: leptotene spermatocytes; pacSC: pachytene spermatocytes; rST: round spermatids; eST: elongative spermatids; CON: F3 testis, control; ATZ: F3 testis, atrazine.
The IGV genome viewer v 2.3.36 was used to visualize the RNA-seq data.

A

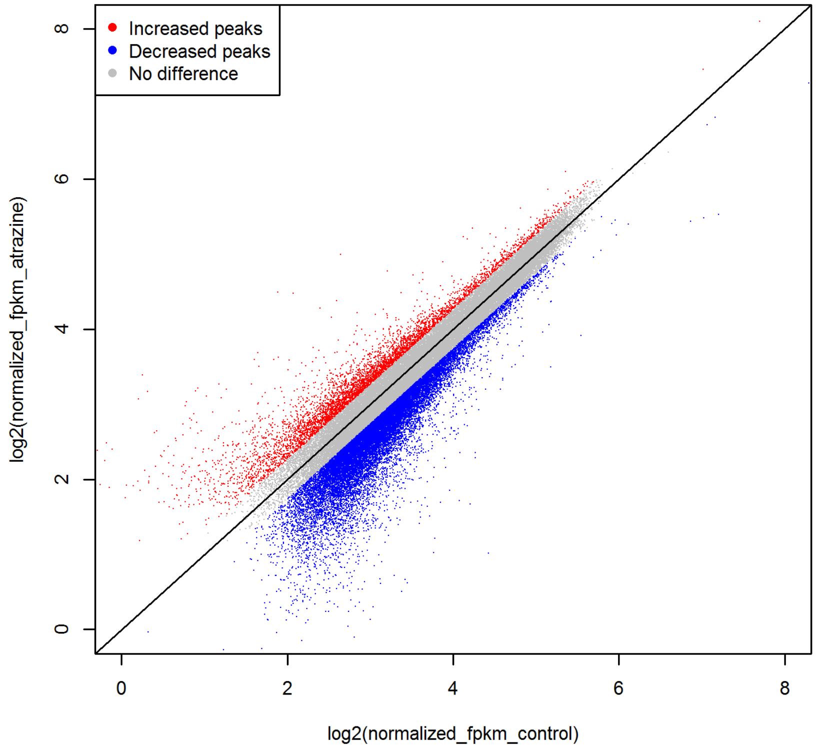

B

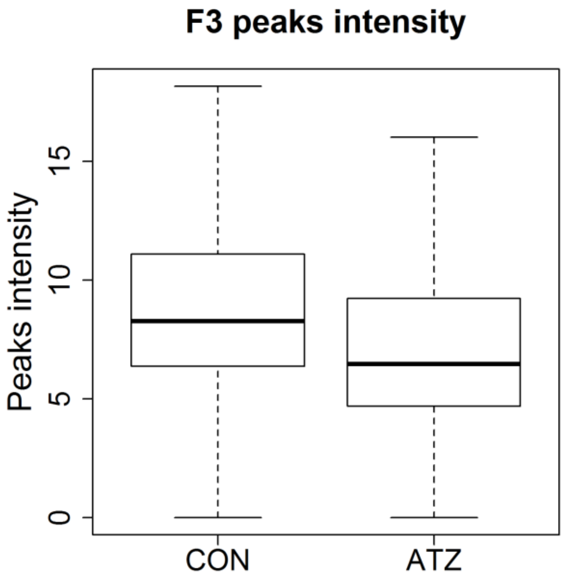

Supplementary_Figure_S17. The scattered plot and boxplot of H3K4me3 peaks with FC above 1.2.

(A) The scattered plot of differential peaks. With fold change above 1.2, we found 5010 peaks have increased and 19479 peaks have decreased H3K4me3 occupancy. Each dots represents the log2 of mean normalized FPKM value from two biological replicates. The increased occupancy peaks are depicted in red, decreased occupancy peaks are in blue, peaks with no changes in H3K4me3 occupancy are shown in grey color. (B) Boxed plot of H3K4me3 peaks intensity

A

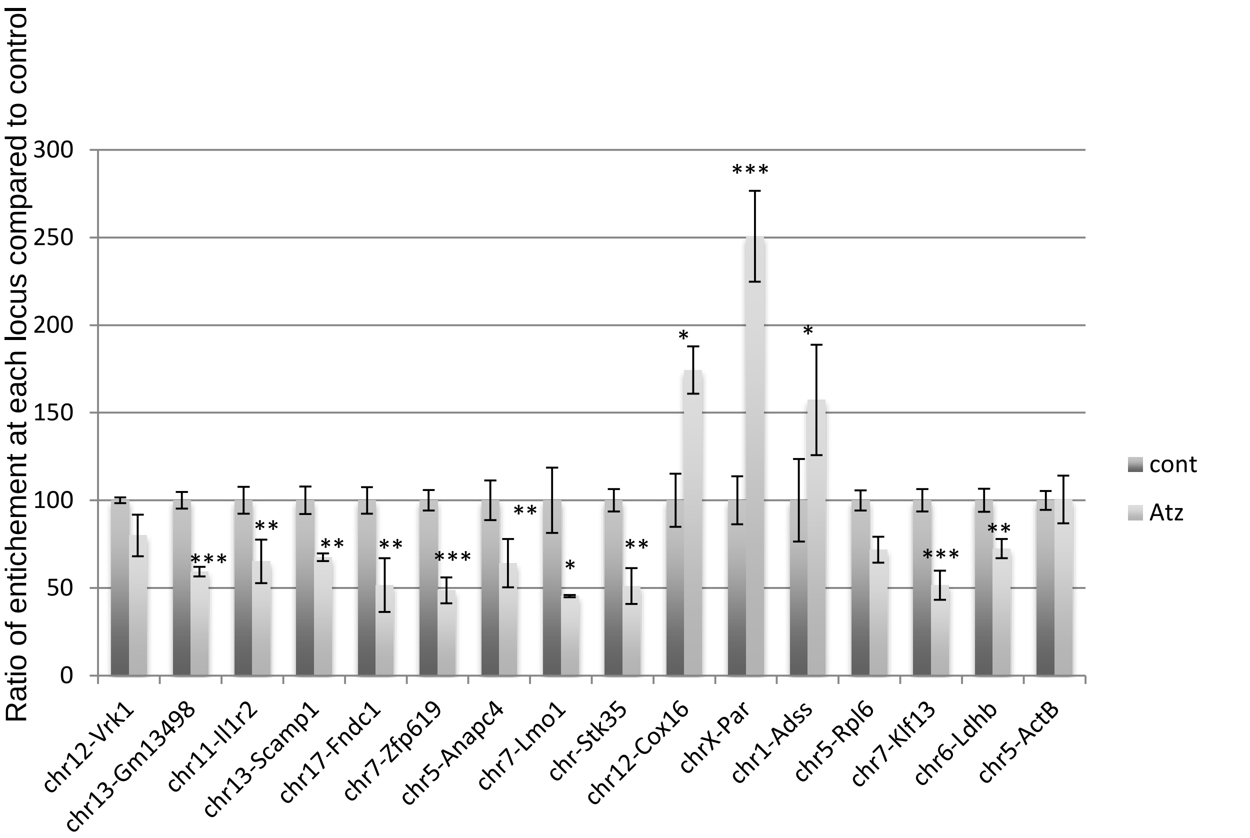

B

region	start	end	Chip-Seq FC	FDR-value	qPCR FC	qPCR P-value
chr12-*Vrk1*	107621161	107622130	0.62	0.06	0.80	0.060
chr13-*Gm13498*	50928661	50929100	0.46	0.04	0.59	0.000
chr11-*Il1r2*	40268641	40269441	0.65	0.05	0.65	0.009
chr13-*Scamp1*	94993331	94995910	0.56	0.06	0.67	0.004
chr17-*Fndc1*	8139441	8139720	0.57	0.06	0.52	0.006
chr7-*Zfp619*	46985121	46986110	0.60	0.07	0.49	0.000
chr5-*Anapc4*	53276871	53278250	0.35	0.05	0.64	0.014
chr7-*Lmo1*	116384801	116386450	0.30	0.03	0.45	0.015
chr-*Stk35*	129660271	129661171	0.66	0.05	0.51	0.001
chrX-*Par*	166434961	166435280	1.73	0.07	2.51	0.000
chr1-*Adss*	179913581	179913970	1.60	0.03	1.57	0.048
chr7-*Klf13*	71200431	71202381	0.64	0.08	0.52	0.000
chr6-*Ldhb*	142464871	142466211	0.52	0.07	0.72	0.002

Supplementary_Figure_S18. Comparison of ChIP-seq and ChIP-qPCR data.

(A) ChIP-qPCR was performed as described in the Methods section. The primer sequences are indicated in Supplemental_Table_S12. The average of at least 4 independent experiments is plotted and expressed as the ratio of enrichment of ChIP product compared to Input and normalized to the control, *p<0.05, **p<0.01, ***, p<0.001, t-test. The control samples are in dark grey, the ATZ-derived in light grey.
(B) Comparison of ChIP-seq and ChIP-qCPR data

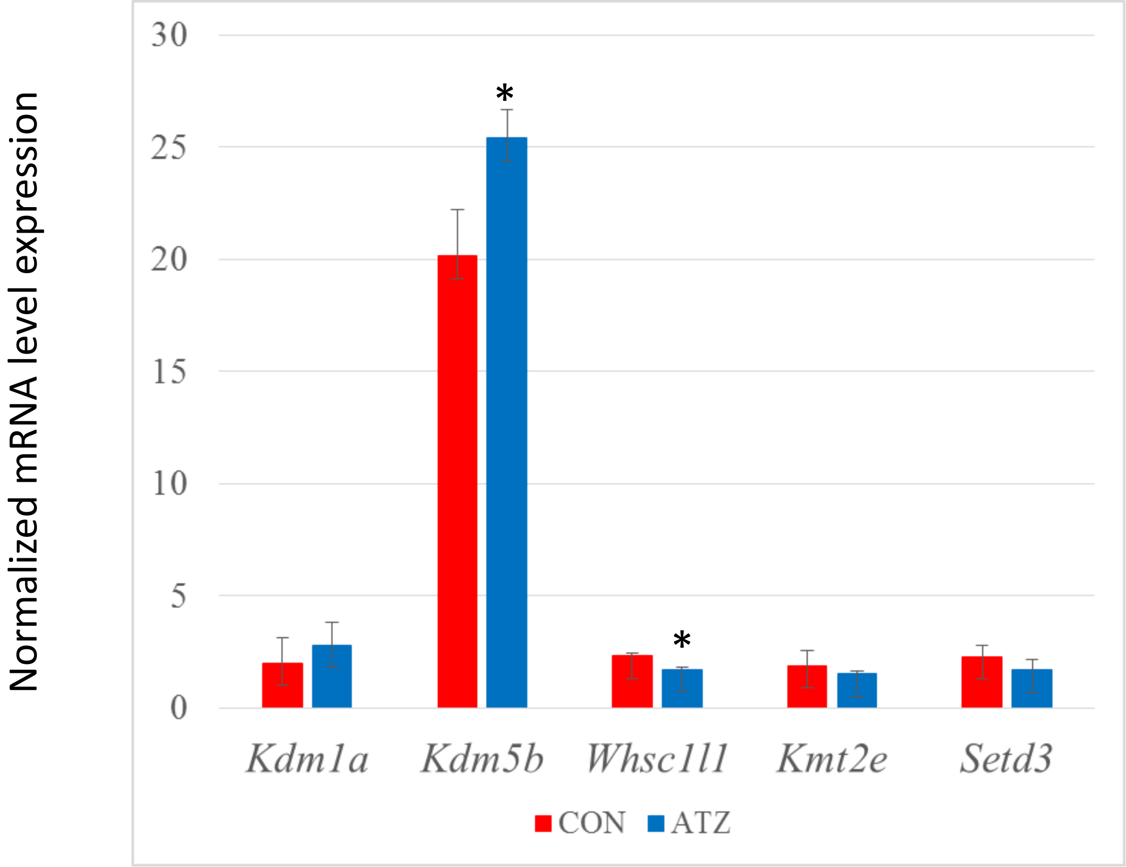

Supplementary_Figure_S19. The expression level of histone H3 lysine 4 methyltransferase mRNA (*Whsc1l1*) is decreased and the expression levels of histone H3 lysine demethylases (*Kdm5b*) is increased in the testes of F3 ATZ-lineage males.

The expression levels were determined using RNA-seq data. Each plot are represented as the averaged FPKM value of three biological replicates. FPKMs were calculated using the tag number. The control samples are in red, ATZ-derived samples in blue, *p<0.05, t-test.

A

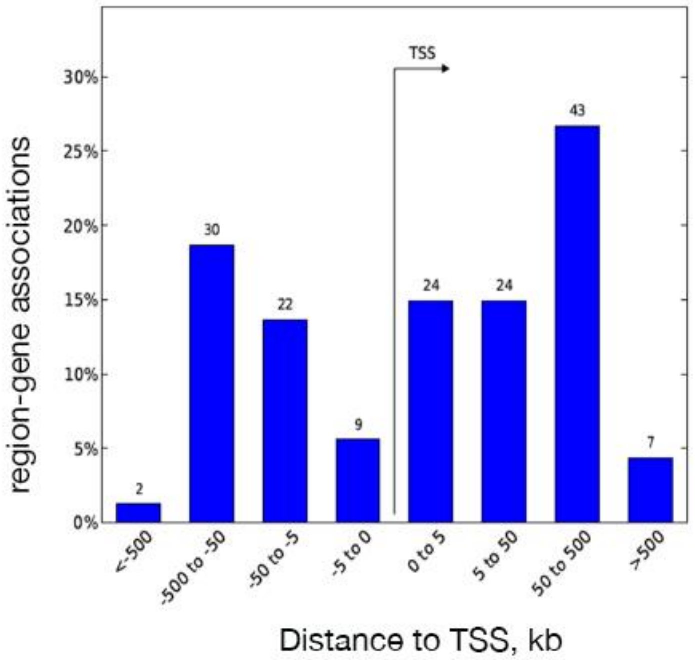

B

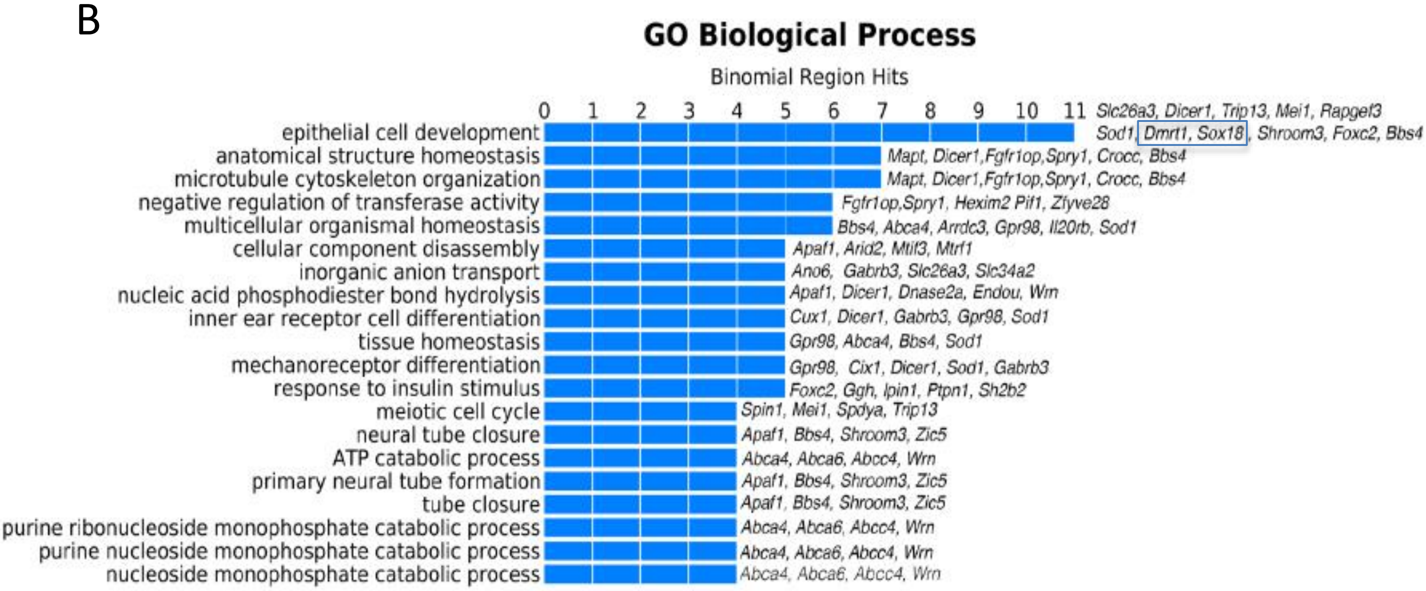

Supplementary_Figure_S20. Differential H3K4me3 peaks in spermatocyte fraction in testis of F3 ATZ-derived males are enriched in regions of genes involved in epithelial cell development and cytoskeleton organization

Differential H3K4me3 peaks were compared with normalized peaks intensity from purified spermatocytes or spermatids. (A) The distance of differential peaks to TSS of the genes, B) The subset of differential H3K4me3 peaks with high signal in spermatocytes were annotated with GREAT. The GO term of biological function is shown on the left, the corresponding genes are on the right.

A

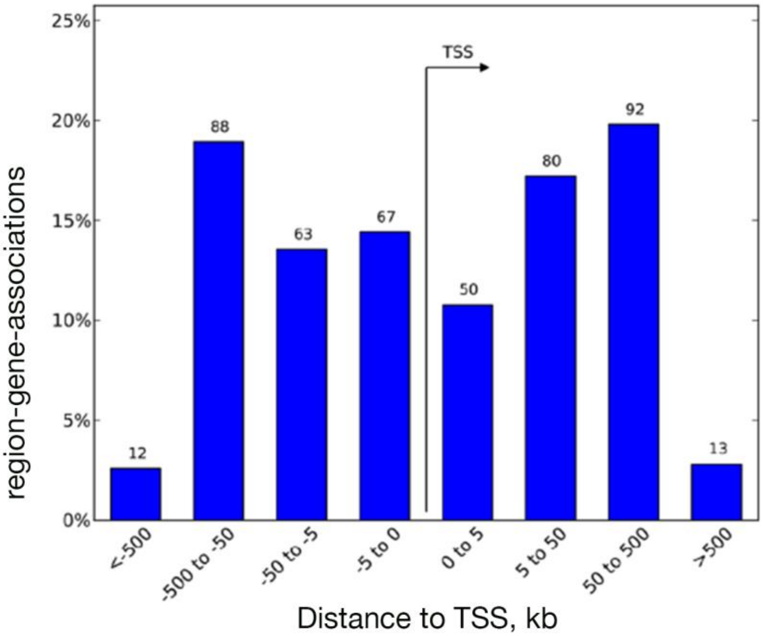

B

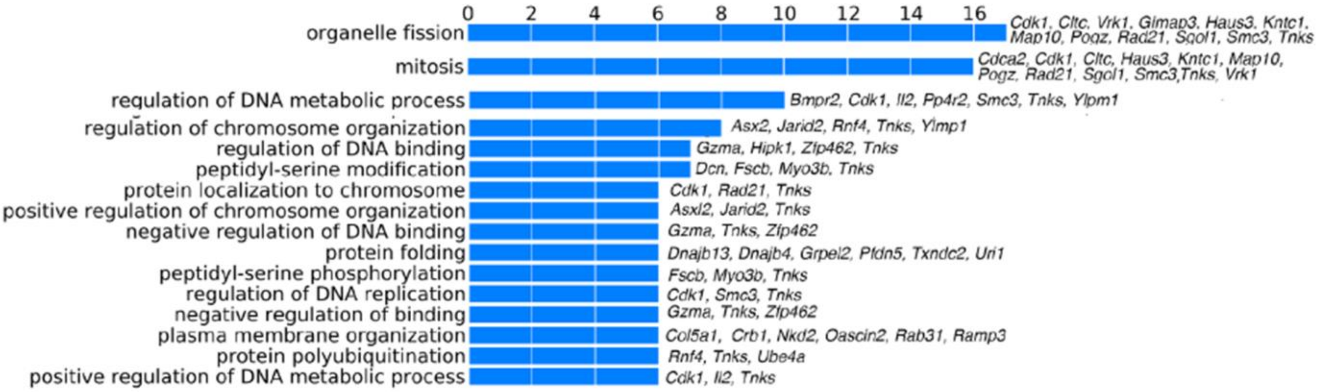

Supplemental_Figure_S21. Differential H3K4me3 peaks in spermatocyte fraction in F3 ATZ-derived males are enriched in regions of genes involved in mitosis.

Differential H3K4me3 peaks were compared with normalized peaks intensity from purified spermatocytes or spermatids. (A) The distance of differential peaks to TSS of the gene. (B) The subset of h3K4me3 peaks with high signal in spermatids were annotated with GREAT. The GO term of biological function is shown on the left, the corresponding genes are on the right.

A

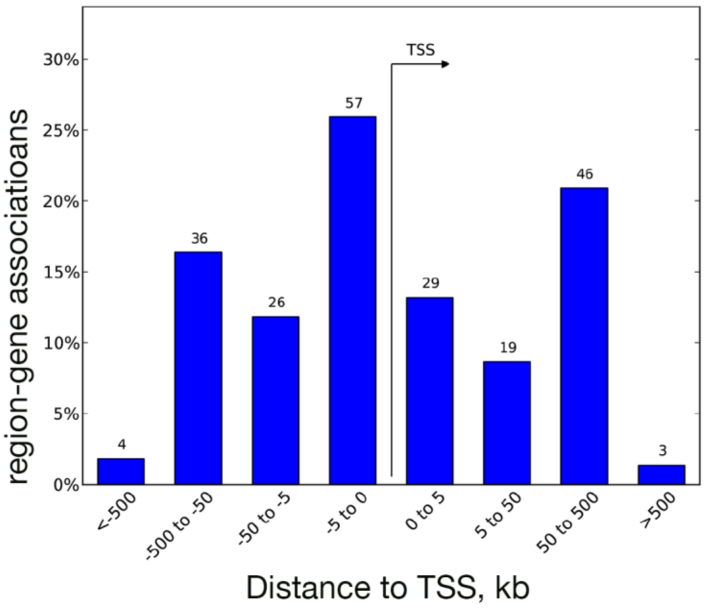

B

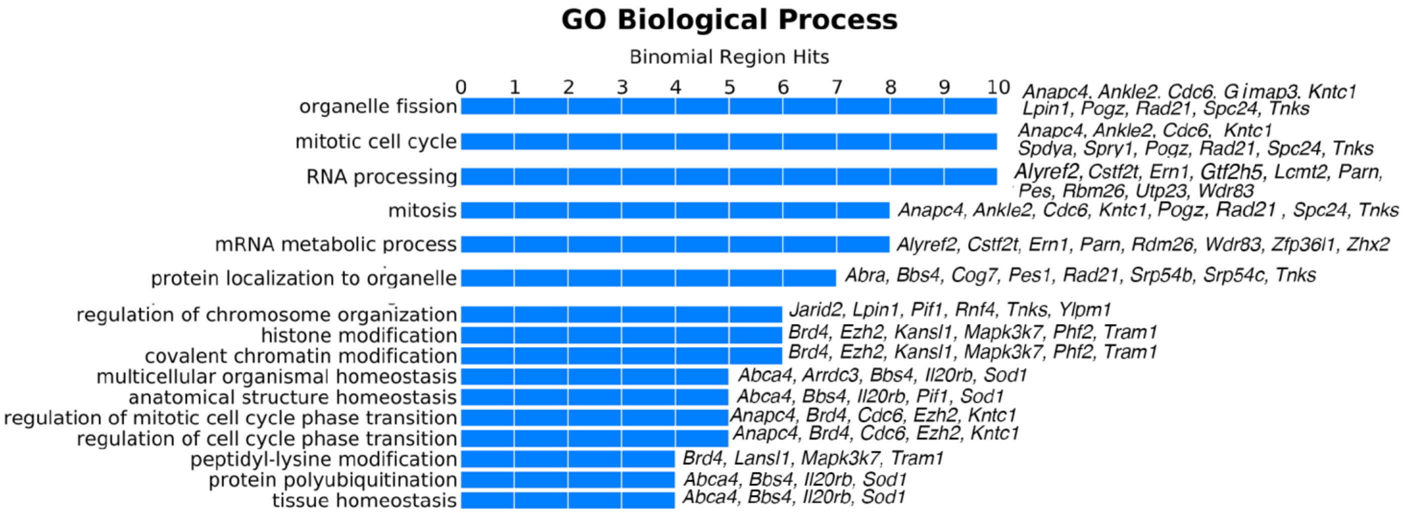

Supplementary_Figure_S22. Differential H3K4me3 peaks in spermatogonia fraction in F3 ATZ-derived males are enriched in regions for the genes associated with cell cycle, mitosis and RNA processing functions

Differential H3K4me3 peaks were compared with published datasets. (A) The distance of differential peaks to TSS of the genes, B) The subset of peaks with high signal in spermatogonia were annotated with GREAT. The GO term of biological function is shown on the left, the corresponding genes are on the right.

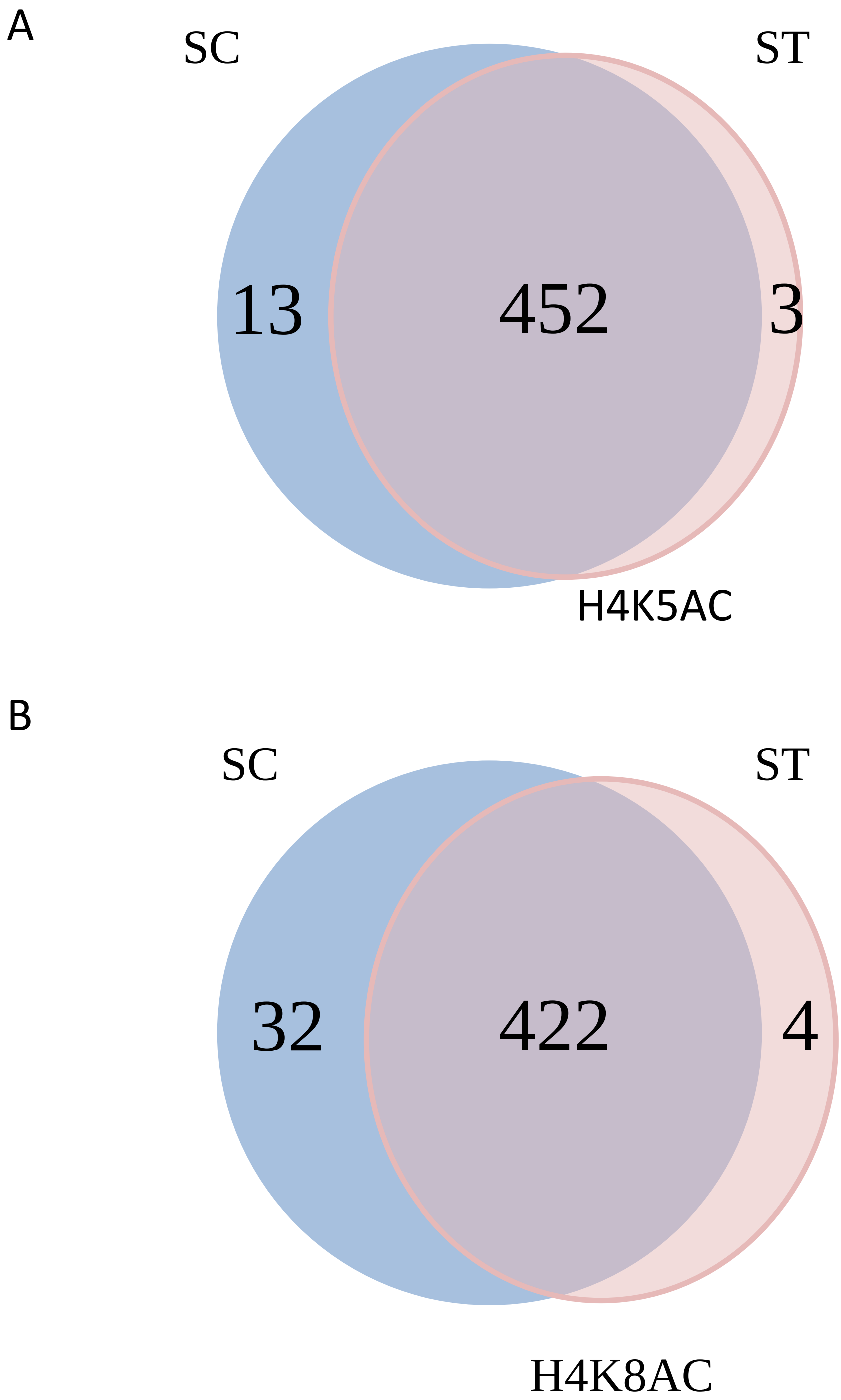

Supplementary_Figure_S23. The association of differential H3K4me3 peaks in F3 ATZ-derived males with H4K5Ac and H8K8ac marks from spermatocyte and spermatid fractions .

Differential H3K4me3 peaks from testis of F3 ATZ progeny ChIP-seq data were compared with published H4K5Ac and H4K8ac datasets. (A) The venn diagrams of differential peaks of testis fractions with H4K5Ac and (B) The venn diagrams of differential peaks of testis fractions with H4K8ac. SC(Spermatocytes), ST(Spermatids).

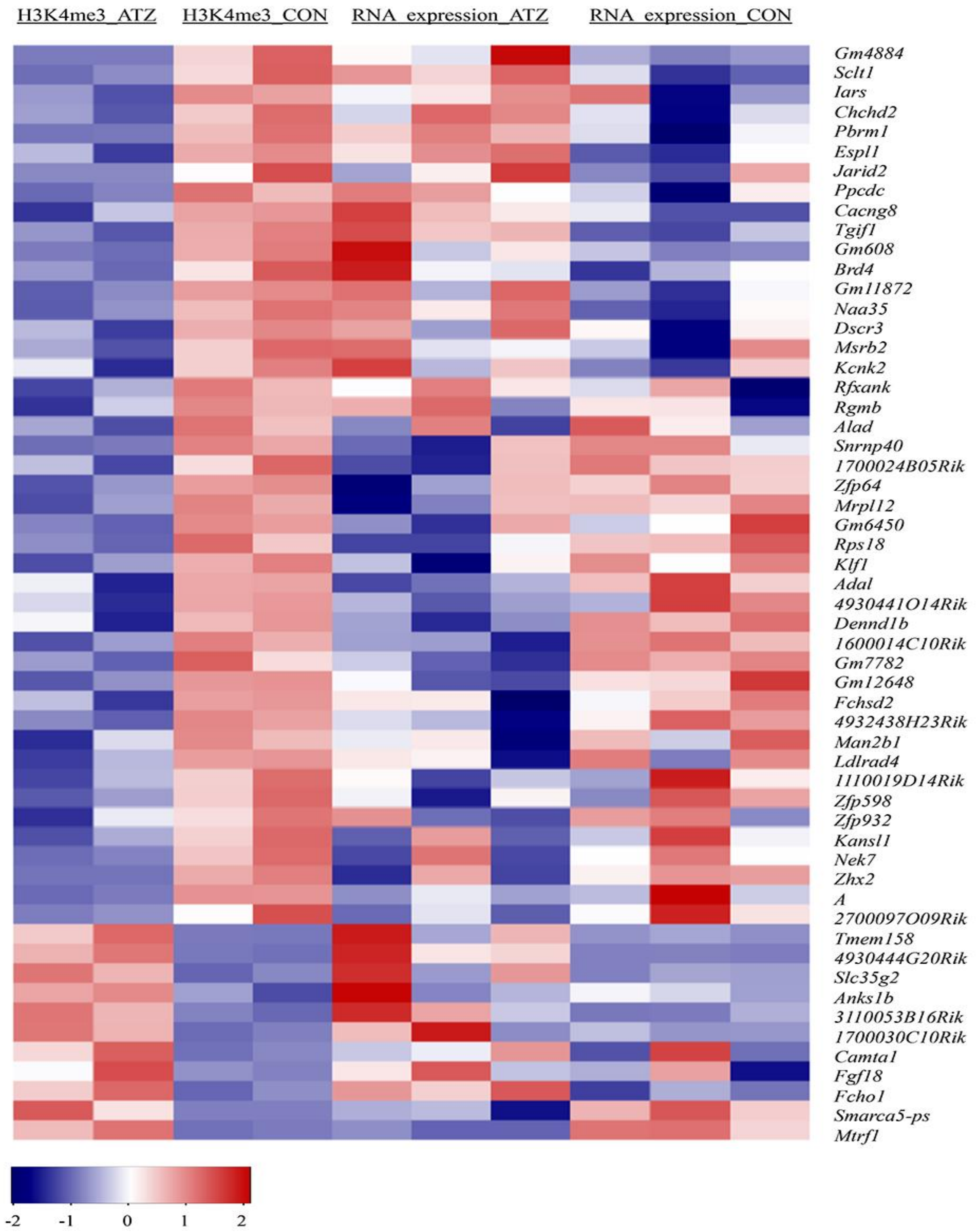

Supplementary_Figure_S24: **The association of intensity of differential H3K4me3 at promoters with altered gene expression level (FC>1.2) .**

H3K4me3 differential peaks associated with promoters were identified using published datasets based on signal ratio of H3K4me1 to H3K4me3. From all differential peaks , we found only 26% of them are associated with gene promoters. We presented here the signal for ChIP and corresponding RNA expression. Each column represents a biological replicate. Up-regulated genes and peaks with increased H3K4me3 occupancy are depicted in red, down-regulated RNA and peaks with decreased H3K4me3 occupancy in blue.

A

Chr	Start	End	*P*-val	FDR	peak presence in ATZ	Nearest Differentially expressed RNA	Fold Change RNA	type RNA
chr11	110041351	110042160	0,002	0,078	no	*Abca9*	1.2	coding, novel isoform
chr4	140820871	140821290	0,007	0,078	no	*Arhgef19*	0,7	coding, novel isoform
chr1	169171501	169171621	0,001	0,043	no	*Uck2*	0,6	coding, novel isoform
Chr8*	35142601	35144570	0,011	0,078	no	Intergenic RNA	0,6	novel isoform
chr10	21778021	21779480	0,006	0,065	yes	Intergenic RNA	21,0	novel isoform
Chr8	36322681	36324820	0,003	0,078	no	Intergenic RNA	0,8	novel isoform
chr10	21787571	21787970	0,002	0,043	yes	4930444G20Rik	3,0	protein coding
chr13	66815341	66815900	0,012	0,078	no	Intergenic RNA	0,8	novel isoform
chr7	46435861	46436150	0,000	0,073	no	Intergenic RNA	0,3	novel isoform

B

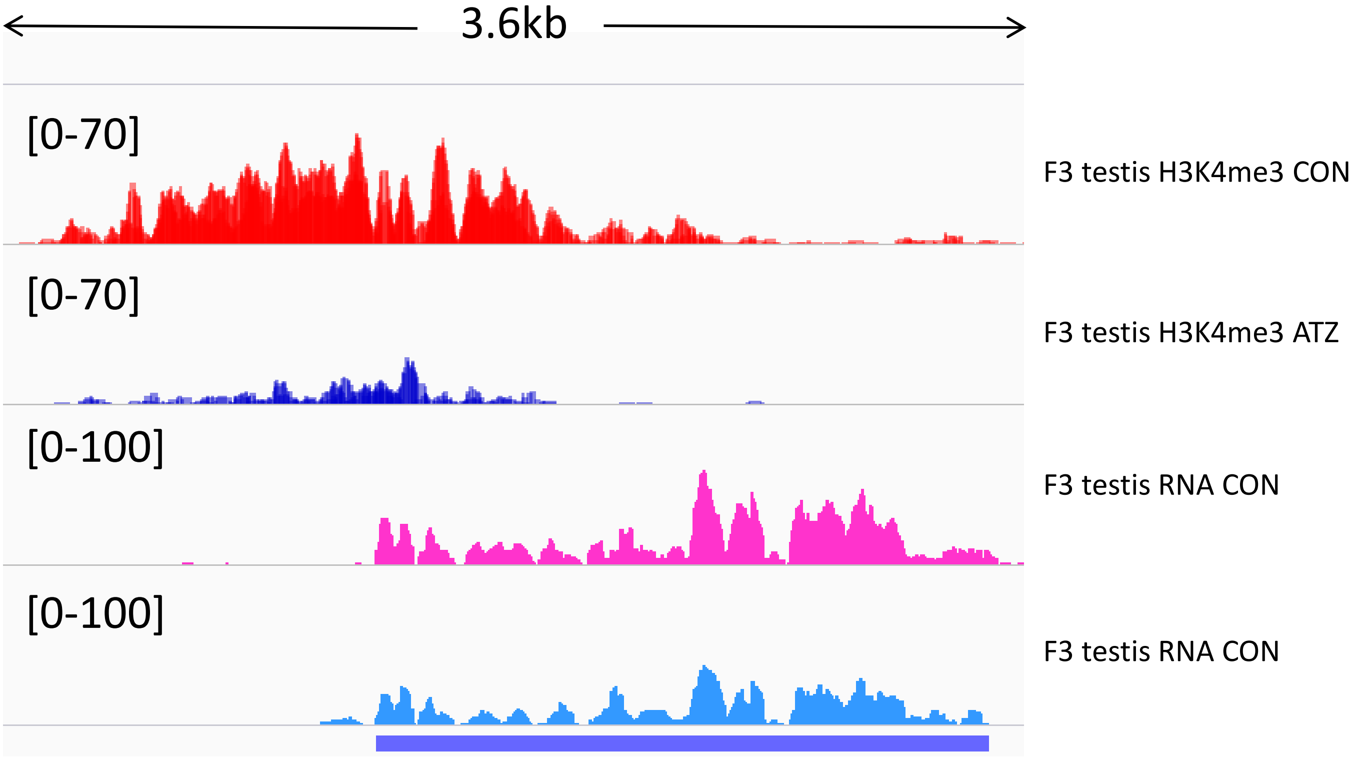

Supplementary_Figure_S25. Differential H3K4me3 peaks lost or gained in F3 ATZ-derived males

(A) Differential H3K4me3 peaks from testis of F3 ATZ progeny ChIP-seq data (chr8:35142601-35144570). (B) An example of a very low peak intensity in **F3 ATZ-derived males** near intergenic RNA expression

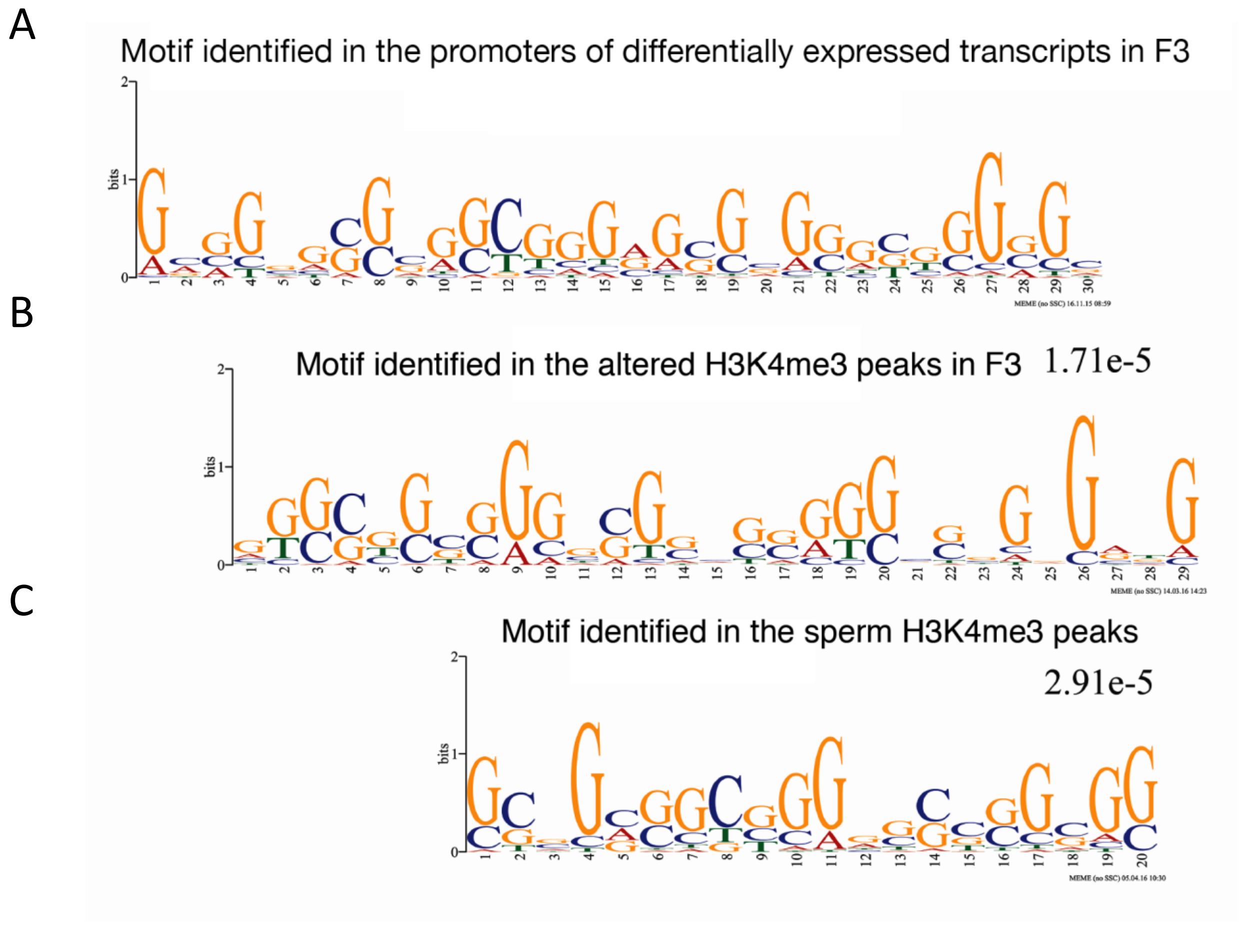

Supplementary_Figure_S26. The enriched motifs identified in the promoters of new RNA transcripts, the differential H3K4me3 peaks in F3 ATZ-derived males and the strongest peaks in sperm are similar.

(A) The motif enrichment was identified 500 bp upstream of the TSSs of transcripts with alternative TSSs in F3 ATZ-derived males. (B) Motif found in differential H3K4me3 peaks in F3 ATZ-derived males. (C) Motif found in the strongest H3K4me3 peaks identified during sperm histone retention analysis (Erkek et al. 2013). The MEME application was used as described in the Methods section, and the *q-value* of similarity between top and down motifs was estimated by TomTom.

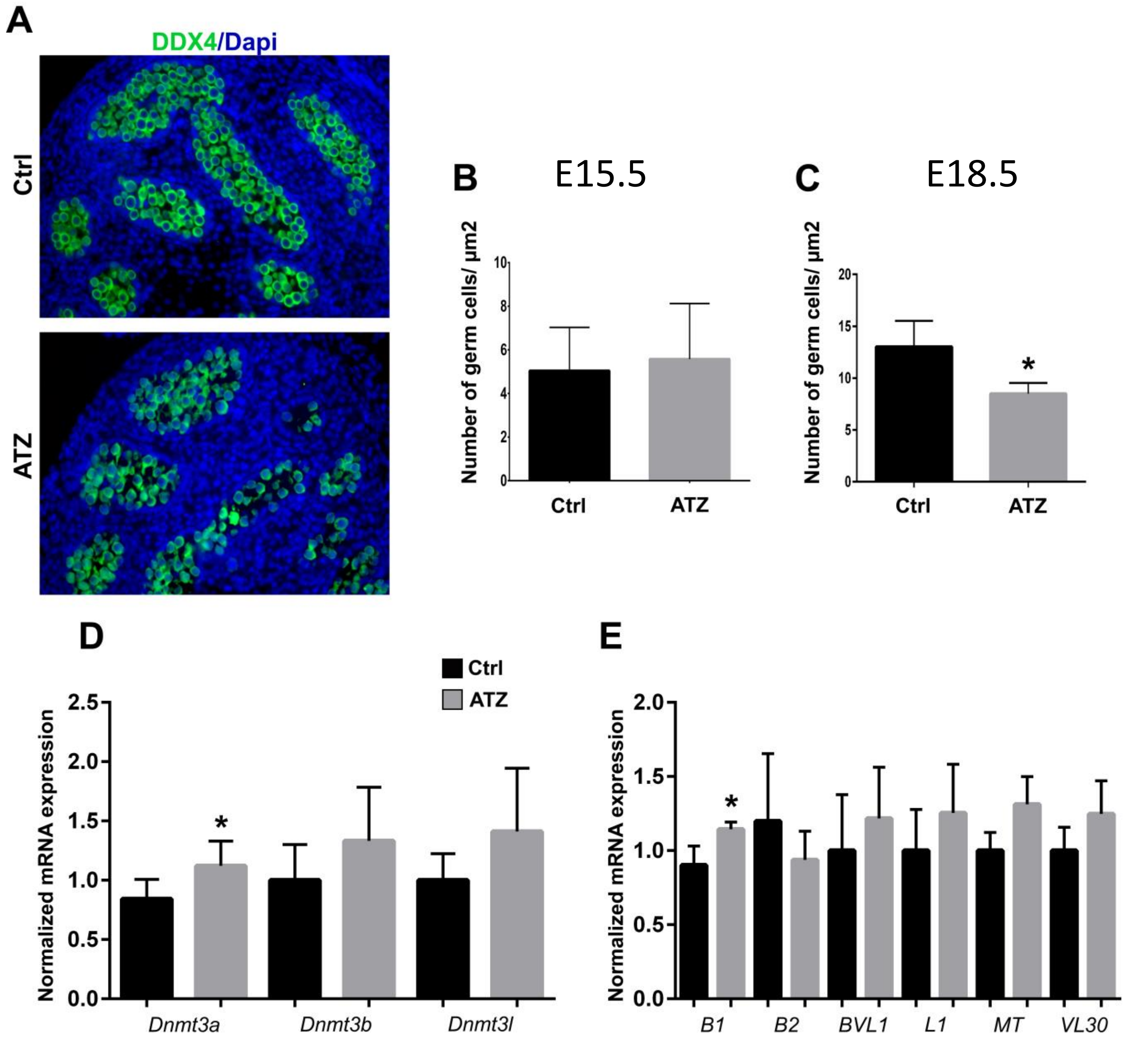

Supplementary_Figure_S27. Germ cell analysis in F1 ATZ exposed male embryos.

The testes from F1 control or ATZ exposed males were fixed with PFA and paraffin slides were prepared as described in Supplementary Methods. A) The sections were immunostained against anti- DDX4 antibody, a marker of germ cells. (B) The number of germ cells were counted in three biological replicates, B) at E15,5 and C) at E18.5. D) QPCR analysis of *de novo* DNA methyltransferases from E18.5 male gonads, total RNA was extracted as described in Material and Methods and RT PCR was performed using n=5, *, p<0.05, t-test, E) qPCR analysis of retroelements, n=5, *, p<0.05, t-test.

Pearson correlation coefficients for RNA-seq and H3K4me3 ChIP-seq samples

Pearson correlation coefficients of RNA-seq samples

A

	ATZ_1	ATZ_2	ATZ_3	CON_1	CON_2	CON_3
ATZ_1	1.00	0.98	0.96	0.94	0.85	0.97
ATZ_2	0.98	1.00	0.93	0.96	0.89	0.98
ATZ_3	0.96	0.93	1.00	0.91	0.80	0.94
CON_1	0.94	0.96	0.91	1.00	0.94	0.96
CON_2	0.85	0.89	0.80	0.94	1.00	0.85
CON_3	0.97	0.98	0.94	0.96	0.85	1.00

Pearson correlation coefficients of ChIP-seq samples

B

	ATZ_1	ATZ_2	CON_1	CON_2
ATZ_1	1.00	0.94	0.96	0.96
ATZ_2	0.94	1.00	0.93	0.92
CON_1	0.96	0.93	1.00	0.97
CON_2	0.96	0.92	0.97	1.00

Differentially expressed genes in F3 ATZ-derived testes, functionally annotated by DAVID.

Term Name	Number of genes	P Value	Genes
response to DNA damage stimulus	17	0.001	***up regulated in ATZ :*** *Cdk1, Pgap2, Ssrp1, Nbn, Usp1, Morf4l2, Xab2, Ercc8, Pnkp, Chd1l, Rif1, Bre, Fbxo6, Mus81, Bcl3, Rev3l;* ***down regulated in ATZ:*** *Mms19, Ercc8, Bre, Mus81*
nucleotide binding	71	0.006	***up regulated in ATZ:*** *Kif23, Rnasel, Gtpbp10, Aurkc, Slfn5, Mcm7, Clp1, Ndufs2, Rdm1, Cdk1, Nadsyn1, Arl9, Cdkl3, Stk4, Dapk3, Mark2, Pnkp, Glul, Hhat, Nrk, Srxn1, Rev3l, Enox1, Prps1, Abca2, Chd1l, Ddx19a, Rab11a, Nat10, Prkaa1, Mat2a, Safb2, Epha7, Tubal3, Pprc1 ;* ***down regulated in ATZ:*** *Acvrl1, Rps6kb2, Lace1, Dhx34, Nlrp4e, Pdxk, Hsp90aa1, Pik3cd, Ptbp1, Ifi47, Pkdcc, Rfc5, Rfc1, Ube2m, Aars2, Clcn3, Chkb, Hk2, Irak4, Igtp, Naip2, Ptk2b, Hnrnpd, Guf1, Rbm26, Pdk2, Chdh, Axl, Idh3b, Psmb8, Acsm3, Cirbp, Aox3, Tnk2, Cit, Katnal1*
ion binding	115	0.011	***up regulated in ATZ:*** *Rnasel, Zfp94, Plod1, Clp1, Rnf219, Luc7l2, Rps27a, Stk4, Mark2, Srxn1, Prps1, Zfp619, Nr2c2, Pxn, Wt1, Trim68, Mbnl3, B4galt4, Trpc1, Klf12, Zbtb42, Cblb, Dohh, Klf2, Cyb5r4, Kcnab1, Npnt, Zfp644, Kcnq3, P4ha3, Ndufs2, Zswim2, Dnajc24, Rev3l, Enox1, Inpp1, Ing3, Reps1, Eri2, Gatad2b, Slc39a7, Prkaa1, Cybasc3, Dtna, Mat2a, Sun1, Racgap1, Anxa4, Itpr2, Zc3h11a, Gca;* ***down regulated in ATZ :*** *Gm8935, Rabgef1, Dhx34, Zfp688, Pdxk, Polg, Nudt14, Zfp78, Nbr1, Fxyd1, Clcn3, Cacnb4, Ttc3, Irak4, Sgip1, Esrrb, Idh3b, Myl12b, Trim63, Zfp579, Frem1, Tnk2, Cryzl1, Acvrl1, Lyar, Phf23, Pcgf3, Trim6, Zfpl1, Gatad1, Kdm5b, Zcchc7, Zcchc8, Gmpr, Gpr98, Cyp27a1, Adam17, Zfpm2, Haghl, Ppm1m, Mecr, Lims2, Ecel1, Glis1, Zbtb16, Dchs1, Rnf123, Naip2, Mus81, Eno3, Scnm1, Rbm26, Adarb1, Cyp21a1, Trim28, Mid1, Capn3, Manba, Acsm3, Plscr2, Rassf1, Aox3, Cit, Scn4a*
multicellular organism reproduction	20	0.004	***up regulated in ATZ :*** *Tdrd7, Htt, Il11ra1, Racgap1, Hmga1, Wt1, Nr2c2, Calca, Gm2854, Gm2790, Prmt7, Gm14332, Gm3763, Chrna7, Spata6, Gm2964 ;* ***down regulated in ATZ*** *: Cav1, Tmbim6, Hk2, Zbtb16, Sbf1, Creb3l4, Cit*
amine biosynthetic process	7	0.007	***up regulated in ATZ :*** *Bcat1, Chdh, Glul, Mat2a, Chrna7 ;* ***down regulated in ATZ :*** *Chdh, Asl, Prodh*
mitochondrion	47	0.011	***up regulated in ATZ :*** *Bcat1, Mrps34, Rnasel, Cyb5r2, Tdrd7, Acp6, Gtpbp10, 1700021f05rik, Ndufs5, Stard4, Rab11a, Atp5h, Adck4, Ndufs2, Atpaf2, Park7, Pex11b, Glul, Mtrf1, Phb2 ;* ***down regulated in ATZ :*** *Cav1, Gcat, Hk2, Chchd3, Bnip3, Stoml2, Ccdc58, Lace1, Mrpl55, Guf1, Cox18, Gtf3c2, Sardh, Cox16, Chdh, Pdk2, Mmadhc, Polg, Fdxr, Idh3b, Acsm3, Cyp27a1, Mrpl48, Aars2, Surf1, Mecr, Prodh*
intracellular organelle lumen	40	0.022	***up regulated in ATZ :*** *Midn, Ing3, Tdrd7, Gtpbp10, Sox6, Senp5, Wt1, Blzf1, Grwd1, Gm3763, P4ha3, Nat10, Dcaf17, Rdm1, Tead2, Sun1, Carf, Med6, Trpc4ap, Pop4, Gm2964;* ***down regulated in ATZ :*** *Lyar, Bnip3, Zbtb16, Tcf7l2, Ercc8, Mus81, Mrpl55, Sardh, Olfm1, Pdk2, Polg, Trim28, Fdxr, Smad2, Acsm3, Cirbp, Pbx1, Hdac9, Aars2, Prodh*
lymphocyte activation	11	0.015	***up regulated in ATZ :*** *Cblb, Nbn, Il11ra1, Bcl3, Chrna7 ;* ***down regulated in ATZ :*** *Pik3cd, Adam17, Cacnb4, Fas, Hdac9, Ncor1*
vesicle-mediated transport	19	0.028	***up regulated in ATZ :*** *Stx3, Caly, Htt, Hook2, Blzf1, Gapvd1, Stxbp5, Exoc4, Chrna7 ;* ***down regulated in ATZ :*** *Cav1, Dennd1a, Ap4m1, Vps33b, Cd36, Stx16, Zfpl1, Rabgef1, Snap23, Cox16*
G2/M transition DNA damage checkpoint	3	0.027	***up regulated in ATZ :*** *Cdk1, Nbn ;* ***down regulated in ATZ :*** *Bre*

Differentially expressed genes in F3 ATZ-derived Liver, functionally annotated by DAVID.

Term Name	Number of genes	P -value	Genes
oxidation reduction	8	0.001	*Me1, Cyp2c44, Akr1b3, Glud1, Pgd, Cat, Dus1l, Hadha*
mitochondrion	8	0.040	*Me1, Mtrf1, Glud1, Slc25a22, Mtcp1, Cat, Timm23, Hadha*

Differentially expressed genes in F3 ATZ-derived brain

ID	Gene Name	GOTERM_BP_FAT
Pdpk1	3-phosphoinositide dependent protein kinase-1	May play a general role in signaling processes and in development
Arl16	ADP-ribosylation factor-like 16	Nucleotide binding, GTP binding, purine nucleotide binding, guanyl nucleotide binding, ribonucleotide binding
B9d1	B9 protein domain 1	Specifically or prominently expressed in mouse blastocysts compared to 4-cell stage embryos
Thumpd1	THUMP domain containing 1	acetylation, phosphoprotein,
Wwp2	WW domain containing E3 ubiquitin protein ligase 2	E3 ubiquitin-protein ligase which accepts ubiquitin from an E2 ubiquitin-conjugating enzyme in the form of a thioester and then directly transfers the ubiquitin to targeted substrates
Nnat	neuronatin	May participate in the maintenance of segment identity in the hindbrain and pituitary development
Nvl	nuclear VCP-like	Nucleotide binding, nucleoside binding, purine nucleoside binding, ATP binding
4933434E20Rik	RIKEN cDNA 4933434E20 gene	ND
Gm8399	predicted gene 8399	ND
Gm6189	predicted gene 6189	ND
Psmc3ip	proteasome (prosome, macropain) 26S subunit, ATPase 3, interacting protein	Plays an important role in meiotic recombination
Slc1a5	solute carrier family 1 (neutral amino acid transporter), member 5	Insulin-dependent transporter for L-serine and, to a lesser extent, uptake of L-alanine and L-glutamate
Tns1	tensin 1	Cell motion, cell-substrate junction assembly, cell migration, cell junction assembly, cell junction organization, cell motility, localization of cell,
Txn2	thioredoxin 2	Has an anti-apoptotic function and plays an important role in the regulation of mitochondrial membrane potential
Trim26	tripartite motif-containing 26	Zinc ion binding, ion binding, cation binding, metal ion binding, transition metal ion binding
Zbtb25	zinc finger and BTB domain containing 25	DNA binding, zinc ion binding, ion binding, cation binding, metal ion binding, transition metal ion binding,
Gm25736	predicted gene 25736	ND
Gm25475	predicted gene 25475	ND
Gm22771	predicted gene 22771	ND
Rps2-ps10	ribosomal protein S2, pseudogene 10	ND

Differentially expressed new mRNA isoforms in F3 ATZ-derived testis

extended TSS	**exon gained:** *Klf12, Gm13318, Htt, Specc1, Gatad2b, Xkr7, 9130204L05Rik, Scn4a, Gm1043, Zscan30, Zfp78, A630077J23Rik, Gm7120, 4930432E11Rik, Elp5, Slfn5, Nxnl2, Mark2, Ccdc28a, Gm13794, 4930443O20Rik, Itfg2, 1700128F08Rik, Ptk2b, Psmb8, Gm21982, Poteg, Gm13853, Gm16347, 1700026J12Rik, Hbb-bs ;* **exon number no difference:** *Scgb2b22-ps, Stard8, Prrc2b, Scgb2b23-ps, Zfp619, Pglyrp4, Lca5, 1700021L23Rik, Naip2, Plod1, Arl9, Slc19a1, Katnal1, Gm13794, Klhl15, Tex101, Fam186a, Safb2, Pkdcc, Tspan8, Mmadhc, 1110004E09Rik, Nlrp4e, Ifi27, Anxa4, 4930444M15Rik, Dennd1a, Supt20, Blzf1;* **exon loss:** *Bre, Zfp951, Igf2, Gm17359, Tubal3, Luc7l2, Prodh, Stx16, Kazald1, Zcchc8, Stag3, Nup35, Gpaa1, Clp1, Prep, 2410002F23Rik, Gm8540, Irf7, Gm21982, Gm10125, Igtp, Nadsyn1, Nudt14, Racgap1, Rgs11, Trpc1*
shortened TSS	**exon gained:** *Cyb5r2, Zc3h11a, Creb3l4, Tmem8, Srxn1, Tmem123, Gm21982, Gtf3c2, Gm7205***; exon number no difference:** *Usp25, Nbn, Gm12973, Slc35a5, Rnf219, Trim6, Prkaa1, Hhipl2, Chd1l, Gm14012, Phf23, Pcgf3, Slc6a7, Tprn, Cyp27a1, Veph1, Epm2a, 1700057H15Rik, Sox6, Adarb1, Esrrb, Kcnq3;* **exon loss:** *Ap4m1, Ssrp1, Cers5, Chmp3, Rgcc, Ifit2, Gm14012, Maged2, Myom2, Klf2, Rnasel, Cul5, Serinc2, Calca, Tmem123, Gtpbp10, Apoc2, Gm21982, Rbm48, Phyhip, Hook2, Gm8540, Bcl3, Sun1, Dcun1d4, Epb4.1l1, Vangl2, Paqr3, 9230104L09Rik, Klhl25, Ptprf, Klrb1, P4ha3, Nmrk1, Pdxk, Mecr, Vmn2r19, Hif3a, Cblb, Fam105b, Pik3cd, Unc45b, Fas, Wdr78, Serac1, Tdrd5, Slc39a12, Pxn, Akap9, Fndc1, Rftn1, Cox16, Gzmk, Col6a6, Gm13490, A530072M11Rik, Rasgrf1, Hapln1, Serinc5, Flnb, Cit, Btrc, Col4a4, Mid1, Lhfpl2, Kcnab1, Ppp1r12b, Frem1, Glis1, Hhat, Hdac9, Dtna, Gabbr2, Maml3, Nrg3*
non altered TSS	**exon gained:** *Zfp644, Serpine2, Atpaf2, Tanc1, Hsp90aa1;* **exon number no difference:** *Cwc22, Prss38, Cops3, Hap1, Inpp1, Rfc1, Gm14012;* **exon loss:** *Gm8935, E330020D12Rik, Cwc22, Rbm26, BC029214, Serp2, Dennd6b, Gm14012, Surf1, Idh3b, Flii, Trim68, Lrwd1*

Differentially expressed new mRNA isoforms in F3 ATZ-derived testis

transcripts with additional exons at 3' UTR	*1700128F08Rik,Poteg,Gm13794,Gm13318,Gm8935,Zfp644,Gm7205,Iqcj, Gm1043,E330020D12Rik,Klhl15,Gm16347,Gtf3c2,Cwc22,Igtp,Ifnar2,Kcnq3, Rbm26,2610020H08Rik,4930432E11Rik,1700128F08Rik,Calca,1700057H15 Rik,Gm13853,Gatad2b,Klf12,Ifit2,Dcun1d4,Cit,Cblb,Cblb,Col4a4,Katnal1, 9230104L09Rik,Zfp78,Gm8540,Gm8540,Gm8540,Gm14012,Safb2,Creb3l4, Zc3h11a,Trpc1,Nrg3,Mark2,Col6a6,2410002F23Rik,A630077J23Rik,Arl9, Prss38,Pxn,Zfp951,Luc7l2,Specc1,Serinc2,Ceacam3,Klrb1,Nudt14,Srxn1, Trim6,Gzmk,Mid1,Bre,Serpine2,Scgb2b23-ps,Scgb2b23-ps, Scgb2b22ps,Slc35a5,Slc35a5,Cox16,Scn4a,Slc6a7,Hif3a,BC029214,Epm2a, Naip2,Slc19a1,Hook2,Atpaf2,Kcnab1,Gpaa1,Gm21982,Anxa4,Metap1d, Metap1d,Tex22,Tmem123,Tmem123,Frem1,Prrc2b,Stx16,Pkdcc,Ssrp1,Apoc2*
shortened 3'UTR	*Maged2,Gm14012,Chmp3,Surf1,Gm7120,Irf7,Gm12973,Idh3b,Lca5,Rgs11, Nlrp4e,Flii,Trim68,Plscr2,Vmn2r19,Klhl25,Zswim2,Lrwd1,Gm21982,Ppp1r12b, Serinc5,Nup35,Gm10125,Gm17359,Ppp1r12b,Myom2*
non altered 3'UTR	*Htt,Stard8,Zfp619,Xkr7,Pglyrp4,Tubal3,Elp5,Slfn5,Kazald1,Nxnl2,Stag3, Tspan8,Prep,Psmb8,Ifi27,Gm13794,Supt20,Serp2,Tanc1,Wdr78,Igf2, Dennd1a,Esrrb,9130204L05Rik,Zscan30,1700021L23Rik,Plod1,Prodh,Ccdc2 8a,Zcchc8,4930443O20Rik,Itfg2,Tex101,Fam186a,Clp1,Mmadhc,1110004E0 9Rik,Ptk2b,Nadsyn1,1700026J12Rik,4930444M15Rik, Hbb-bs,Racgap1,Blzf1,1700057G04Rik,Gmpr,Lrrd1,Lztr1,Mtrf1,Soat2,Stoml1, Sun1,Tsr3,Wdr67,Zbtb42,Ap4m1,Usp25,Nbn,Tmem8,Prkaa1,Hhipl2,Phf23, Klf2,Pcgf3,Rnasel,Phyhip,Tprn,Cyp27a1,Sun1,Epb4.1l1,P4ha3,Nmrk1,Mecr, Pik3cd,Unc45b,Fas,Slc39a12,Akap9,Adarb1,A530072M11Rik,Rasgrf1,Hapln1 ,Flnb,Btrc,Lhfpl2,Rasgrf1,Glis1,Dtna,Gm13490,Cops3,Cwc22,Dennd6b,Hap1, Hsp90aa1,Inpp1,Rfc1,Cyb5r2,Cyb5r2,Rnf219,Cers5,Rgcc,Chd1l,Cul5, Gtpbp10,Rbm48,Bcl3,Vangl2,Paqr3,Ptprf,Pdxk,Fam105b,Serac1,Tdrd5, Veph1,Sox6,Fndc1,Rftn1,Hhat,Hdac9,Gabbr2,Maml3*

Differential transcripts with altered APA sites in F3 ATZ-derived testis

Gene	strand	Coordinates
Spin2-ps2	-	chrX:71922607-71923384
Gm11499	-	chr11:91990925-91991246
Gm6177	+	chr1:162823251-162823715
Mid1	+	chrX:166427518-166428501
Mpdz	-	chr4:80925242-80925673
Spin2-ps4	-	chrX:72135002-72135772
Spin2-ps3	+	chrX:72050138-72050899
Spin2-ps4	-	chrX:72135002-72135763
Htatsf1	+	chrX:54310068-54310383
Mid1	+	chrX:166427579-166428727
Spin2-ps3	+	chrX:72050129-72050899
Gm2500	-	chr4:42336528-42337018
Gm9969	+	chr4:42681536-42682026
Htatsf1	+	chrX:54310068-54310381
Slfn5	+	chr11:82774402-82776443
4933404K08Rik	+	chr13:23373238-23374136
Rpl21-ps8	-	chr18:82692936-82693413
Cwc22	-	chr2:77754484-77755220
Gm5687	+	chr18:13056936-13057575
Cd68	-	chr11:69477715-69478006
1700045I11Rik	+	chr4:42041568-42042058

Differential transcripts with altered APA sites in F3 ATZ-derived liver

Gene	strand	Coordinates
Gm12651	-	chr4:93570715-93571161
Gm14414	+	chr2:177244980-177245552
Gm7887	-	chr6:48474553-48474973
Gm13701	-	chr2:80817995-80818295
Rps8-ps2	-	chr2:175804078-175804765
Pop4	-	chr7:39047839-39048328
4632419I22Rik	+	chr11:86020455-86024183
Gm14438	+	chr2:175096057-175096680
Htatsf1	+	chrX:54310068-54310383
I830012O16Rik	+	chr19:34685921-34687891
Rps8-ps2	-	chr2:175804115-175804738
Htatsf1	+	chrX:54310068-54310381
Ube2j2	+	chr4:155331190-155331675
Rpl21-ps8	-	chr18:82692936-82693413
Dcaf17	+	chr2:70926404-70926701
9430015G10Rik	+	chr4:155499526-155500328
Gm14438	+	chr2:175096030-175096717
Gm14414	+	chr2:177244980-177245589

Differential expressed transcripts with altered APA sites in F3 ATZ-derived brain

Gene	strand	Coordinates
Gm14734	-	chrX:69760043-69760489
Gm11499	-	chr11:91990925-91991246
Mid1	+	chrX:166427518-166428501
Gm10320	-	chr13:99259303-99259783
Gm5528	+	chr1:72050862-72051344
Mid1	+	chrX:166427579-166428727
Gm11886	+	chr4:23349660-23349962
Tcte2	-	chr17:13682139-13683705
Gm14702	+	chrX:66223680-66224125
Gm13468	+	chr2:47472605-47473049
Gm14414	+	chr2:177244980-177245589

Functional annotation of altered F3 ATZ-derived testis H3K4me3 peaks

ID	Desc	Hyper P-value	Obs Genes	Genes
GO:0031323	regulation of cellular metabolic process	2,42E-04	188	*2410141K09Rik,2610044O15Rik8,5730507C01Rik, AW822073,Abra,Adcy1,Ankle2,App,Arfgap3, Arhgap24,Arhgap29,Arhgef19,Arid2,Asap2, Asxl2,Aym1,Bach2,Barhl2,Basp1,Bmpr2, Brd4,Camta1,Cc2d1a,Ccne1,Cdk1, Cdk5rap3,Cebpb,Cep290,Cers6,Cux1,Cux2, D10Bwg1379e,Dicer1,Dmrt1,EU599041, Edn1,Ednra,Eef2k,Efna5,Epc2,Epha2,Ern1, Etv6,Evi5,Ezh2,Fgfr1op,Fnip1,Foxc2,Foxh1, Foxk1,Galr1,Gdi2,Gm10323,Gm13157,Gm13212,Gm13225,Gm13235,Gm6712,Gm7148, Gm9376,Gm9791,Gmeb1,Gtf2e1,Gtf2e2, Gtf2h5,Gzma,Hexim2,Hipk1,Hnrnpk,Iars,Ikzf5,Il2,Il21,Ino80c,Jarid2,Jmjd1c,Klf1,Klf13,Klf2,Lamtor1,Leo1,Lhx4,Lmo1,Lpin1,Lrrtm3,Lzts1,Maf,Magi3,Map2k6,Map3k7,Mapkbp1, Med10,Mkl2,Mrpl12,Mtif3,Mtnr1a,Nfatc2ip, Nfe2l3,Nfya,Nkd2,Nr3c2,Pfdn5,Pgam5,Phf2, Phtf1,Pif1,Ppp1r3b,Ppp4r2,Prex2,Prg4,Ptpn3,Rad21,Rad23b,Ramp3,Rapgef3,Rbm26, Rbpms,Rfx8,Rgma,Rgmb,Rnf4,Rpl38,Runx1, Rxra,Sall3,Sap30l,Shox2,Siah2,Sipa1l2,Smarcal1,Smc3,Sod1,Sox14,Sox18,Sox6,Sp5, Spata24,Spdya,Sprtn,Spry1,Srsf4,Srsf7,Taf12,Tbc1d15,Tcfl5,Tgfbr3,Tgif1,Tiam1,Tnfsf8, Tnks,Tollip,Tram1,Trim24,Trim27,Tsc22d2, Tshz1,Tshz3,Uri1,Usp2,Wapal,Wnt11,Ylpm1, Ywhaq,Zbtb44,Zcchc9,Zfat,Zfhx3,Zfp191, Zfp369,Zfp36l1,Zfp462,Zfp516,Zfp521, Zfp534,Zfp536,Zfp64,Zfp640,Zfp71rs1,Zfp715,Zfp85rs1,Zfp930,Zfp932,Zfp960,Zfp97, Zfpm2,Zfyve28,Zhx2,Zmym2*

Functional annotation of altered F3 ATZ-derived testis H3K4me3 peaks

GO:0006355	regulation of transcription, DNA-dependent	2,43E-04	133	*2410141K09Rik,2610044O15Rik8,5730507C01Rik, AW822073,Abra,App,Arid2,Asxl2,Aym1, Bach2,Barhl2,Basp1,Bmpr2,Brd4,Camta1, Cc2d1a,Cdk5rap3,Cebpb,Cep290,Cers6, Cux1,Cux2,Dicer1,Dmrt1,EU599041,Edn1, Epc2,Ern1,Etv6,Ezh2,Fnip1,Foxc2,Foxh1, Foxk1,Gm10323,Gm13157,Gm13212, Gm13225,Gm13235,Gm6712,Gm7148, Gm9376,Gm9791,Gmeb1,Gtf2e1,Gtf2e2, Gtf2h5,Hexim2,Hipk1,Hnrnpk,Ikzf5,Il2, Ino80c,Jarid2,Jmjd1c,Klf1,Klf13,Klf2,Leo1, Lhx4,Lmo1,Lpin1,Maf,Map2k6,Map3k7,Mapkbp1,Med10,Mkl2,Mrpl12,Nfatc2ip,Nfe2l3,Nfya,Nr3c2,Pfdn5,Phf2,Phtf1,Rad21,Ramp3, Rbpms,Rfx8,Rgma,Rgmb,Rnf4,Runx1,Rxra,Sall3,Sap30l,Shox2,Smarcal1,Sox14, Sox18,Sox6,Sp5,Spata24,Taf12,Tcfl5, Tgfbr3,Tgif1,Tnfsf8,Tnks,Tram1,Trim24, Trim27,Tsc22d2,Tshz1,Tshz3,Uri1,Usp2, Wnt11,Ylpm1,Ywhaq,Zbtb44,Zfat,Zfhx3, Zfp191,Zfp369,Zfp462,Zfp516,Zfp521, Zfp534,Zfp536,Zfp64,Zfp640,Zfp71rs1, Zfp715,Zfp85rs1,Zfp930,Zfp932, Zfp960,Zfp97,Zfpm2,Zhx2,Zmym2*
GO:0048387	negative regulation of retinoic acid receptor signaling pathway	7,31E-04	3	*Ezh2,Tgif1,Zfp536*
GO:0032318	regulation of Ras GTPase activity	9,29E-04	16	*Arfgap3,Arhgap24,Arhgef19,Asap2,Efna5, Epha2,Evi5,Ezh2,Gdi2,Prex2,Rapgef3, Sod1,Spry1,Tbc1d15,Tiam1,Wnt11*
GO:0048318	axial mesoderm development	2,84E-03	3	*Epha2,Foxh1,Rpl38*
GO:0060119	inner ear receptor cell development	3,19E-03	5	*Cecr2,Dicer1,Gabrb3,Gpr98,Sod1*

Functional annotation of altered F3 ATZ-derived testis H3K4me3 peaks

GO:0033002	muscle cell proliferation	3,45E-03	4	*Foxc2,Naa35,Rxra,Tgfbr3*
GO:0048385	regulation of retinoic acid receptor signaling pathway	3,96E-03	3	*Ezh2,Tgif1,Zfp536*
GO:0048285	organelle fission	4,36E-03	20	*Anapc4,Ankle2,Cdc6,Cdca2,Cdk1,Cltc,Gimap3,Haus3,Kntc1,Lpin1,Map10,Nsun2,Pogz,Rad21,Sgol1,Smc3,Spc24,Tnks,Vrk1,Wapal*
GO:0031124	mRNA 3'-end processing	4,88E-03	5	*App,Cpsf6,Cstf2t,Leo1,Parn*
GO:0030004	cellular monovalent inorganic cation homeostasis	4,88E-03	5	*Cftr,Nr3c2,Rab20,Rab7,Slc26a3*
GO:0032204	regulation of telomere maintenance	5,31E-03	3	*Pif1,Tnks,Ylpm1*
GO:0001946	lymphangiogenesis	5,31E-03	3	*Bmpr2,Foxc2,Sox18*
GO:0034502	protein localization to chromosome	6,01E-03	4	*Cdk1,Rad21,Tnks,Wapal*
GO:0060491	regulation of cell projection assembly	6,52E-03	7	*Arhgap24,Bbs4,Cdc42ep4,Epha2,Kank1,Myo10,Myo3b*
GO:0016070	RNA metabolic process	6,81E-03	105	*Abra,Alyref2,App,Asxl2,Asz1,Bach2,Barhl2,Bmpr2,Brd4,Camta1,Cc2d1a,Cebpb,Cep290,Cpsf6,Cstf2t,Cux1,Cux2,Dicer1,Dido1,Dmrt1,Epc2,Ern1,Etv6,Ezh2,Fdxacb1,Foxc2,Foxh1,Foxk1,Gmeb1,Gtf2e1,Gtf2e2,Gtf2h5,Hexim2,Hipk1,Hnrnpk,Iars,Ikzf5,Ino80c,Jarid2,Jmjd1c,Klf1,Klf13,Klf2,Lcmt2,Leo1,Lhx4,Lpin1,Maf,Map2k6,Map3k7,Med10,Mkl2,Mrpl12,Nfe2l3,Nfya,Nr3c2,Nsun2,Parn,Pes1,Phf2,Phtf1,Polr3a,Pop4,Ppp4r2,Rbm26,Rbpms,Rfx8,Rnf4,Runx1,Rxra,Sall3,Sap30l,Sf3a1,Snrnp40,Sox14,Sox18,Sox6,Sp5,Spata24,Srsf4,Srsf7,Taf12,Tgif1,Tram1,Trim24,Trim27,Tshz1,Tshz3,Uri1,Utp23,Wdr83,Ylpm1,Zbtb44,Zfat,Zfhx3,Zfp191,Zfp369,Zfp36l1,Zfp516,Zfp521,Zfp536,Zfp64,Zfpm2,Zhx2,Zmym2*
GO:0036303	lymph vessel morphogenesis	6,90E-03	3	*Bmpr2,Foxc2,Sox18*
GO:0060113	inner ear receptor cell differentiation	7,52E-03	6	*Cecr2,Cux1,Dicer1,Gabrb3,Gpr98,Sod1*
GO:0050880	regulation of blood vessel size	7,69E-03	7	*Cftr,Edn1,Ednra,Foxc2,Kcnj8,Sod1,Tgfbr3*
GO:0006378	mRNA polyadenylation	8,27E-03	4	*App,Cpsf6,Cstf2t,Leo1*

Functional annotation of altered F3 ATZ-derived testis H3K4me3 peaks

GO:0035150	regulation of tube size	8,33E-03	7	*Cftr,Edn1,Ednra,Foxc2,Kcnj8,Sod1,Tgfbr3*
GO:0007067	mitosis	8,67E-03	18	*Anapc4,Ankle2,Cdc6,Cdca2,Cdk1,Cltc,Haus3,Kntc1,Map10,Nsun2,Pogz,Rad21,Sgol1,Smc3,Spc24,Tnks,Vrk1,Wapal*
GO:0090382	phagosome maturation	8,74E-03	3	*Rab20,Rab31,Rab7*
GO:0006662	glycerol ether metabolic process	8,74E-03	3	*Pdia4,Pdia5,Txndc2*
GO:0043631	RNA polyadenylation	9,58E-03	4	*App,Cpsf6,Cstf2t,Leo1*
GO:0090304	nucleic acid metabolic process	1,10E-02	124	*Abra,Alyref2,Apaf1,App,Asxl2,Asz1,Bach2,Barhl2,Bmpr2,Brd4,Camta1,Cc2d1a,Ccne1,Cdc6,Cdk1,Cebpb,Cep290,Cpsf6,Cstf2t,Cux1,Cux2,Dicer1,Dido1,Dmrt1,Dnase2a,Endou,Epc2,Ern1,Etv6,Ezh2,Fdxacb1,Foxc2,Foxh1,Foxk1,Gmeb1,Gtf2e1,Gtf2e2,Gtf2h5,Haus3,Hexim2,Hipk1,Hist1h2al,Hnrnpk,Iars,Ikzf5,Ino80c,Jarid2,Jmjd1c,Klf1,Klf13,Klf2,Lcmt2,Leo1,Lhx4,Lpin1,Maf,Map2k6,Map3k7,Med10,Mkl2,Mrpl12,Nfe2l3,Nfya,Nr3c2,Nsun2,Orc5,Parn,Pes1,Phf2,Phtf1,Pif1,Polr3a,Pop4,Ppp4r2,Rad21,Rad23b,Rbm26,Rbpms,Rdm1,Rfx8,Rnf4,Runx1,Rxra,Sall3,Sap30l,Sf3a1,Smarcal1,Smc3,Snrnp40,Sox14,Sox18,Sox6,Sp5,Spata24,Sprtn,Srsf4,Srsf7,Taf12,Tgif1,Top3a,Tram1,Trim24,Trim27,Trip13,Tshz1,Tshz3,Uri1,Utp23,Wdr83,Wrn,Ylpm1,Zbtb44,Zfat,Zfhx3,Zfp191,Zfp369,Zfp36l1,Zfp516,Zfp521,Zfp536,Zfp64,Zfpm2,Zhx2,Zmym2*
GO:0051453	regulation of intracellular pH	1,10E-02	4	*Cftr,Rab20,Rab7,Slc26a3*
GO:0030641	regulation of cellular pH	1,26E-02	4	*Cftr,Rab20,Rab7,Slc26a3*
GO:0051301	cell division	1,36E-02	23	*Anapc4,Ankle2,Bbs4,Ccne1,Cdc6,Cdc7,Cdca2,Cdk1,Dicer1,Evi5,Haus3,Kntc1,Map10,Nsun2,Pogz,Rab11fip3,Rad21,Sgol1,Smc3,Spc24,Tnks,Vrk1,Wapal*

Functional annotation of altered F3 ATZ-derived testis H3K4me3 peaks

GO:0001895	retina homeostasis	1,43E-02	4	*Abca4,Bbs4,Gpr98,Sod1*
GO:0007205	protein kinase C-activating G-protein coupled receptor signaling pathway	1,43E-02	4	*Agk,Dgkh,Edn1,Il2*
GO:0055010	ventricular cardiac muscle tissue morphogenesis	1,65E-02	5	*Foxc2,Foxh1,Rxra,Tgfbr3,Zfpm2*
GO:0055067	monovalent inorganic cation homeostasis	1,74E-02	6	*Cftr,Edn1,Nr3c2,Rab20,Rab7,Slc26a3*
GO:0015991	ATP hydrolysis coupled proton transport	1,82E-02	4	*Atp6v0e,Atp6v0e2,Atp6v1e1,Atp6v1g3*
GO:1902115	regulation of organelle assembly	1,82E-02	4	*Bbs4,Edn1,Myh4,Rnf4*
GO:0045931	positive regulation of mitotic cell cycle	1,82E-02	4	*App,Brd4,Cdk1,Usp2*
GO:0007009	plasma membrane organization	1,88E-02	8	*Ano6,Col5a1,Crb1,Nkd2,Pacsin2,Rab31,Ramp3,Sod1*
GO:0006200	ATP catabolic process	1,90E-02	10	*Abca4,Abca6,Abca9,Abcc4,Atp5j,Cftr,Dnahc7b,Mdn1,Vwa8,Wrn*
GO:0006996	organelle organization	2,00E-02	84	*4933400A11Rik,Anapc4,Ankle2,Arhgef10l,Arid2,Arl6,Bbs4,Brd4,Cdc6,Cdca2,Cdk1,Cep290,Cltc,Coro2b,Crocc,Dctn6,Dicer1,Dtnbp1,Epc2,Ezh2,Fam101b,Fam109a,Fat1,Fcho1,Fgfr1op,Gimap3,Golph3l,Gzma,Haus3,Hist1h2al,Jarid2,Jmjd1c,Kansl1,Klf1,Kntc1,Lamtor1,Leo1,Lpin1,Map10,Map3k7,Mapt,Mrpl12,Msrb2,Myh4,Nkd2,Nsun2,Nup153,Pes1,Pfn2,Phactr1,Phf2,Pif1,Pogz,Ptpn1,Rab20,Rab31,Rab7,Rad21,Rad23b,Rnf40,Rpl38,Rpsaps10,Sclt1,Sgol1,Sh2b2,Shroom1,Shroom3,Slc25a4,Smarcal1,Smc3,Sod1,Spc24,Spry1,Taf12,Tmed5,Tnks,Tomm34,Tram1,Trip13,Ttll6,Vrk1,Wapal,Wrn,Zfp462*

Functional annotation of altered F3 ATZ-derived testis H3K4me3 peaks

GO:1901360	organic cyclic compound metabolic process	2,08E-02	157	*Abca4,Abca6,Abca9,Abcc4,Abra,Adcy1, Adss,Alad,Alyref2,Amd2,Apaf1,App,Asxl2, Asz1,Atp5j,Bach2,Barhl2,Bmpr2,Brd4, Camta1,Cc2d1a,Ccne1,Cdc6,Cdk1,Cebpb, Cep290,Cftr,Cmah,Cpsf6,Cstf2t,Cux1, Cux2,Cyp51,Dctd,Dicer1,Dido1,Dio3,Dmrt1, Dnahc7b,Dnase2a,Endou,Epc2,Ern1, Etv6,Ezh2,Fdxacb1,Foxc2,Foxh1,Foxk1, Gmeb1,Gtf2e1,Gtf2e2,Gtf2h5,Haus3, Hexim2,Hipk1,Hist1h2al,Hnrnpk,Iars,Ikzf5, Ino80c,Jarid2,Jmjd1c,Klf1,Klf13,Klf2,Lcmt2, Ldhb,Leo1,Lhx4,Lpin1,Maf,Map2k6, Map3k7,Mdn1,Med10,Mkl2,Mmadhc,Mme, Mrpl12,Nfe2l3,Nfya,Nmnat3,Nr3c2,Nsun2, Nuggc,Oard1,Orc5,Parn,Pde4a,Pes1,Phf2, Phtf1,Pif1,Polr3a,Pop4,Ppcdc,Ppp4r2, Rab7,Rad21,Rad23b,Rbm26,Rbpms, Rdm1,Rfx8,Rnf4,Runx1,Rxra,Sall3,Sap30l, Sf3a1,Smarcal1,Smc3,Snrnp40,Sox14,Sox18,Sox6,Sp5,Spata24,Sprtn,Srbd1,Srp54b,Srp54c,Srsf4,Srsf7,Taf12,Tgif1,Tgm3, Top3a,Tph2,Tram1,Trim24,Trim27,Trip13, Tshz1,Tshz3,Uck2,Uck2,Uri1,Utp23,Vwa8, Wdr83,Wrn,Ylpm1,Zbtb44,Zfat,Zfhx3,Zfp191,Zfp369,Zfp36l1,Zfp516,Zfp521,Zfp536, Zfp64,Zfpm2,Zhx2,Zmym2*

Functional annotation of altered F3 ATZ-derived testis H3K4me3 peaks

GO:0031623	receptor internalization	2,26E-02	4	*Cltc,Cxcr7,Rab31,Ramp3*
GO:0009169	purine ribonucleoside monophosphate catabolic process	2,28E-02	10	*Abca4,Abca6,Abca9,Abcc4,Atp5j,Cftr, Dnahc7b,Mdn1,Vwa8,Wrn*
GO:0031123	RNA 3'-end processing	2,32E-02	5	*App,Cpsf6,Cstf2t,Leo1,Parn*
GO:0009128	purine nucleoside monophosphate catabolic process	2,38E-02	10	*Abca4,Abca6,Abca9,Abcc4,Atp5j,Cftr, Dnahc7b,Mdn1,Vwa8,Wrn*
GO:0048469	cell maturation	2,39E-02	9	*App,Cftr,Il21,Klf1,Klf2,Sclt1,Slc26a3,Sox18, Trip13*
GO:0008088	axon cargo transport	2,50E-02	4	*App,Dtnbp1,Mapt,Sod1*
GO:0009125	nucleoside monophosphate catabolic process	2,59E-02	10	*Abca4,Abca6,Abca9,Abcc4,Atp5j,Cftr, Dnahc7b,Mdn1,Vwa8,Wrn*
GO:0006898	receptor-mediated endocytosis	2,65E-02	6	*Cltc,Cxcr7,Fcho1,Pacsin2,Rab31,Ramp3*
GO:0006885	regulation of pH	2,72E-02	5	*Cftr,Edn1,Rab20,Rab7,Slc26a3*
GO:0044802	single-organism membrane organization	3,47E-02	18	*Abca4,Ankle2,Ano6,Arl6,Col5a1,Crb1, Fcho1,Nkd2,Pacsin2,Rab20,Rab31,Rab7, Ramp3,Sclt1,Sod1,Spam1,Srp54b,Srp54c*
GO:0007017	microtubule-based process	3,51E-02	21	*App,Bbs4,Ccdc39,Cltc,Crocc,Dicer1, Dnahc14,Dnahc7b,Dtnbp1,Fgfr1op,Haus3, Ift43,Kif21b,Kif5c,Map10,Mapt,Sgol1,Smc3, Sod1,Spry1,Ttll6*

Functional annotation of altered F3 ATZ-derived testis H3K4me3 peaks

ID	Desc	HyperP	Obs Genes	Genes
GO:0009161	ribonucleoside monophosphate metabolic process	3,67E-02	13	*Abca4,Abca6,Abca9,Abcc4,Adss,Atp5j, Cftr,Dnahc7b,Mdn1,Uck2,Uck2,Vwa8, Wrn*
GO:0000278	mitotic cell cycle	3,82E-02	20	*Anapc4,Ankle2,Cdc6,Cdca2,Cdk1,Cltc, Haus3,Kntc1,Map10,Nsun2,Pogz,Rad21, Sgol1,Smc3,Spc24,Spdya,Spry1,Tnks, Vrk1,Wapal*
GO:0007140	male meiosis	3,96E-02	4	*Asz1,Mei1,Spdya,Trip13*
GO:0051225	spindle assembly	3,96E-02	4	*Cltc,Dicer1,Haus3,Map10*
GO:0022411	cellular component disassembly	3,96E-02	7	*Apaf1,Arid2,Gzma,Mtif3,Mtrf1,Noxo1,Vrk1*
GO:0006873	cellular ion homeostasis	3,99E-02	16	*App,Cftr,Edn1,Galr1,Hrh4,Il2,Nr3c2, Nucb2,Rab20,Rab7,Scara5,Slc24a3, Slc26a3,Slc34a2,Sod1,Trpm1*
GO:0006821	chloride transport	4,51E-02	6	*Ano6,Cftr,Clcn3,Clic6,Gabrb3,Slc26a3*
GO:0009123	nucleoside monophosphate metabolic process	4,54E-02	13	*Abca4,Abca6,Abca9,Abcc4,Adss,Atp5j, Cftr,Dnahc7b,Mdn1,Uck2,Uck2,Vwa8, Wrn*
GO:0006457	protein folding	4,69E-02	9	*Dnajb13,Dnajb4,Fkbp14,Grpel2,Pdia5, Pfdn5,Qsox1,Txndc2,Uri1*
GO:0033044	regulation of chromosome organization	4,69E-02	8	*Asxl2,Jarid2,Lpin1,Pif1,Rnf4,Tnks,Wapal, Ylpm1*

Functional annotation of altered F1 ATZ-derived testis H3K4me3 peaks

ID	Desc	HyperP	Obs Genes	Genes
GO:0072132	mesenchyme morphogenesis	3,48E-05	5	*Hey1,Isl1,Osr1,Sox9,Twist1*
GO:0048863	stem cell differentiation	3,74E-05	15	*Cyp26a1,Eomes,Hey1,Isl1, Jarid2,Med10,Msi2,Nanos2, Nog,Osr1,Pou5f1,Sox2,Sox9, Twist1,Zhx2*
GO:0048864	stem cell development	4,26E-05	13	*Cyp26a1,Eomes,Hey1,Isl1, Med10,Msi2,Nanos2,Nog, Pou5f1,Sox2,Sox9,Twist1, Zhx2*
GO:0001706	endoderm formation	9,93E-05	5	*Eomes,Lhx1,Nog,Pou5f1,Sox2*
GO:0001714	endodermal cell fate specification	1,06E-04	3	*Eomes,Pou5f1,Sox2*
GO:0002495	antigen processing and presentation of peptide antigen via MHC class II	1,92E-04	4	*H2-Aa,H2-Ab1,H2-DMb1,March1*
GO:0003203	endocardial cushion morphogenesis	2,43E-04	4	*Hey1,Isl1,Sox9,Twist1*
GO:2000543	positive regulation of gastrulation	2,89E-04	3	*Lhx1,Osr1,Otx2*
GO:0002504	antigen processing and presentation of peptide or polysaccharide antigen via MHC class II	3,04E-04	4	*H2-Aa,H2-Ab1,H2-DMb1,March1*
GO:0072028	nephron morphogenesis	3,09E-04	5	*Hey1,Irx1,Lhx1,Osr1,Sox9*
GO:0007492	endoderm development	4,56E-04	6	*Eomes,Lhx1,Nog,Otx2,Pou5f1, Sox2*
GO:0019827	stem cell maintenance	5,27E-04	8	*Eomes,Med10,Nanos2,Nog, Pou5f1,Sox2,Sox9,Zhx2*

Functional annotation of altered F1 ATZ-derived testis H3K4me3 peaks

ID	Desc	HyperP	Obs Genes	Genes
GO:0033993	response to lipid	5,31E-04	21	*Abl2,Aicda,Alpl,Cftr,Cnr1,Cyp26a1,Il15,Isl1,Lbp,Lox,Mapk3,Nr3c2,Osr1,Pou5f1,Ptgfr,Rbp1,Slc34a2,Sox2,Sox9,Tat,Tgfbr2*
GO:0002478	antigen processing and presentation of exogenous peptide antigen	7,77E-04	4	*H2-Aa,H2-Ab1,H2-DMb1,H2-Q2*
GO:0072006	nephron development	9,58E-04	7	*Acta2,Hey1,Irx1,Lhx1,Magi2,Osr1,Sox9*
GO:0043583	ear development	1,03E-03	11	*Chd7,Grhl3,H2-DMb1,H2-Q2,Maf,Nog,Osr1,Otx2,Rpl38,Sox2,Sox9*
GO:0072073	kidney epithelium development	1,10E-03	6	*Hey1,Irx1,Lhx1,Magi2,Osr1,Sox9*
GO:0045621	positive regulation of lymphocyte differentiation	1,29E-03	6	*Cd83,H2-Aa,H2-DMb1,Ikzf1,Il15,Tgfbr2*
GO:0001708	cell fate specification	1,29E-03	6	*Eomes,Isl1,Otx2,Pou5f1,Sox2,Sox9*
GO:0042471	ear morphogenesis	1,35E-03	8	*Chd7,Grhl3,Nog,Osr1,Otx2,Rpl38,Sox2,Sox9*
GO:0001711	endodermal cell fate commitment	1,38E-03	3	*Eomes,Pou5f1,Sox2*

Functional annotation of altered F1 ATZ-derived testis H3K4me3 peaks

ID	Desc	HyperP	Obs Genes	Genes
GO:0072009	nephron epithelium development	1,41E-03	5	*Hey1,Irx1,Lhx1,Osr1,Sox9*
GO:0003197	endocardial cushion development	1,42E-03	4	*Hey1,Isl1,Sox9,Twist1*
GO:0050870	positive regulation of T cell activation	1,42E-03	8	*Cd83,Coro1a,H2-Aa,H2-Ab1,H2-DMb1,Ikzf1,Il15,Tgfbr2*
GO:0019884	antigen processing and presentation of exogenous antigen	1,62E-03	4	*H2-Aa,H2-Ab1,H2-DMb1, H2-Q2*
GO:0060009	Sertoli cell development	1,73E-03	3	*Fndc3a,Nup210l,Sox9*
GO:0072087	renal vesicle development	1,73E-03	3	*Hey1,Lhx1,Osr1*
GO:0072079	nephron tubule formation	1,73E-03	3	*Irx1,Osr1,Sox9*
GO:0072088	nephron epithelium morphogenesis	1,84E-03	4	*Irx1,Lhx1,Osr1,Sox9*
GO:0060993	kidney morphogenesis	1,87E-03	5	*Hey1,Irx1,Lhx1,Osr1,Sox9*
GO:0001649	osteoblast differentiation	2,14E-03	6	*Fignl1,Hey1,Nog,Sox2, Twist1,Wwox*
GO:0019886	antigen processing and presentation of exogenous peptide antigen via MHC class II	2,14E-03	3	*H2-Aa,H2-Ab1,H2-DMb1*
GO:0007398	ectoderm development	2,14E-03	3	*Grhl3,Lhx1,Pou5f1*
GO:0035987	endodermal cell differentiation	2,60E-03	3	*Eomes,Pou5f1,Sox2*
GO:0072210	metanephric nephron development	2,95E-03	4	*Irx1,Lhx1,Osr1,Sox9*
GO:0032526	response to retinoic acid	3,15E-03	6	*Abl2,Cyp26a1,Osr1,Pou5f1, Sox2,Sox9*

Functional annotation of altered F1 ATZ-derived testis H3K4me3 peaks

ID	Desc	HyperP	Obs Genes	Genes
GO:0071300	cellular response to retinoic acid	3,28E-03	4	*Abl2,Cyp26a1,Osr1,Sox9*
GO:0045582	positive regulation of T cell differentiation	3,60E-03	5	*Cd83,H2-Aa,H2-DMb1,Ikzf1,Tgfbr2*
GO:0070849	response to epidermal growth factor stimulus	3,69E-03	3	*Garem,Mapk3,Sox9*
GO:0045619	regulation of lymphocyte differentiation	3,82E-03	7	*Cd83,H2-Aa,H2-DMb1,Ikzf1,Il15,Ndfip1,Tgfbr2*
GO:0010033	response to organic substance	4,02E-03	43	*Abl2,Aicda,Alpl,Cd83,Cftr, Cnr1,Coro1a,Cyp26a1,Fut8, Garem,Gm13305,H2-Aa, H2Ab1,Idi1,Ifnlr1,Il11ra2,Il15, Isl1,Jak1,Klf6,Lbp,Lhx1,Lox, Magi2,Mapk3,Nog,Nos1, Nr3c2,Nub1,Osr1,Pdgfd, Pou5f1,Ptgfr,Rad51,Rbp1, Slc34a2,Snrpn,Sox2,Sox9, Tat,Tgfbr2,Tmbim6,Wwox*
GO:0048002	antigen processing and presentation of peptide antigen	4,17E-03	5	*H2-Aa,H2-Ab1,H2-DMb1, H2-Q2,March1*
GO:0045580	regulation of T cell differentiation	4,49E-03	6	*Cd83,H2-Aa,H2-DMb1,Ikzf1,Il15,Tgfbr2*
GO:0060008	Sertoli cell differentiation	5,02E-03	3	*Fndc3a,Nup210l,Sox9*
GO:0001503	ossification	5,28E-03	9	*Alpl,Fignl1,Hey1,Nog,Rpl38, Sox2,Sox9,Twist1,Wwox*

Functional annotation of altered F1 ATZ-derived testis H3K4me3 peaks

ID	Desc	HyperP	Obs Genes	Genes
GO:0046320	regulation of fatty acid oxidation	5,78E-03	3	*Cnr1,Ppargc1a,Twist1*
GO:0090190	positive regulation of branching involved in ureteric bud morphogenesis	5,78E-03	3	*Lhx1,Nog,Sox9*
GO:0072078	nephron tubule morphogenesis	5,78E-03	3	*Irx1,Osr1,Sox9*
GO:0050863	regulation of T cell activation	6,27E-03	9	*Cd83,Coro1a,H2-Aa,H2-Ab1,H2-DMb1,Ikzf1,Il15,Ndfip1,Tgfbr2*
GO:0060042	retina morphogenesis in camera-type eye	6,37E-03	4	*Lhx1,Pvrl3,Sox2,Sox9*
GO:0034502	protein localization to chromosome	6,61E-03	3	*Casc5,Lrwd1,Tnks*
GO:2000826	regulation of heart morphogenesis	6,61E-03	3	*Isl1,Sox9,Twist1*
GO:0007369	gastrulation	6,63E-03	7	*Eomes,Lhx1,Nog,Otx2, Pou5f1,Sox2,Tgfbr2*
GO:0002696	positive regulation of leukocyte activation	6,94E-03	9	*Cd83,Coro1a,H2-Aa,H2-Ab1,H2DMb1,Ikzf1,Il15,Lbp, Tgfbr2*
GO:2000779	regulation of double-strand break repair	7,50E-03	3	*Fignl1,Rad51,Twist1*
GO:0009072	aromatic amino acid family metabolic process	7,50E-03	3	*Dct,Tat,Tph1*
GO:0001837	epithelial to mesenchymal transition	7,52E-03	4	*Eomes,Hey1,Nog,Sox9*
GO:0035019	somatic stem cell maintenance	7,52E-03	4	*Nog,Sox2,Sox9,Zhx2*
GO:0042474	middle ear morphogenesis	8,46E-03	3	*Nog,Osr1,Rpl38*
GO:0060795	cell fate commitment involved in formation of primary germ layer	8,46E-03	3	*Eomes,Pou5f1,Sox2*

Supplementary_Table_ S8

Functional annotation of altered F1 ATZ-derived testis H3K4me3 peaks

ID	Desc	HyperP	Obs Genes	Genes
GO:0090189	regulation of branching involved in ureteric bud morphogenesis	8,46E-03	3	*Lhx1,Nog,Sox9*
GO:0046635	positive regulation of alpha-beta T cell activation	8,79E-03	4	*Cd83,H2-Ab1,Ikzf1,Tgfbr2*
GO:0090183	regulation of kidney development	9,48E-03	4	*Lhx1,Nog,Osr1,Sox9*
GO:0002066	columnar/cuboidal epithelial cell development	9,49E-03	3	*Insm1,Klf5,Spdef*
GO:0061333	renal tubule morphogenesis	9,49E-03	3	*Irx1,Osr1,Sox9*
GO:0002064	epithelial cell development	9,94E-03	10	*Acta2,Capza3,Cftr,Fndc3a,Insm1,Klf5,Magi2,Nup210l,Sox9,Spdef*
GO:0006282	regulation of DNA repair	1,10E-02	4	*Bre,Fignl1,Rad51,Twist1*
GO:2001252	positive regulation of chromosome organization	1,17E-02	4	*Jarid2,Mapk3,Nos1,Tnks*
GO:0035065	regulation of histone acetylation	1,30E-02	3	*Mapk3,Nos1,Twist1*

Differentially expressed genes in F3 ATZ-derived testis compared to control, containing the binding sites of Sp1/Sp3/Sp4 and Wt1 sites with more than >70% conservative score in mammalian species

**SP4**	**SP3**	**SP1**	**WT1**
Zfp644	*Cul5*	*Cul5*	*Cul5*
Cul5	*Zfp644*	*Zfp644*	*Zfp644*
Ppp1r12b	*Maged2*	*Ppp1r12b*	*Safb2*
Hsp90aa1	*Ppp1r12b*	*Hsp90aa1*	*Dennd1a*
Dennd1a	*Dennd1a*	*Maged2*	*Maged2*
Surf1	*Hsp90aa1*	*Dennd1a*	*Hsp90aa1*
Pik3cd	*Pik3cd*	*Mark2*	*Flii*
Maged2	*Rbm26*	*Flii*	*Mark2*
Cops3	*Flii*	*Pik3cd*	*Gm10125*
Rbm26	*Atpaf2*	*Atpaf2*	*Lrwd1*
Flii	*Mark2*	*Rbm26*	*Atpaf2*
Irf7	*Safb2*	*Safb2*	*Clp1*
Atpaf2	*Cops3*	*Cops3*	*Slc35a5*
Serpine2	*Itfg2*	*Anxa4*	*Itfg2*
BC029214	*BC029214*	*Tex101*	*Anxa4*
Gpaa1	*Anxa4*	*Ssrp1*	*Stard8*
Ssrp1	*Ssrp1*	*Tanc1*	*Usp25*
Usp25	*Usp25*	*Pkdcc*	*Tanc1*
Phf23	*Pkdcc*	*Phf23*	*Pkdcc*
Pkdcc	*Stag3*	*Prrc2b*	*Phf23*
Tanc1	*Tanc1*	*Stx16*	*Stx16*
Luc7l2	*Phf23*	*Klhl15*	*Stag3*
Stx16	*Klhl15*	*Stag3*	*Arl9*

Differentially expressed genes in F3 ATZ-derived testis, containing the binding sites of Sp1/Sp3/Sp4 and Wt1 sites with more than >70% conservative score in mammalian species

SP4	SP3	SP1	WT1
Stag3	*Prrc2b*	*Ap4m1*	*Metap1d*
Klhl15	*Stx16*	*Sun1*	*Prep*
Prrc2b	*Sun1*	*Ptprf*	*Chmp3*
Sun1	*Luc7l2*	*Stoml1*	*Zbtb42*
Dcun1d4	*Prep*	*Hif3a*	*Klhl15*
Prep	*Ptprf*	*Tmem8*	*Stoml1*
Hook2	*Stoml1*	*Zc3h11a*	*Trpc1*
Stoml1	*Hif3a*	*Trpc1*	*Zc3h11a*
Ptprf	*Tmem8*	*Serpine2*	*Tmem8*
Zc3h11a	*Trpc1*	*Cers5*	*Tspan8*
Tmem8	*Zc3h11a*	*Tsr3*	*Nudt14*
Flnb	*Serpine2*	*Zswim2*	*Serpine2*
Trpc1	*Rgcc*		*Ptprf*
Nbn	*Zswim2*		
Wdr67	*Prkaa1*		
Prkaa1			
Cers5			
Tsr3			
Rgcc			
Gm21982			

Supplementary_Table_S 10

The altered H3K4me3 peaks near the genes, containing Sp1/Sp3/Sp4 and Wt1 binding sites sites with more than >70% conservative score in mammalian species

**SP4**	**SP3**	**SP1**	**WT1**
Dhx40	*Fgf18*	*Fgf18*	*Fgf18*
Fgf18	*Pabpc1l*	*C030017K20Rik*	*Trim27*
C030017K20Rik	*C030017K20Rik*	*Dnajb4*	*Scube2*
Irak2	*Dnajb4*	*Tmem158*	*Irak2*
Scube2	*Scube2*	*Scube2*	*Rapgef3*
1700030C10Rik	*Tmem158*	*3110053B16Rik*	*Gm16257*
Tmem158	*Rapgef3*	*Dhx40*	*3110053B16Rik*
Trim27	*Gm16257*	*Zfat*	*Zfp36l1*
3110053B16Rik	*3110053B16Rik*	*Gm14290*	*Gm17591*
Zfp36l1	*Zfat*	*Etv6*	*Gm14290*
Gm17591	*Dhx40*	*Rapgef3*	*Tmem158*
Zfat	*4933411K20Rik*	*Gm16257*	*Alad*
Ogfr	*Gabrb3*	*4933411K20Rik*	*Gabrb3*
Rapgef3	*Zhx2*	*Pabpc1l*	*Zhx2*
Gm16257	*Cux1*	*Gabrb3*	*Cux1*
Gabrb3	*Gm16599*	*Zhx2*	*Gm16599*
Zhx2	*Gpr27*	*Cux1*	*Zbtb44*
Cux1	*Tmem52*	*Gm16599*	*Pde4a*
Gm16599	*Kansl1*	*Kansl1*	*Arrdc3*
Kif21b	*1700081L11Rik*	*1700081L11Rik*	*1700023H06Rik*
Col9a3	*Sox18*	*Tmem52*	*Pole3*
Arrdc3	*1700030C10Rik*	*Sox18*	*Gpr27*
1700023H06Rik	*Gm16256*	*1700030C10Rik*	*Sox18*
Gpr27	*Tram1*	*Anks1b*	*Tatdn2*
Tmem52	*Anks1b*	*Tram1*	*Anks1b*
Tatdn2	*Siva1*	*Kcnb1*	*Gm16256*
Sox18	*8030451A03Rik*	*Gm16256*	*1700030C10Rik*
Kansl1	*Tnc*	*8030451A03Rik*	*Tram1*
1700081L11Rik	*Ldlrad4*	*Tnc*	*Kcnb1*
Mei1	*Pdia5*	*Pdia5*	*Sdk2*
Tram1	*Kcnb1*	*Sdk2*	*Pdia5*
Ldlrad4	*Sdk2*	*Crocc*	*Crocc*
Cntnap2	*1600014C10Rik*		
Anks1b			
Kcnb1			
Pdia5			
Gm16256			
1600014C10Rik			
Crocc			
Akr1e1			

Supplementary Table S11

Genes located near F3 testis differential peaks and retained in human sperm nucleosomes

Official_gene_symbol	Name
Kcnj16	potassium inwardly-rectifying channel, subfamily J, member 16
Prg4	proteoglycan 4 (megakaryocyte stimulating factor, articular superficial zone protein)
Slc16a1	solute carrier family 16 (monocarboxylic acid transporters), member 1
Fgf18	fibroblast growth factor 18
Mtnr1a	melatonin receptor 1A
Ms4a5	membrane-spanning 4-domains, subfamily A, member 5
Zcchc7	zinc finger, CCHC domain containing 7
Dtnbp1	dystrobrevin binding protein 1
Acss3	acyl-CoA synthetase short-chain family member 3
Rasgef1b	RasGEF domain family, member 1B; hypothetical protein LOC100044232
Cdkn3	cyclin-dependent kinase inhibitor 3
A2m	alpha-2-macroglobulin
Tmed5	transmembrane emp24 protein transport domain containing 5
Rab31	RAB31, member RAS oncogene family
Nkain3	Na+/K+ transporting ATPase interacting 3
Tram1l1	translocation associated membrane protein 1-like 1
Ppcdc	phosphopantothenoylcysteine decarboxylase
Arrdc4	arrestin domain containing 4
Cog7	component of oligomeric golgi complex 7
Arl6	ADP-ribosylation factor-like 6
Tph2	tryptophan hydroxylase 2
Rgmb	RGM domain family, member B
Prss48	predicted gene 1019
Itga11	integrin alpha 11
Cpm	carboxypeptidase M
Gpx8	glutathione peroxidase 8 (putative)
Xylt1	xylosyltransferase 1
Rfx8	RIKEN cDNA 4933400N17 gene
Cux2	cut-like homeobox 2
Cftr	cystic fibrosis transmembrane conductance regulator homolog
Qsox1	quiescin Q6 sulfhydryl oxidase 1
Grhpr	glyoxylate reductase/hydroxypyruvate reductase
Arl4d	ADP-ribosylation factor-like 4D; hypothetical protein LOC100044157
Nr3c2	nuclear receptor subfamily 3, group C, member 2
Dkk2	dickkopf homolog 2 (Xenopus laevis)
Fchsd2	FCH and double SH3 domains 2
Med10	mediator of RNA polymerase II transcription, subunit 10 homolog (NUT2, S. cerevisiae)
Tmem202	transmembrane protein 202
Hebp2	heme binding protein 2
Tmco1	transmembrane and coiled-coil domains 1
Evi5	ecotropic viral integration site 5
Slc25a4	solute carrier family 25 (mitochondrial carrier, adenine nucleotide translocator), member 4
Stk33	serine/threonine kinase 33
Gzma	granzyme A
Nkain1	Na+/K+ transporting ATPase interacting 1
Kank1	KN motif and ankyrin repeat domains 1
Fgl2	fibrinogen-like protein 2
Il1r2	interleukin 1 receptor, type II
Phtf1	putative homeodomain transcription factor 1
Oxr1	oxidation resistance 1
Tmem200b	transmembrane protein 200B
Abcc4	ATP-binding cassette, sub-family C (CFTR/MRP), member 4
Cgnl1	cingulin-like 1
Ldhb	lactate dehydrogenase B; predicted gene 5514
Epc2	enhancer of polycomb homolog 2
Ywhaq	tyrosine 3-monooxygenase/tryptophan 5-monooxygenase activation protein, theta polypeptide
Arhgap29	Rho GTPase activating protein 29
Pla2g4a	phospholipase A2, group IVA (cytosolic, calcium-dependent)
Gpx5	glutathione peroxidase 5
Zfp36l1	zinc finger protein 36, C3H type-like 1
Als2cr12	amyotrophic lateral sclerosis 2 (juvenile) chromosome region, candidate 12 (human)
Veph1	ventricular zone expressed PH domain homolog 1 (zebrafish)
Cdc6	cell division cycle 6 homolog
Tnc	tenascin C
Hmcn1	hemicentin 1
Sox6	SRY-box containing gene 6

Slc24a3	solute carrier family 24 (sodium/potassium/calcium exchanger), member 3
Myo3b	myosin IIIB
Gtf2e1	general transcription factor II E, polypeptide 1 (alpha subunit)
Mdn1	midasin homolog (yeast)
Slc38a9	solute carrier family 38, member 9
Sgol1	shugoshin-like 1 (S. pombe)
Mapkbp1	mitogen-activated protein kinase binding protein 1
Pdzrn3	PDZ domain containing RING finger 3
Col4a1	collagen, type IV, alpha 1
Tnks	tankyrase, TRF1-interacting ankyrin-related ADP-ribose polymerase
Lmo1	LIM domain only 1
Fndc3a	fibronectin type III domain containing 3A
Cpsf6	cleavage and polyadenylation specific factor 6
Wnt11	wingless-related MMTV integration site 11
Frmd4b	FERM domain containing 4B
Sgk1	serum/glucocorticoid regulated kinase 1
Fam109a	family with sequence similarity 109, member A
Abca9	ATP-binding cassette, sub-family A (ABC1), member 9
Fscb	fibrous sheath CABYR binding protein
Mme	membrane metallo endopeptidase
Tnfaip8	tumor necrosis factor, alpha-induced protein 8
Nfe2l3	nuclear factor, erythroid derived 2, like 3
Fam65b	family with sequence similarity 65, member B
Zfat	zinc finger and AT hook domain containing
Actr6	ARP6 actin-related protein 6 homolog (yeast)
Mast4	microtubule associated serine/threonine kinase family member 4
Dynlrb2	dynein light chain roadblock-type 2
Maf	avian musculoaponeurotic fibrosarcoma oncogene homolog
Ergic1	endoplasmic reticulum-golgi intermediate compartment (ERGIC) 1
Nat8l	N-acetyltransferase 8-like
Lhfpl2	lipoma HMGIC fusion partner-like 2
Phactr1	phosphatase and actin regulator 1
Afap1l1	actin filament associated protein 1-like 1
Dicer1	Dicer1, Dcr-1 homolog (Drosophila)
Isca1	iron-sulfur cluster assembly 1 homolog (S. cerevisiae)
Psmd7	proteasome (prosome, macropain) 26S subunit, non-ATPase, 7
Tmem158	transmembrane protein 158

Rasef	RAS and EF hand domain containing
Tiam1	T-cell lymphoma invasion and metastasis 1
Zfp64	zinc finger protein 64
Klkb1	kallikrein B, plasma 1
Tgif1	TGFB-induced factor homeobox 1
Abra	actin-binding Rho activating protein
Tulp4	tubby like protein 4
Txndc2	thioredoxin domain containing 2 (spermatozoa)
Dirc2	disrupted in renal carcinoma 2 (human)
Alcam	activated leukocyte cell adhesion molecule
Pdzrn4	PDZ domain containing RING finger 4
Galm	galactose mutarotase
Sema5b	sema domain, seven thrombospondin repeats (type 1 and type 1-like), transmembrane domain (TM) and short cytoplasmic domain, (semaphorin) 5B
Zc3h13	zinc finger CCCH type containing 13
Tmem144	transmembrane protein 144
Dlgap1	discs, large (Drosophila) homolog-associated protein 1
Bcl2l14	BCL2-like 14 (apoptosis facilitator)
Lphn3	latrophilin 3
Mtif3	mitochondrial translational initiation factor 3
Cd180	CD180 antigen
Abca4	ATP-binding cassette, sub-family A (ABC1), member 4
Cabin1	calcineurin binding protein 1
Fnip1	folliculin interacting protein 1
Spin1	spindlin 1
Slc26a3	solute carrier family 26, member 3
Edn1	endothelin 1
Mmadhc	methylmalonic aciduria (cobalamin deficiency) cblD type, with homocystinuria
Mkl2	MKL/myocardin-like 2
Siah3	seven in absentia homolog 3 (Drosophila)
Pdia4	protein disulfide isomerase associated 4
Barhl2	BarH-like 2 (Drosophila)
Atp10a	ATPase, class V, type 10A
Foxk1	forkhead box K1
Cops8	COP9 (constitutive photomorphogenic) homolog, subunit 8 (Arabidopsis thaliana)
Parn	poly(A)-specific ribonuclease (deadenylation nuclease)
Kif21b	kinesin family member 21B
Hrh4	histamine receptor H4
Rnd3	Rho family GTPase 3
Arrdc3	arrestin domain containing 3

Lmbr1	limb region 1
Lpin1	lipin 1
Galnt10	UDP-N-acetyl-alpha-D-galactosamine:polypeptide N-acetylgalactosaminyltransferase 10
Mak16	MAK16 homolog (S. cerevisiae)
App	amyloid beta (A4) precursor protein
Vrk1	vaccinia related kinase 1
Kcnj8	potassium inwardly-rectifying channel, subfamily J, member 8
Utp23	UTP23, small subunit (SSU) processome component, homolog (yeast)
Basp1	brain abundant, membrane attached signal protein 1
Tram1	translocating chain-associating membrane protein 1
Zhx2	zinc fingers and homeoboxes 2
Fam120a	family with sequence similarity 120, member A
Wdfy3	WD repeat and FYVE domain containing 3
Arid2	AT rich interactive domain 2 (ARID, RFX-like); RIKEN cDNA 1700124K17 gene
N6amt1	N-6 adenine-specific DNA methyltransferase 1 (putative)
Ramp3	receptor (calcitonin) activity modifying protein 3
Map3k7	mitogen-activated protein kinase kinase kinase 7; predicted gene 8188
Ylpm1	YLP motif containing 1
Grid1	glutamate receptor, ionotropic, delta 1
Dcn	decorin
Sap30l	SAP30-like
Mapt	microtubule-associated protein tau
Myh4	myosin, heavy polypeptide 4, skeletal muscle
Trafd1	TRAF type zinc finger domain containing 1
Cul1	cullin 1
Ptplad2	protein tyrosine phosphatase-like A domain containing 2
Ndst3	N-deacetylase/N-sulfotransferase (heparan glucosaminyl) 3
Cstf2t	cleavage stimulation factor, 3' pre-RNA subunit 2, tau
Pak6	p21 protein (Cdc42/Rac)-activated kinase 6
Dennd1b	DENN/MADD domain containing 1B
Prkrir	protein-kinase, interferon-inducible double stranded RNA dependent inhibitor, repressor of (P58 repressor)
Dmrt1	doublesex and mab-3 related transcription factor 1

Spam1	sperm adhesion molecule 1
Crb1	crumbs homolog 1 (Drosophila)
Gxylt1	glycosyltransferase 8 domain containing 3
Runx1	runt related transcription factor 1
Wapal	wings apart-like homolog (Drosophila)
Gpr98	G protein-coupled receptor 98
Adcy1	adenylate cyclase 1
Cdca2	cell division cycle associated 2
Asap2	development and differentiation enhancing factor 2
Ano6	anoctamin 6
Nek11	NIMA (never in mitosis gene a)-related expressed kinase 11
Cdc7	cell division cycle 7 (S. cerevisiae)
Shroom3	shroom family member 3
Spry1	sprouty homolog 1 (Drosophila
Scamp1	secretory carrier membrane protein 1
Ednra	endothelin receptor type A
Ccdc39	coiled-coil domain containing 39
Trhde	TRH-degrading enzyme
Phf2	PHD finger protein 2
Anks1b	ankyrin repeat and sterile alpha motif domain containing 1B
Efna5	ephrin A5
Stxbp5l	syntaxin binding protein 5-like
Kif5c	kinesin family member 5C
Sall3	sal-like 3 (Drosophila)
Ptpn3	protein tyrosine phosphatase, non-receptor type 3
Bmpr2	bone morphogenic protein receptor, type II (serine/threonine kinase)
Lzts1	leucine zipper, putative tumor suppressor 1
Rnf38	ring finger protein 38
Otud6b	OTU domain containing 6B; predicted gene 12444
F11	coagulation factor XI
Atp6v1g3	ATPase, H+ transporting, lysosomal V1 subunit G3
Dgkh	diacylglycerol kinase, eta
Lhx4	LIM homeobox protein 4
Rpl38	ribosomal protein L38
Ino80c	INO80 complex subunit C
Atp5j	ATP synthase, H+ transporting, mitochondrial F0 complex, subunit F pseudogene
Arid1b	AT rich interactive domain 1B (SWI-like)
Kcnk2	potassium channel, subfamily K, member 2
Galr1	galanin receptor 1

Trpc7	transient receptor potential cation channel, subfamily C, member 7
Pdia5	protein disulfide isomerase associated 5
Mctp2	multiple C2 domains, transmembrane 2
Dio3	deiodinase, iodothyronine type III
Exoc8	exocyst complex component 8
Rps29	ribosomal protein S29
Sdk2	sidekick homolog 2 (chicken)
Zbtb40	zinc finger and BTB domain containing 40
Ppp4r2	protein phosphatase 4, regulatory subunit 2
Nek7	NIMA (never in mitosis gene a)-related expressed kinase 7
Bach2	BTB and CNC homology 2
Sclt1	sodium channel and clathrin linker 1
Zfpm2	zinc finger protein, multitype 2
Rad23b	RAD23b homolog (S. cerevisiae)
Anapc4	anaphase promoting complex subunit 4
Chchd2	coiled-coil-helix-coiled-coil-helix domain containing 2
Chsy3	chondroitin sulfate synthase 3
Abca6	ATP-binding cassette, sub-family A (ABC1), member 6
Zfhx3	zinc finger homeobox 3
Apaf1	apoptotic peptidase activating factor 1
Trim24	tripartite motif-containing 24
Zmym2	zinc finger, MYM-type 2
Cep170	centrosomal protein 170
Siah2	seven in absentia 2
Sox14	SRY-box containing gene 14
Fam198b	RIKEN cDNA 1110032E23 gene
Cdh10	cadherin 10
Tnfsf8	tumor necrosis factor (ligand) superfamily, member 8
Cbll1	Casitas B-lineage lymphoma-like 1
Kctd3	predicted gene 7553; potassium channel tetramerisation domain containing 3
Pgk2	phosphoglycerate kinase 2
Magi3	membrane associated guanylate kinase, WW and PDZ domain containing 3
Scrn1	secernin 1
Dctd	dCMP deaminase
Crocc	ciliary rootlet coiled-coil, rootletin
Clic6	chloride intracellular channel 6
Sp5	trans-acting transcription factor 5
Camta1	calmodulin binding transcription activator 1
Muc5b	mucin 5, subtype B, tracheobronchial
Il20rb	interleukin 20 receptor beta
Gtf2h5	general transcription factor IIH, polypeptide 5
Msrb2	methionine sulfoxide reductase B2
Smpdl3a	sphingomyelin phosphodiesterase, acid-like 3A
Nmnat3	nicotinamide nucleotide adenylyltransferase 3
Cdk1	cell division cycle 2 homolog A (S. pombe)
Stk35	serine/threonine kinase 35
Asxl2	additional sex combs like 2 (Drosophila)
Tollip	toll interacting protein
Tmem66	transmembrane protein 66
Arglu1	arginine and glutamate rich 1
Akap9	A kinase (PRKA) anchor protein (yotiao) 9
Rnf32	ring finger protein 32
Gpr149	G protein-coupled receptor 149
Dscr3	Down syndrome critical region gene 3
Zbtb44	zinc finger and BTB domain containing 44
Adss	adenylosuccinate synthetase, non muscle
Rad21	RAD21 homolog (S. pombe)
Rgma	RGM domain family, member A
Agk	predicted gene 8546; acylglycerol kinase
Klf13	Kruppel-like factor 13
Gdi2	predicted gene 4973; guanosine diphosphate (GDP) dissociation inhibitor 2
Nucb2	nucleobindin 2
Edil3	EGF-like repeats and discoidin I-like domains 3
Nup153	nucleoporin 153
Tshz3	teashirt zinc finger family member 3
Mtrf1	mitochondrial translational release factor 1
Atg16l2	autophagy related 16 like 2 (S. cerevisiae)
Prex2	phosphatidylinositol-3,4,5-trisphosphate-dependent Rac exchange factor 2
Map2k6	mitogen-activated protein kinase kinase 6
Col6a3	collagen, type VI, alpha 3
Trpm1	transient receptor potential cation channel, subfamily M, member 1
Uck2	uridine-cytidine kinase 2
Vps52	vacuolar protein sorting 52 (yeast)
Clybl	citrate lyase beta like
Sipa1l2	hypothetical protein LOC676463; signal-induced proliferation-associated 1 like 2
Sod1	superoxide dismutase 1, soluble
Ezh2	enhancer of zeste homolog 2 (Drosophila)
Ppm1l	protein phosphatase 1 (formerly 2C)-like
Cep290	centrosomal protein 290
Tsc22d2	TSC22 domain family, member 2
Gpc6	glypican 6
Rbm26	RNA binding motif protein 26

Spock1	sparc/osteonectin, cwcv and kazal-like domains proteoglycan 1
Il2	interleukin 2
Pld5	phospholipase D family, member 5
Jmjd1c	jumonji domain containing 1C
Clcn3	chloride channel 3
Shroom1	shroom family member 1
Btg1	B-cell translocation gene 1, anti-proliferative
Gabrb3	gamma-aminobutyric acid (GABA) A receptor, subunit beta 3
Arih1	ariadne ubiquitin-conjugating enzyme E2 binding protein homolog 1 (Drosophila)
Sema3c	sema domain, immunoglobulin domain (Ig), short basic domain, secreted, (semaphorin) 3C
Shox2	short stature homeobox 2
Lrrtm3	leucine rich repeat transmembrane neuronal 3
Coro2b	coronin, actin binding protein, 2B
Wrn	Werner syndrome homolog
Ivns1abp	influenza virus NS1A binding protein
Tgfbr3	transforming growth factor, beta receptor III
Asz1	ankyrin repeat, SAM and basic leucine zipper domain containing 1
Ccdc62	coiled-coil domain containing 62
Serp2	stress-associated endoplasmic reticulum protein family member 2
Kpna4	karyopherin (importin) alpha 4
Hs3st5	heparan sulfate (glucosamine) 3-O-sulfotransferase 5
Arhgap24	Rho GTPase activating protein 24
Fat1	FAT tumor suppressor homolog 1 (Drosophila)
Ggh	gamma-glutamyl hydrolase
Ppfia2	protein tyrosine phosphatase, receptor type, f polypeptide (PTPRF), interacting protein (liprin), alpha 2
Ube2e2	ubiquitin-conjugating enzyme E2E 2 (UBC4/5 homolog, yeast)
Srbd1	S1 RNA binding domain 1
Pfn2	profilin 2
Zcchc9	zinc finger, CCHC domain containing 9
Sdc3	syndecan 3
Lmod3	leiomodin 3 (fetal)
Kansl1	KAT8 regulatory NSL complex subunit 1
Map10	microtubule-associated protein 10
Asic2	acid-sensing (proton-gated) ion channel 2
Cers6	ceramide synthase 6
Vwa8	von Willebrand factor A domain containing 8

Tmem178b	transmembrane protein 178
Caap1	caspase activity and apoptosis inhibitor 1
Tenm3	teneurin transmembrane protein 3
Focad	focadhesin
Saysd1	SAYSVFN motif domain containing 1
Scp2d1	SCP2 sterol-binding domain containing 1
Tmem242	transmembrane protein 242
Gsap	gamma-secretase activating protein
Ldlrad4	low density lipoprotein receptor class A domain containing 4
Uri1	URI1, prefoldin-like chaperone

Common regions affected in vinclozolin transgenerational and this studies

Official symbol	**Official Full Name**	**Function of the protein**	**FC, in our data**
Cwc22	CWC22 spliceosome-associated protein	Plays role in mRNA splicing	3.21
Slmo2	slowmo homolog 2 (Drosophila)	The protein has the phosphatidic acid transporter activity	2.34
Lrrc61	leucine rich repeat containing 61	ND	2.16
Etv1	Ets variant 1	The ETS proteins regulate genes that modulate the cell growth, angiogenesis, migration, proliferation and differentiation	1.68
1700123k08Rik	RIKEN cDNA 1700123K08 gene	ND	1.51
Dcxr	dicarbonyl L-xylulose reductase	Protein plays role oxidation-reduction process	1.47
Sepw1	selenoprotein W, muscle 1	Protein is highly expressed in proliferating myoblasts and suggested to be involved in muscle growth and differentiation by protecting the developing myoblasts from oxidative stress.	1.38
Pibf1	progesterone immunomodulatory binding factor 1	Mediator of progesterone that by acting on the phospholipase A2 enzyme interferes with arachidonic acid metabolism, induces a Th2 biased immune response, and by controlling NK activity exerts an anti-abortive effect	1.37
Skap1	src family associated phosphoprotein 1	The encoded protein plays a critical role in inside-out signaling by coupling T-cell antigen receptor stimulation to the activation of integrins	1.33
Sepn1	selenoprotein N, 1	The function of this gene in mouse is not known, however, mutations in the homologous gene in human are associated with multiminicore disease and rigid spine muscular dystrophy	1.31
Bop1	block of proliferation 1	Block of proliferation 1 (BOP1) protein plays an oncogenic role in hepatocellular carcinoma by promoting epithelial-to-mesenchymal transition.	1.28
Itgb3	integrin beta 3	Integrins are known to participate in cell adhesion as well as cell-surface mediated signalling	1.26
Arl6ip4	ADP-ribosylation factor-like 6 interacting protein 4	Involved in modulating alternative pre-mRNA splicing with either 5 distal site activation or preferential use of 3 proximal site. In case of infection by Herpes simplex virus (HSVI), may act as a splicing inhibitor of HSVI pre-mRNA.	1.22

Supplementary Table S13

The primers used for QPCR analysis

Gene	5'->3', forward	5'-->3',reverse
Poteg	CACTTATCCACCACCCAACC	GTCGTCCCAAAGTCTACCTCAG
Specc1	ATGCTCCATCAACGACTACCC	TAGTCCAGACCCTCCGTTCTC
Xkr7	CCACTTACCACAGAAACAGAACC	ATAGTCGCCTTCCTTTCACCC
Rfx8	TTAAATCCTCCACTTCTCCAGTTCTTG	GGCTCACCTGTGTCTCCAT
Cwc22	GAGAACCTCCACGGAAGTGAA	TCAAGGAACAGTACGACACCAT
Cit	AAACTTCTACCTGTCCAAACAACTC	GCCTCCATCGTTGCTTCT
AurkC	CAGCACCTCAACCAGAAGCATTT	TCCTTGAGCCGAGCCAAGTA
Lgals3bp	AAAGGTTCCAGAGGTTGTCAC	CCAGGGACTCAAGTACAGAAGA
Npb	GTAAGGAGCCTTGCTGCCAG	TCAGGTGCTCTGGCAATCAGT
Ifi27	GCTTCACCGCCTCAGGAAT	GTCCAGCAGCTCAGCAGACT
Cyp21a1	CAGTTCCCAAACCATCTCATCC	TCACCACCCTGAATCCAGAAG
Ifi27l2a	GAGTCCAAGGACCCTGCTGATT	ACTCCCTAAGTTCTTCCTATGCTCT
Phyhip	AGCATGCCAGACCCACTGT	GATGGCATAGTGGTAGGCTGTGT
Aars2	CCACCCAGCCCTACTAACCA	CCCAATGATCACCAAGTCTCCC
Spin2d	CTCCAAGGCTTTCTGGTGT	CAGGAAACCATCAACGGTCAC
Crtam	GAAAGGAGTCATAACACGGTCA	ACCAGCTTCTTCACCATTCAG
Cdk1	CCAGAGCTTGAAGATCCTGAAGA	GTACCGATCTCCAGAAGTGTTG
Ctc1	CTGCTCAGATACAGAAAGAAATCC	GGGCTAATTCACCAAGTGTC
Rif1	AGCAACAGTGAAATCTCGTGG	GCGAACCTTTCTTCATCCGT
Irwd1	CAACACAGATCCTGAAGTGGC	AGTGAGGTAGGTGAAGGCTG
Mis18	CGTTGAAAGTTACACCTTAGGGTC	GGCTGTCAGGACTTCTTCCA
Nbn	GCCAAGCAGACAAGAAACTCAG	GTTATTCCTACATCAACAACGCAG
Mus81	GCATCATAAGCAGCCAGGA	CTGTCAAGAACAAGCCCAGT
Fbox6	GTTGTCAGGAAAGCTTGTGCCA	GAACCCGTGTGCTGAAAGAACC
Chd	ACCAAAGATAGCCTTGTTTCCTG	GCATCCCAACTTACAATACTACTTTCC
Morf4l2	GCTCCTCTCATCTTACTTCTCTG	GAAAGAAGGAACATTGTGGTTGG
Ssrp1	CAGCATTGAAAGGAGGAGTATGG	TCTTCATCAGAGTCAGCATAATCGT
Dnmt3a	GCAGGAGAGGGCAAAGAACAG	CAGCCACTCATCCCGTTTCC
Dnmt3b	TTTAAGTAACCCGTGCTCCA	GGAAATGCTACAAGACCAACAG
Dnmt3l	GCCCTTCTTCTGGATATTCATGGAC	ACTCTTCTTCCTTTGGGGTCAG
Retro- B1	TGGTGGTGCATGCCTTTAAT	CCTGGTGTCCTGGAACTCACT (a)
Retro- B2	GGCTGGTGAGATGGCTCAGT	TACACTGTAGCTGTCTTCAGACA (a)
Retro- BVL1	CTAGAGCATTGGAAGGAGGTGC	ACCACTATAGAATCACAGTACCAC (b)
Retro- L1	GTGGGCGAAGAGGACGATGG	CTTGGCAGTCTAGGCGGGC (c)
Retro-MT	ATGTCTTGGGGAGGACTGTG	AGCCCCAGCTAACCAGAACT (d)
Retro-VL30	CTTCATGACCAAACCCTTCA	GTATGAGTTTCTTCTGCCA (b)

(a) A Allen T, et al 2004 (b) Willard J. Costain et al 2006

(c) Fransen E et al, 1998 (d) Peaston A. et al, 2004

Supplementary Table S13

The primers used for QPCR analysis

chr regions	forward 5'-->3'	reverse 5'-->3'
chr1-Adss	GTTAGTGGTGCTCAACGTCTACGA	TGAGAACTAAGCTGGAGGCGG
chr2-Stk35	TGAAGCAGACAACCACATCCA	TGTTAGTAGATGTAGGAAGACCCA
chr5-Rpl6	GTCAGCCATATCATCACCGAG	ACTACATGCCTTCTCCTTCCA
chr5-Anapc4	AAGTTCCCAAGACGTAATCCTTCC	GTGTTCCTATGTTCTGATCCAGTC
chr6-Ldhb	ATACTGCATTAATCCATCCCATCC	CTCAGAATCCGAATCCGAATCAC
chr7-Klf13	GACTTCCATATGCTGACCCAC	CTTCCTTGCCTCATCTTCTGTC
chr7-Zfp619	TCACCAGCATTACAGAAACCA	GGGACAAAGTTAGCAAAGAAAGG
chr7-Lmo1	CCTGACTCTGCCCATTTCTTCC	ATCCTGCTTTGCCCTCTGAC
chr11-Il1r2	TTTGCTTTGGGACTTCGGTG	TGCTGAATATGAATGAGAGGCGG
chr12-Vrk1	GAACCCACCTCTGTCTCCTTCC	GTCCACATAGAGCAACATCCGT
chr12-Cox16	ACCAATACGCCACTCAGCCAA	GCCAATCGTTTCCGTTTGCCT
chr13-Gm13498	TAGACTGCAAAGGACCCACTG	AAACGATCATCAGGAACGGG
chr13-Scamp1	AGAGTTTCCATCCCAAGCCA	TCTTAACCATTCTGCCTTCTTCC
chr17-Fndc1	ATCTTCTTCAATGTCCTCATCCTC	CTGCTGGAATGGAGTTGTATAGG
chrX-Par	GCGCTCTTCATTTCATTGATTCGT	ACACAGGGACGGACAGACAG
Tel_length	CGGTTTGTTTGGGTTTGGGTTTGGGT<br>TTGGGTTTGGGTT	GGCTTGCCTTACCCTTACCCTTACC<br>CTTACCCTTACCCT
36B4_F	ACTGGTCTAGGACCCGAGAAG	TCAATGGTGCCTCTGGAGATT

Supplemental References

Allen TA, Von Kaenel S, Goodrich JA, Kugel JF. 2004. The SINE-encoded mouse B2 RNA represses mRNA transcription in response to heat shock. *Nature structural & molecular biology* **11**(9): 816-821.

Barr JA, Jones J, Glenister PH, Cattanach BM. 1995. Ubiquitous expression and imprinting of Snrpn in the mouse. *Mammalian genome : official journal of the International Mammalian Genome Society* **6**(6): 405-407.

Bellve AR. 1993. Purification, culture, and fractionation of spermatogenic cells. *Methods in enzymology* **225**: 84-113.

Choufani S, Shapiro JS, Susiarjo M, Butcher DT, Grafodatskaya D, Lou Y, Ferreira JC, Pinto D, Scherer SW, Shaffer LG et al. 2011. A novel approach identifies new differentially methylated regions (DMRs) associated with imprinted genes. *Genome research* **21**(3): 465-476.

Constancia M, Hemberger M, Hughes J, Dean W, Ferguson-Smith A, Fundele R, Stewart F, Kelsey G, Fowden A, Sibley C et al. 2002. Placental-specific IGF-II is a major modulator of placental and fetal growth. *Nature* **417**(6892): 945-948.

Costain WJ, Rasquinha I, Graber T, Luebbert C, Preston E, Slinn J, Xie X, MacManus JP. 2006. Cerebral ischemia induces neuronal expression of novel VL30 mouse retrotransposons bound to polyribosomes. *Brain research* **1094**(1): 24-37.

Dockery L, Gerfen J, Harview C, Rahn-Lee C, Horton R, Park Y, Davis TL. 2009. Differential methylation persists at the mouse Rasgrf1 DMR in tissues displaying monoallelic and biallelic expression. *Epigenetics* **4**(4): 241-247.

Erkek S, Hisano M, Liang CY, Gill M, Murr R, Dieker J, Schubeler D, van der Vlag J, Stadler MB, Peters AH. 2013. Molecular determinants of nucleosome retention at CpG-rich sequences in mouse spermatozoa. *Nature structural & molecular biology* **20**(7): 868-875.

Fransen E, D'Hooge R, Van Camp G, Verhoye M, Sijbers J, Reyniers E, Soriano P, Kamiguchi H, Willemsen R, Koekkoek SK et al. 1998. L1 knockout mice show dilated ventricles, vermis hypoplasia and impaired exploration patterns. *Human molecular genetics* **7**(6): 999-1009.

Gan H, Wen L, Liao S, Lin X, Ma T, Liu J, Song CX, Wang M, He C, Han C et al. 2013. Dynamics of 5-hydroxymethylcytosine during mouse spermatogenesis. *Nature communications* **4**: 1995.

Getun IV, Torres B, Bois PR. 2011. Flow cytometry purification of mouse meiotic cells. *Journal of visualized experiments : JoVE*(50).

Joseph R, Dou D, Tsang W. 1994. Molecular cloning of a novel mRNA (neuronatin) that is highly expressed in neonatal mammalian brain. *Biochemical and biophysical research communications* **201**(3): 1227-1234.

Mahadevaiah SK, Turner JM, Baudat F, Rogakou EP, de Boer P, Blanco-Rodriguez J, Jasin M, Keeney S, Bonner WM, Burgoyne PS. 2001. Recombinational DNA double-strand breaks in mice precede synapsis. *Nature genetics* **27**(3): 271-276.

Page J, de la Fuente R, Manterola M, Parra MT, Viera A, Berrios S, Fernandez-Donoso R, Rufas JS. 2012. Inactivation or non-reactivation: what accounts better for the silence of sex chromosomes during mammalian male meiosis? *Chromosoma* **121**(3): 307-326.

Peaston AE, Evsikov AV, Graber JH, de Vries WN, Holbrook AE, Solter D, Knowles BB. 2004. Retrotransposons regulate host genes in mouse oocytes and preimplantation embryos. *Developmental cell* **7**(4): 597-606.

Peters AH, Plug AW, van Vugt MJ, de Boer P. 1997. A drying-down technique for the spreading of mammalian meiocytes from the male and female germline. *Chromosome research : an international journal on the molecular, supramolecular and evolutionary aspects of chromosome biology* **5**(1): 66-68.

Plass C, Shibata H, Kalcheva I, Mullins L, Kotelevtseva N, Mullins J, Kato R, Sasaki H, Hirotsune S, Okazaki Y et al. 1996. Identification of Grf1 on mouse chromosome 9 as an imprinted gene by RLGS-M. *Nature genetics* **14**(1): 106-109.
Russell LD. 1990. *Histological and histopathological evaluation of the testis*. Clearwater, Fl, Cache River Press.
Szabo PE, Mann JR. 1995. Allele-specific expression and total expression levels of imprinted genes during early mouse development: implications for imprinting mechanisms. *Genes & development* **9**(24): 3097-3108.
Vallet-Erdtmann V, Tavernier G, Contreras JA, Mairal A, Rieu C, Touzalin AM, Holm C, Jegou B, Langin D. 2004. The testicular form of hormone-sensitive lipase HSLtes confers rescue of male infertility in HSL-deficient mice. *The Journal of biological chemistry* **279**(41): 42875-42880.
